# Supplementary material for: Preclinical evaluation of nitrated α-synuclein-specific CAR-Treg therapy in Parkinson’s disease
Source: Cell Rep Med. 2026 Aug 12;7(9):102983. doi: 10.1016/j.xcrm.2026.102983 (PMC13589471; doi:10.1016/j.xcrm.2026.102983)
Supplement: Supplementary file 1 [file mmc1.pdf]

Cell Reports Medicine, Volume *7*

Supplemental information

Preclinical evaluation of nitrated α-synuclein-specific CAR-Treg therapy in Parkinson's disease

Valentina Ugalde, Daniela Elgueta, Sandra Espinoza, Ornella Chovar-Vera, Ernesto López, Alejandra Catenaccio, Francisca Álvarez-Astudillo, Dafne Franz, Vanesa Sanchez-Guajardo, María Inés Becker, Diego Figueroa, Zulmary Manjarres, Sofía Hidalgo, Álvaro Lladser, Cheril Tapia-Rojas, Alejandra Loyola, Sergio A. Quezada, Roque Villagra, and Rodrigo Pacheco

Figure S1. Working model hypothesis and aim.

(A–G) Scheme representing the proposed working model of how T cells promote neuroinflammation and neurodegeneration in Parkinson's disease (PD).

(A) Oxidative stress triggers the generation of pathogenic forms of **α**Syn, (B) which aggregate into oligomers and are released into the extracellular space. These **α**Syn oligomers stimulate toll-like receptors on microglia and antigen-presenting cells (APCs), inducing an initial and local inflammatory response.

(C) APCs capture pathogenic forms of **α**Syn, process them, and present their fragments on MHC molecules while migrating to the lymph nodes.

(D) In the lymph nodes, APCs activate $CD8^+$ and $CD4^+$ T cells specific for **α**Syn-derived antigens, which subsequently infiltrate the brain.

(E) $CD4^+$ T cells are re-stimulated by microglia presenting **α**Syn-derived antigens, inducing the production of inflammatory cytokines by T cells, particularly TNF**α** and IFN**γ**. These cytokines promote the production of neurotoxic factors by microglia, thereby favouring neuronal death.

(F) In addition, oxidative stress produced by microglia induces further oxidation and the generation of pathogenic forms of **α**Syn.

(G) Recognition of neurons presenting **α**Syn-derived antigens on MHC class I molecules by $CD8^+$ T cells may induce the release of cytotoxic vesicles, leading to neuronal death.

(H) The aim: we propose to develop regulatory T cells (Treg) expressing chimeric antigen receptors (CAR) specific for the recognition of pathogenic forms of **α**Syn, which, upon antigen recognition, would dampen inflammatory processes and attenuate the progression of neuroinflammation and neurodegeneration.

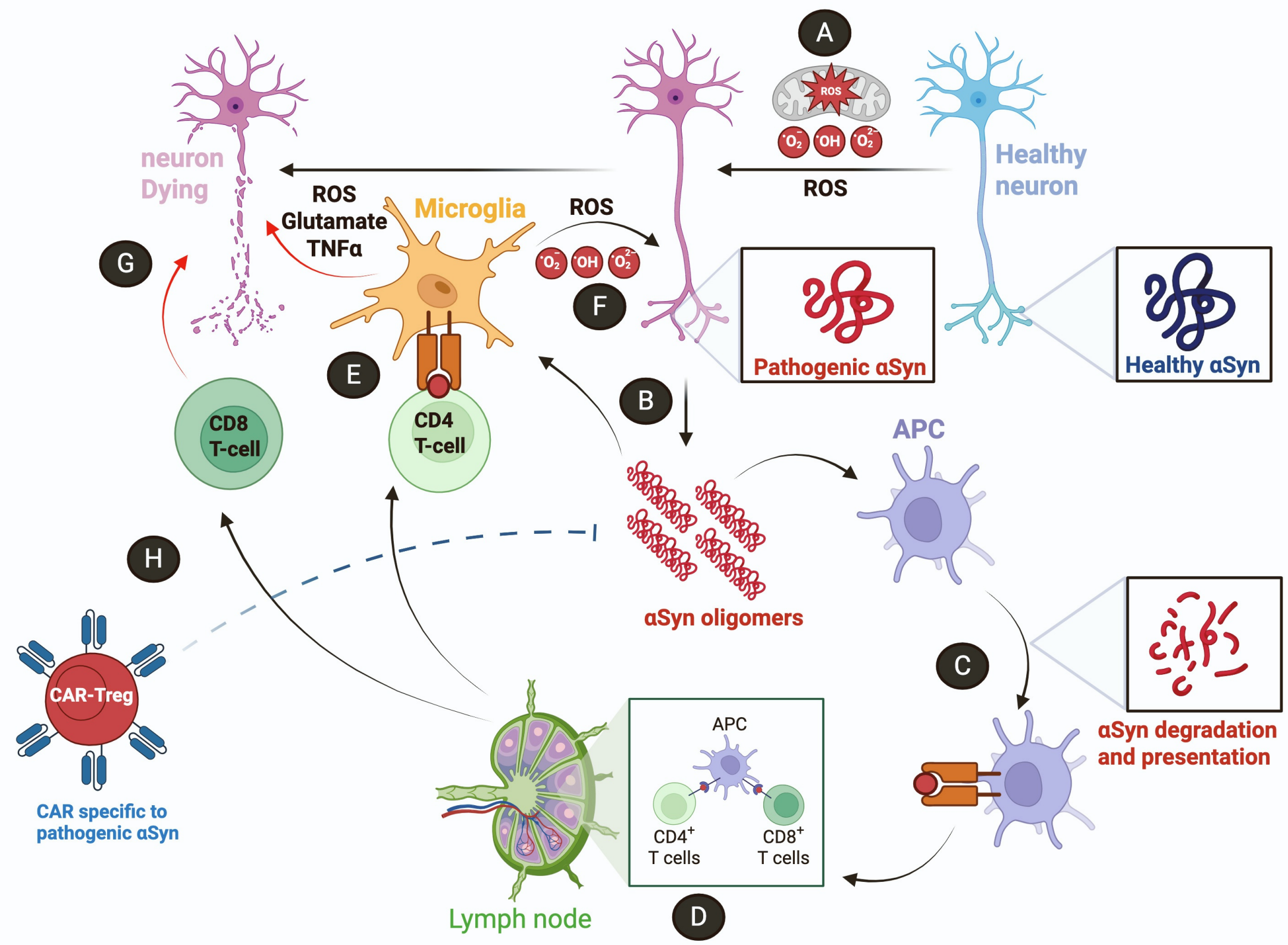

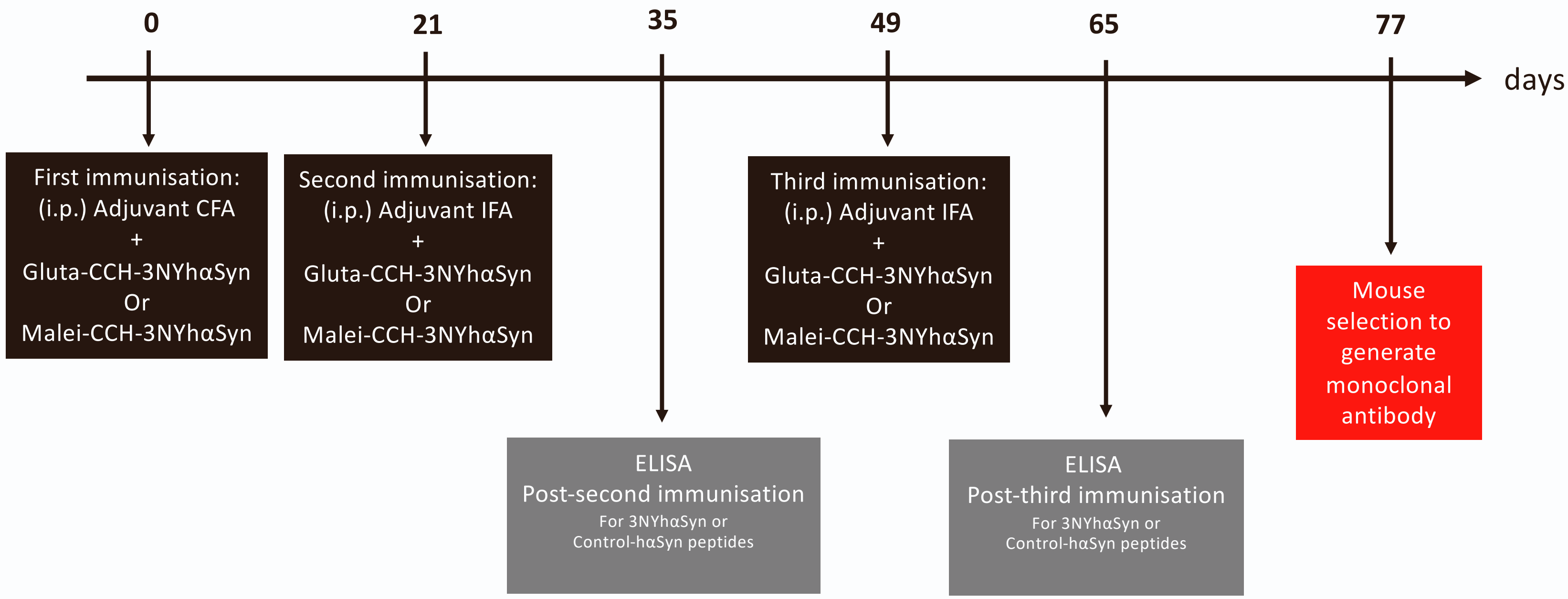

Figure S2. Strategy used for generation of anti-3NYhαSyn mouse polyclonal antibodies. Related to Figure 1 A and B. Scheme representing a timeline of immunisation and analyses to detect anti-3NYhαSyn pAb in serum. The i.p. immunisation with glutaraldehyde CCH-3NYhαSyn (Gluta-CCH-3NYhαSyn; 250 µg of immunogen per mouse), maleimide CCH-3NYhαSyn (250 µg of immunogen per mouse) or PBS was performed using CFA as adjuvant, whereas the second and third immunisation were performed using IFA instead CFA. ELISA analyses were carried out by activating plates with 10 µg/mL of 3NYhαSyn or control-hαSyn peptides after the second and third immunisation. Serial dilutions of serum were assessed starting at 1:10. Immunisations are shown in black boxes, analyses in grey boxes and final outcome in a red box.

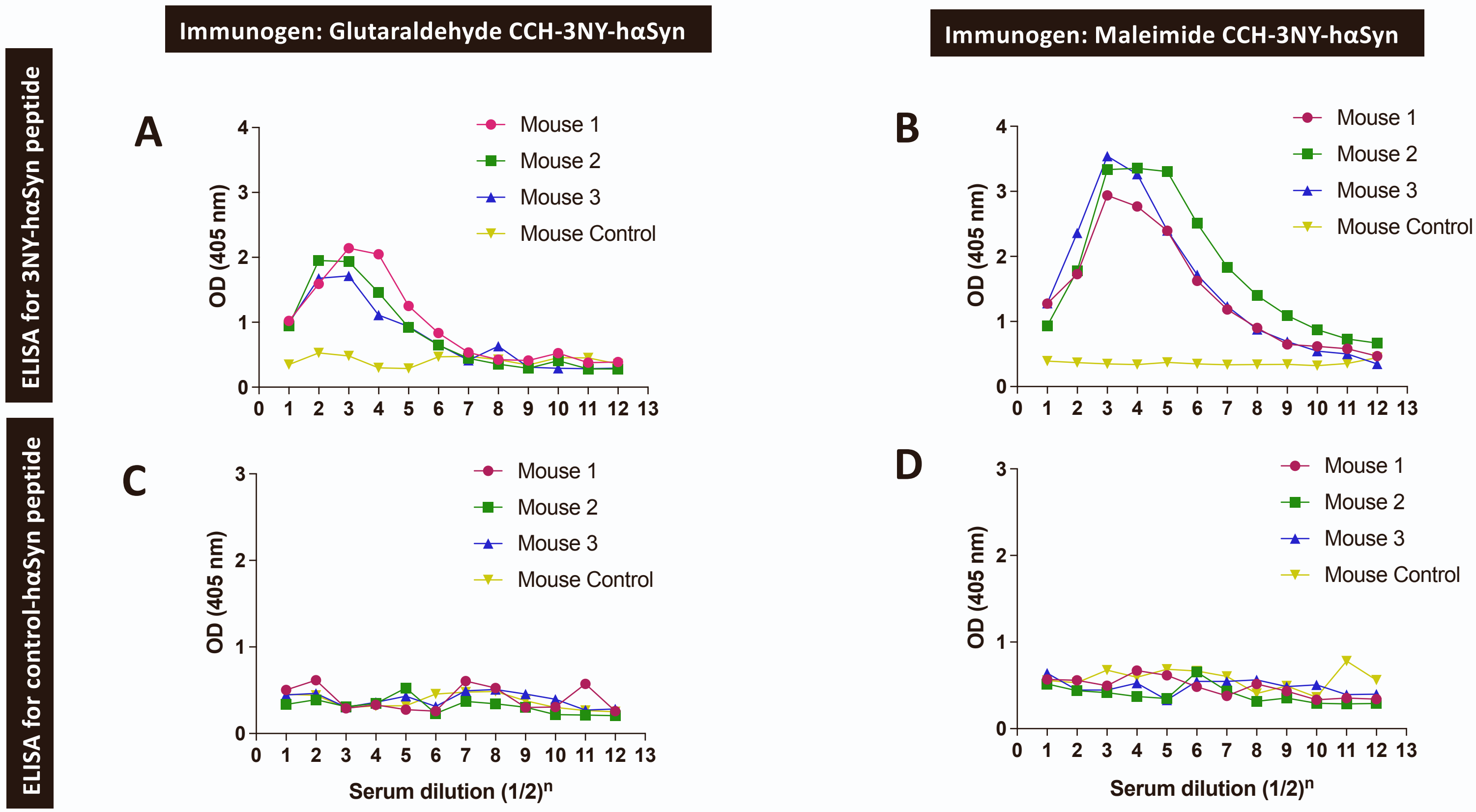

Figure S3. Detection of anti-3NYhαSyn pAb by ELISA to 3NYhαSyn and control-hαSyn peptides after the second immunisation of mice (at day 35). Related to Figure 1 A and B. (**A**) Serum from animals immunised with glutaraldehyde CCH-3NYhαSyn conjugate were assessed for 3NYhαSyn peptide. (**B**) Serum from animals immunised with maleimide CCH-3NYhαSyn conjugate were assessed for 3NYhαSyn peptide. (**C**) Serum from animals immunised with glutaraldehyde CCH-3NYhαSyn conjugate were assessed for control-hαSyn peptide. (**D**) Serum from animals immunised with maleimide CCH-3NYhαSyn conjugate were assessed for control-hαSyn peptide. (A-D) The optic density (O.D.) was detected for serial dilutions of serum obtained from immunised mice (mouse 1-3) or from control mouse (injected with PBS and adjuvant).

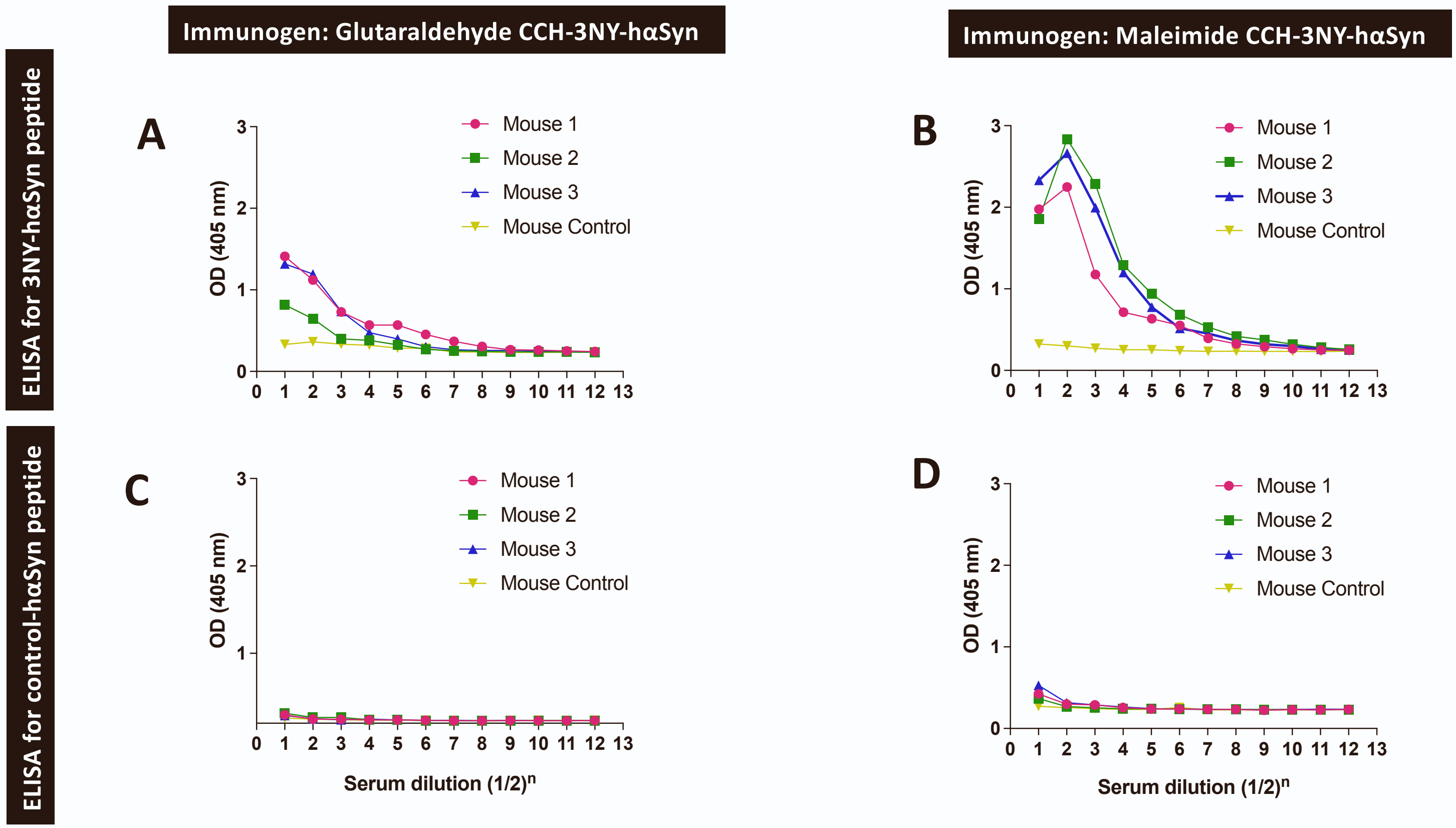

Figure S4. Detection of anti-3NYhαSyn pAb by ELISA for 3NYhαSyn and control-hαSyn peptides after the third immunisation of mice (at day 65). Related to Figure 1 A and B. (**A**) Serum from animals immunised with glutaraldehyde CCH-3NYhαSyn conjugate were assessed for 3NYhαSyn peptide. (**B**) Serum from animals immunised with maleimide CCH-3NYhαSyn conjugate were assessed for 3NYhαSyn peptide. (**C**) Serum from animals immunised with glutaraldehyde CCH-3NYhαSyn conjugate were assessed for control-hαSyn peptide. (**D**) Serum from animals immunised with maleimide CCH-3NYhαSyn conjugate were assessed for control-hαSyn peptide. (A-D) The optic density (O.D.) was detected for serial dilutions of serum obtained from immunised mice (mouse 1-3) or from control mouse (injected with PBS and adjuvant).

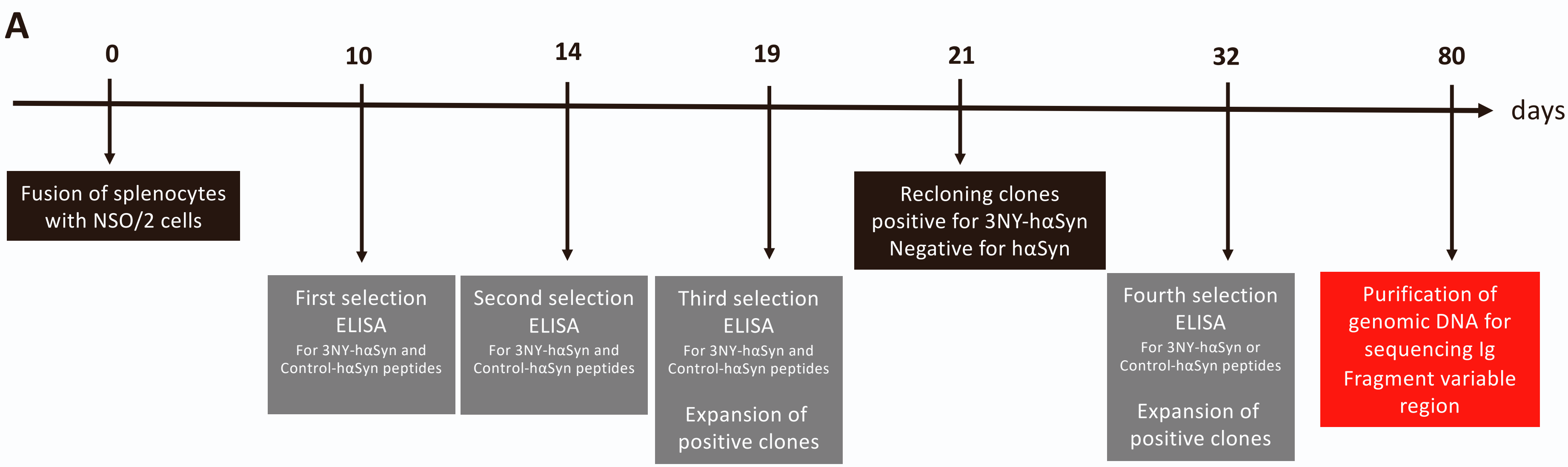

B

Clone	ELISA for 3NY-hαSyn (O.D; mean ± SEM)	ELISA for control-hαSyn (O.D; mean ± SEM)	Heavy chain Isotype	Light chain Isotype
1A 11	2.78 ± 0.57	0.27 ± 0.01	IgG1	Igκ
8E 7	3.34 ± 0.16	0.26 ± 0.01	IgA	Igκ

Figure S5. Generation of anti-3NY-hαSyn mAb. Related to Figure 1 A and B. (**A**) Scheme representing a timeline of generation of hybridomas, expansion and selection of clones by ELISA analyses to detect anti-3NY-hαSyn mAb in hybridomas supernatant. Actions are shown in black boxes, analyses in grey boxes and final outcome in a red box. (**B**) Table showing the two hybriboma clones displaying supernatants with high reactivity to 3NY-hαSyn peptide and negative reactivity to control-hαSyn peptide. Values represent average optic density. The isotype for each clone was determined by the Rapid Antibody Isotyping kit.

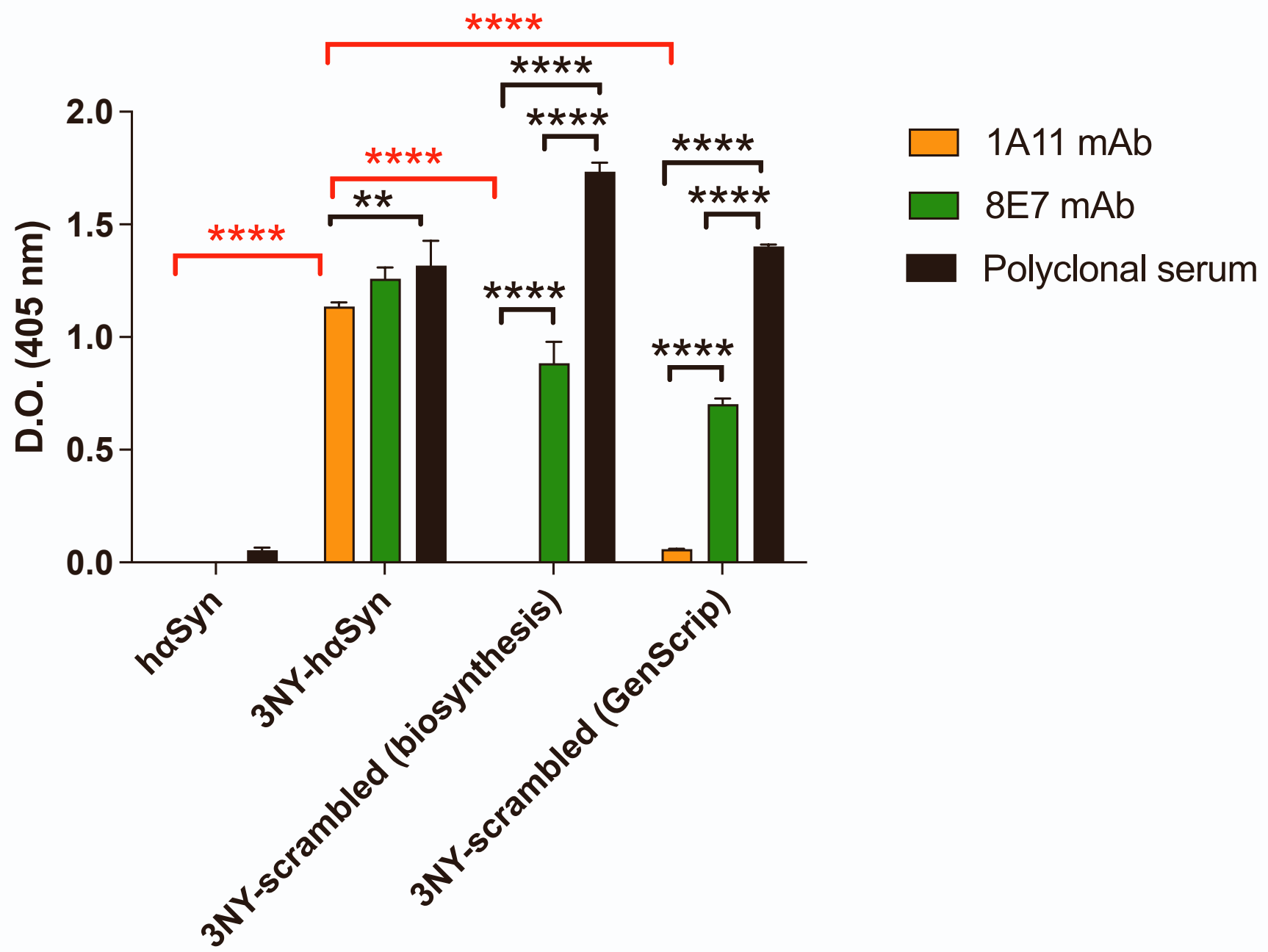

Figure S6. Evaluating the specificity of 1A11 and 8E7 mAbs by ELISA. **Related to Figure 1 A and B.** Serum from animals immunised with maleimide CCH-3NY-hαSyn conjugate (diluted 1:25 in PBS; black bars) or undiluted supernatants from 1A11 (orange bars) or 8E7 (green bars) hybridomas were assessed for reactivity with peptides hαSyn, 3NY-hαSyn or 3NY-scrambled (obtained from biosynthesis or from GenScript). Detection of reactivity was performed by ELISA using a secondary anti-mouse Ig (H+L) antibody conjugated to phosphatase alkaline followed by the addition of p-nitrophenyl phosphate. The optic density was quantified at 405 nm. Values are mean with SEM from triplicates. **, $p<0.01$; ****, $p<0.0001$ by two-way ANOVA followed by Tukey's posthoc test. Differences obtained between different antibodies using the same antigen are indicated in black asterisks/brackets, whilst differences obtained for 1A11 mAb using different antigens are indicated in red asterisks/brackets.

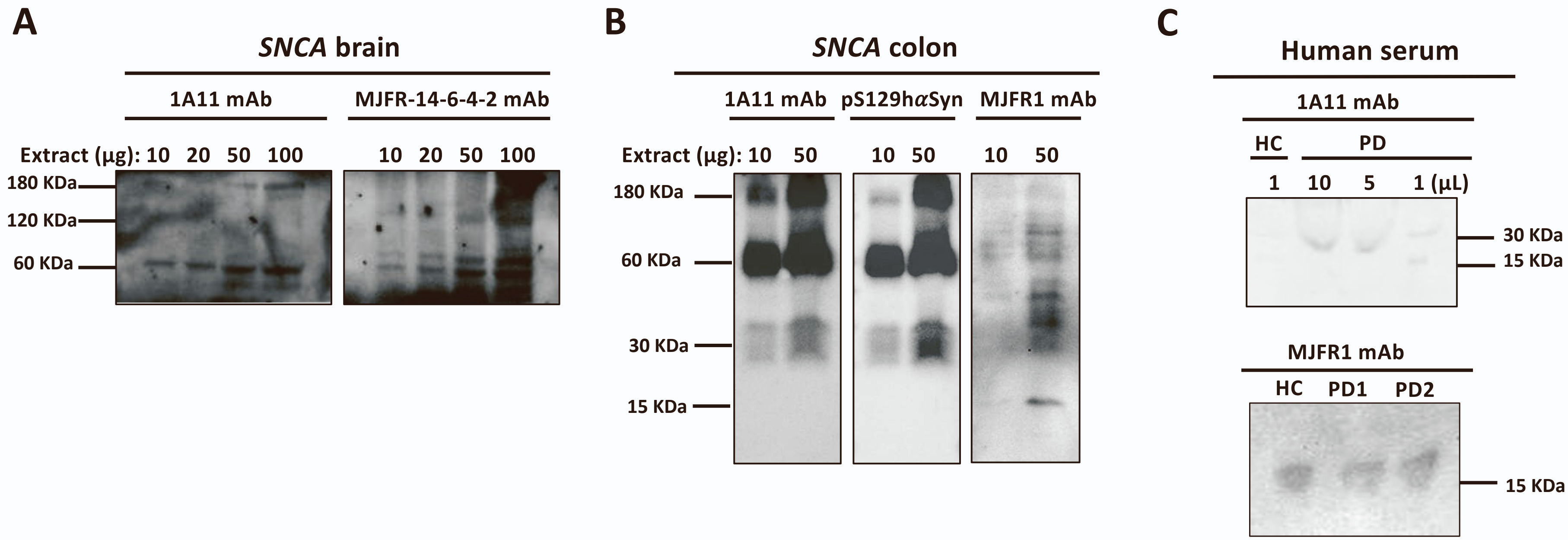

Figure S7. Characterising the 1A11 mAb antigen recognition in biological samples. Related to Figure 1. (**A**) Brain lysate obtained from 20-week-old *SNCA* mice was loaded in increasing amounts and separated in SDS-PAGE 10% followed by western blot analysis using the 1A11 mAb or the MJFR-14-6-4-2 mAb (specific for recognition of h**α**Syn aggregates; Abcam, cat# ab209538). (**B**) Colon lysate obtained from 20-week-old SNCA mice was loaded in increasing amounts and separated in SDS-PAGE 12,5% followed by western blot analysis using the 1A11 mAb, pSer129-h**α**Syn (Biolegend) or the MJFR1 mAb (specific for recognition of monomeric **α**Syn; Abcam, cat# ab138501). (**C**) Serum samples obtained from Parkinson's disease patients (PD) or from heathy controls (HC) were loaded and separated in SDS-PAGE 18% followed by western blot analysis using the 1A11 mAb or the MJFR1 mAb. Approximate molecular weights are indicated.

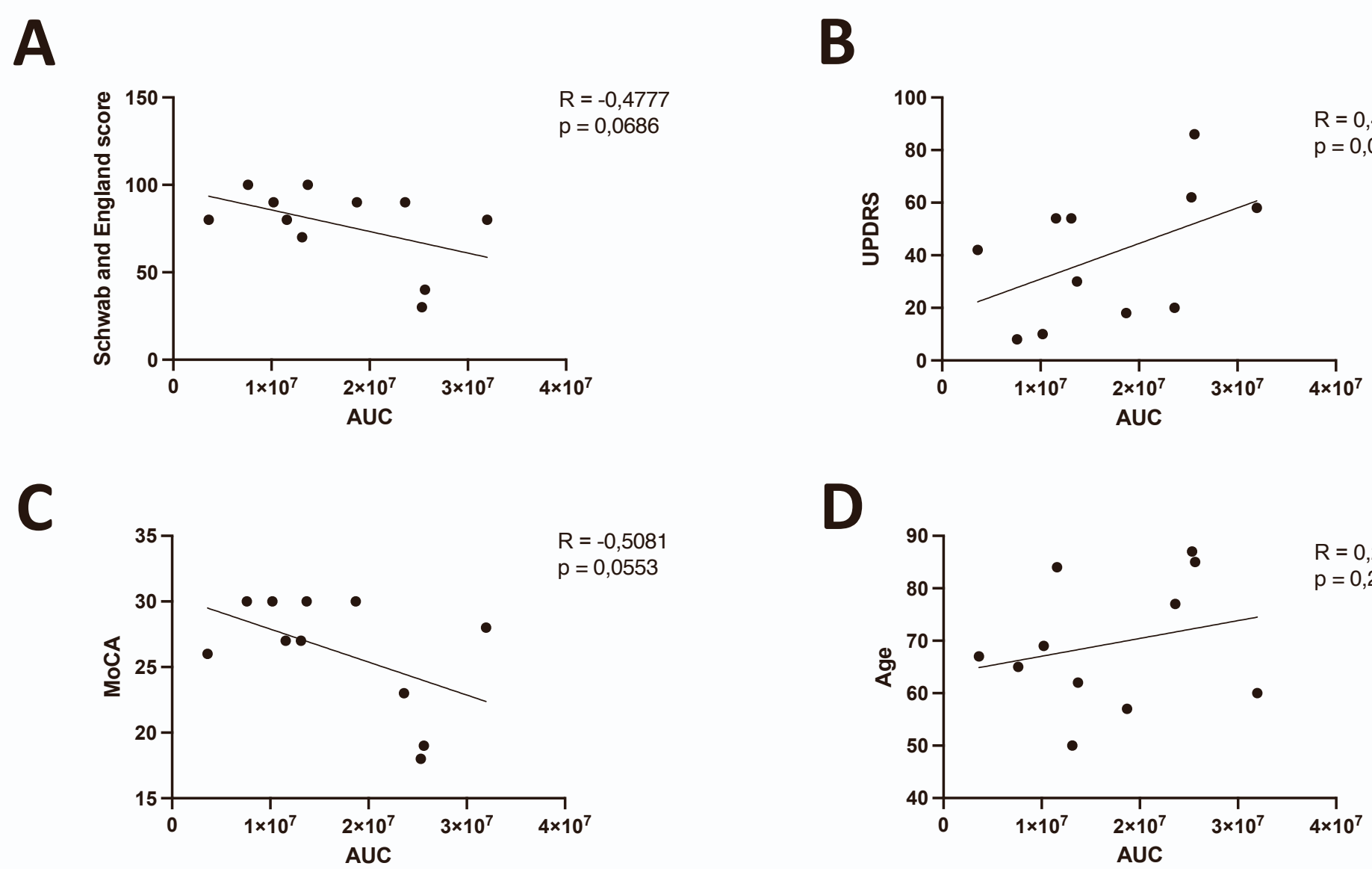

Figure S8. Correlation analysis of the immunoreactivity of the 1A11 mAb with clinical score and age of PD patients. **Related to Figure 1 E.** Dot blot immunoreactivity of 1A11 mAb in serum from PD patients versus clinical score determined by Schwab and England score (**A**), UPDRS (**B**), MoCA test (**C**), and versus the age (**D**). R values are indicated. Each symbol represents data from an individual subject. P values are indicated, as calculated by the Pearson's correlation test.

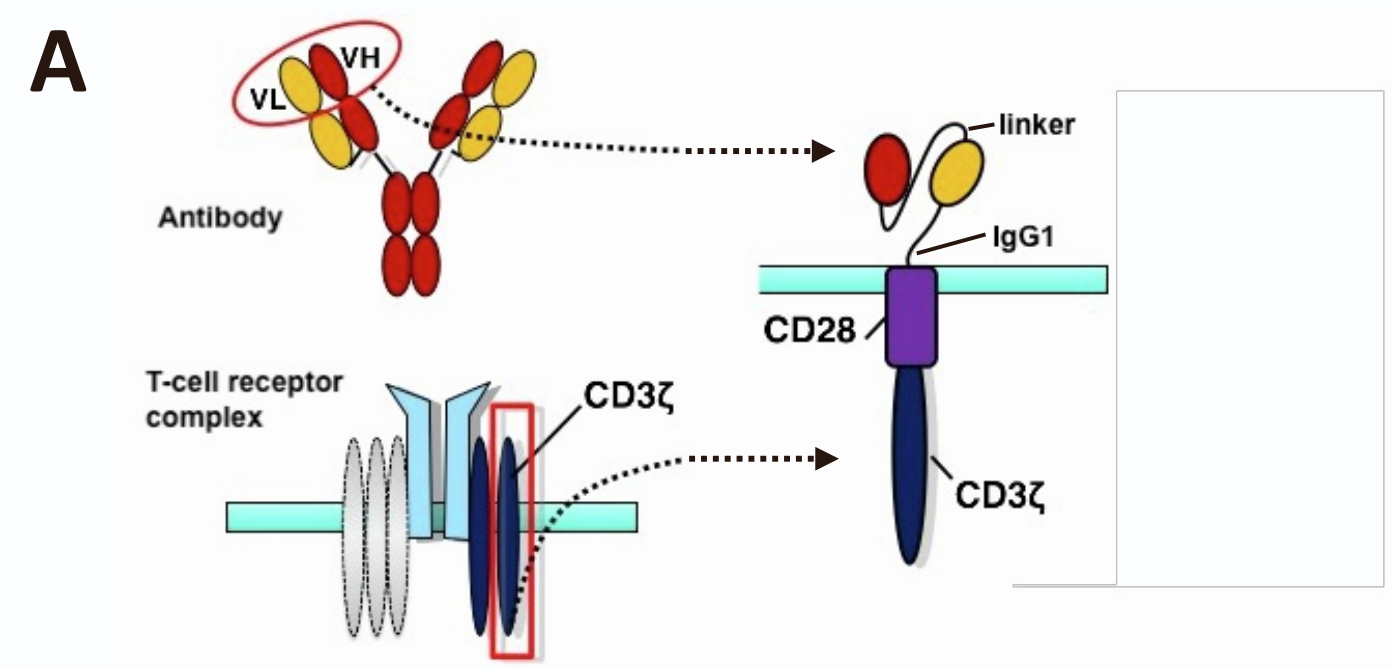

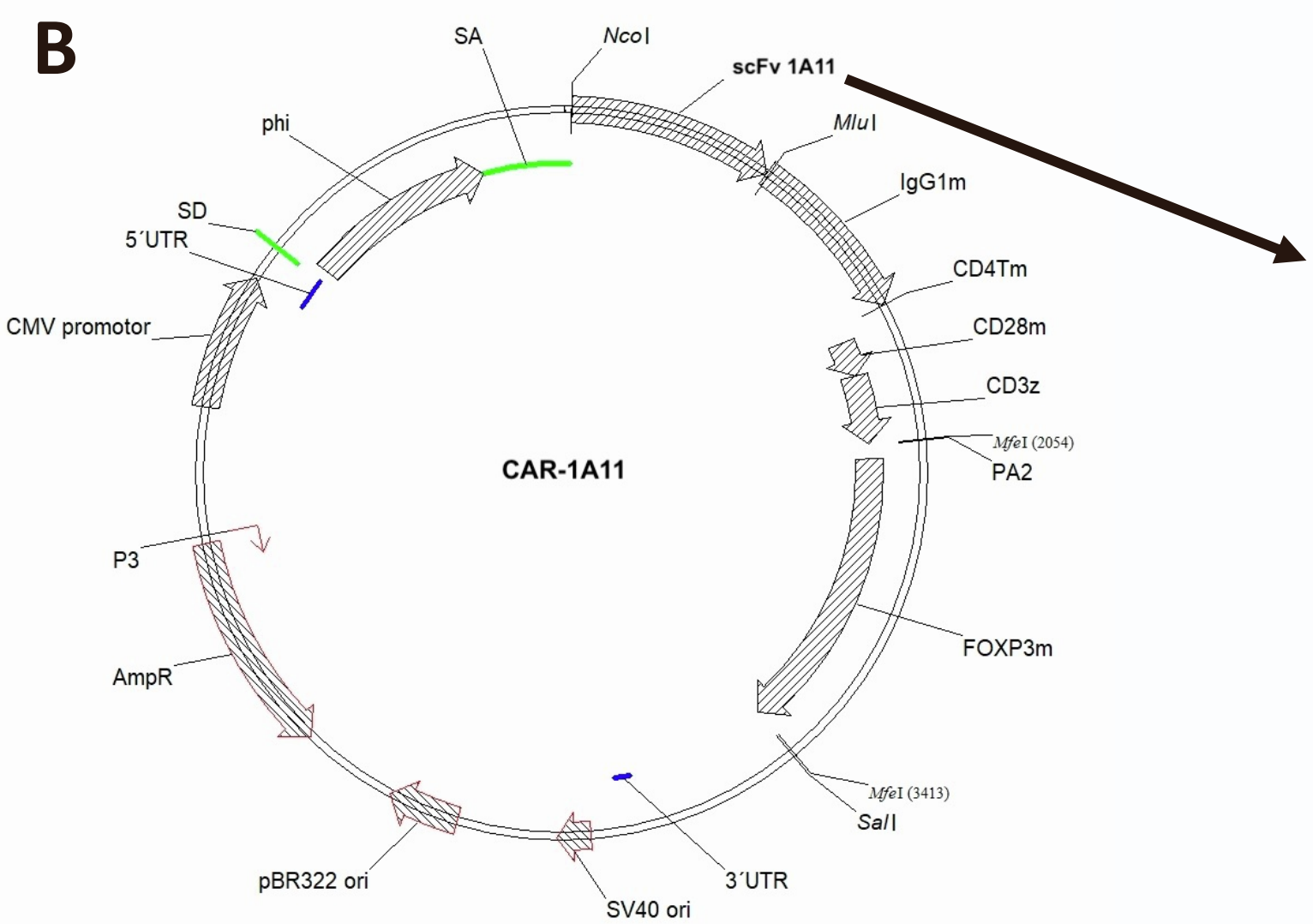

Region	Nucleotide sequence
3’-VL	gatgt…….aacgg
Spacer	ggagg……gatct
VH-5’	gaggt…….cttct

Region	Nucleotide sequence
3’-VH	gaggt…….cttct
Spacer	ggagg……gatct
VL-5’	gatgt…….aacgg

Figure S9. Generation of vectors encoding the CAR1A11. Related to Figure 2. (**A**) Scheme of a Chimeric Antigen Receptor (CAR). (**B**) The left panel shows a map of the vector built to generate regulatory T-cells expressing the CAR1A11. The DNA encoding for a single chain variable fragment (scFv; the antigen-recognising site) of the 1A11 mAb was inserted into a pBullet vector CAR cassette including an extracellular mouse IgG1 hinge domain, CD4 transmembrane domain (CD4Tm) and intracellular CD28 and CD3z moieties responsible for costimulatory and activation signals, respectively. The murine Foxp3 gene was inserted into the construct at the 3' side of the CAR1A11 gene and separated by a 2A peptide. All genes were under the control of a strong promoter (cytomegalovirus; CMV). The right panel shows the abbreviated nucleotide sequences of VH (red) and VL (yellow) from the 1A11 mAb. A spacer sequence required for generating scFv is also indicated (grey). The scFv was arranged in two versions: 3’-VL-spacer-VH-5’ and 3’-VH-spacer-VL-5’.

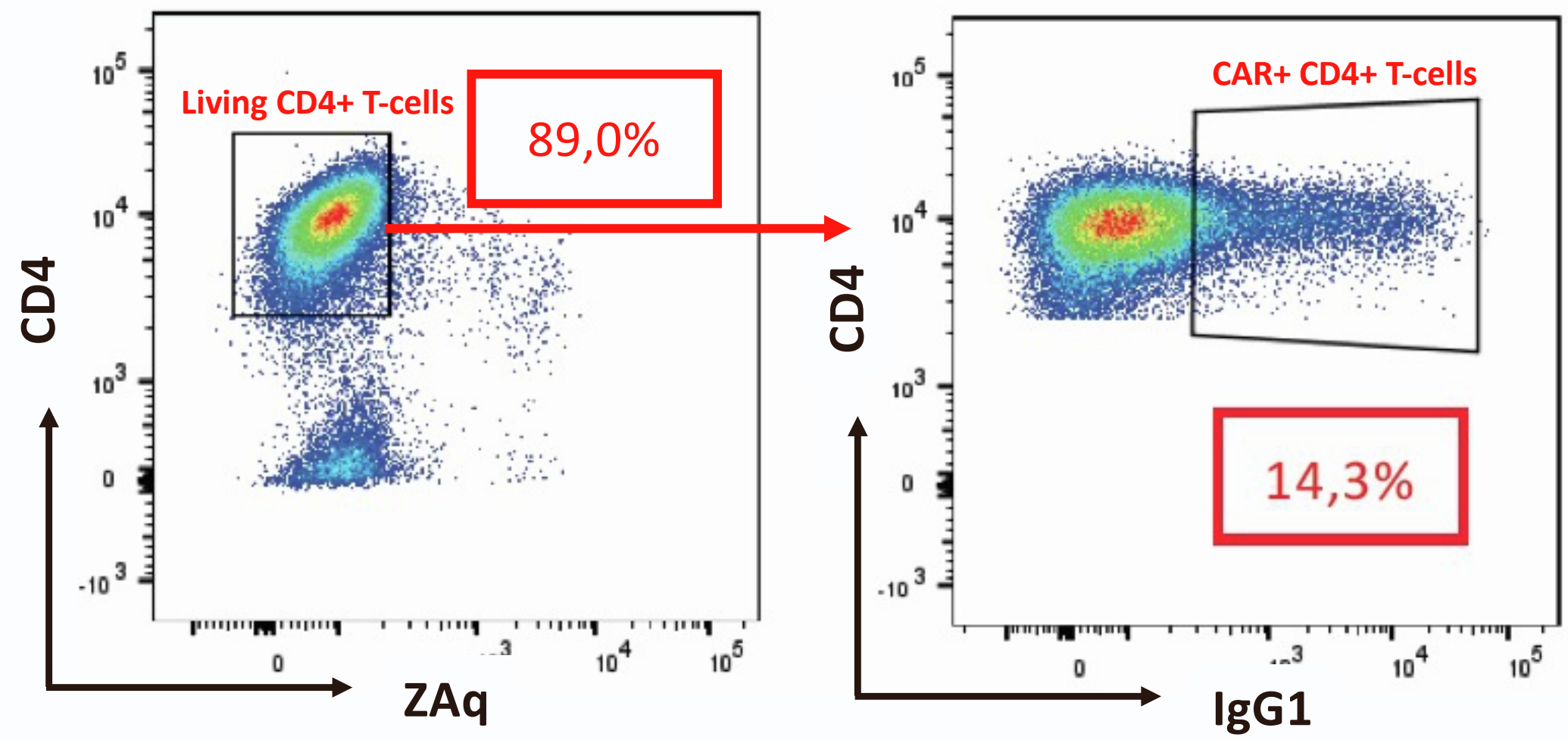

Figure S10. Transduction of CD4⁺ T-cells with viral vectors encoding for CARs. **Related to Figure 2.** Total $CD3^+$ $CD4^+$ T-cells were isolated from the spleen of WT mice and activated with αCD3/αCD28-coated dynabeads (dynabead-to-cell ratio 1:1) in the presence of IL-2 (1 µg/mL). After 24h, cells were spinoculated over retroviral particles encoding the CAR1A11. The expression of the CAR was evaluated as the extent of surface anti-IgG1 immunoreactivity in living (ZAq^-) $CD4^+$ T-cells. Representative dot-plots are shown. The percentage of cells in the indicated region is shown in red.

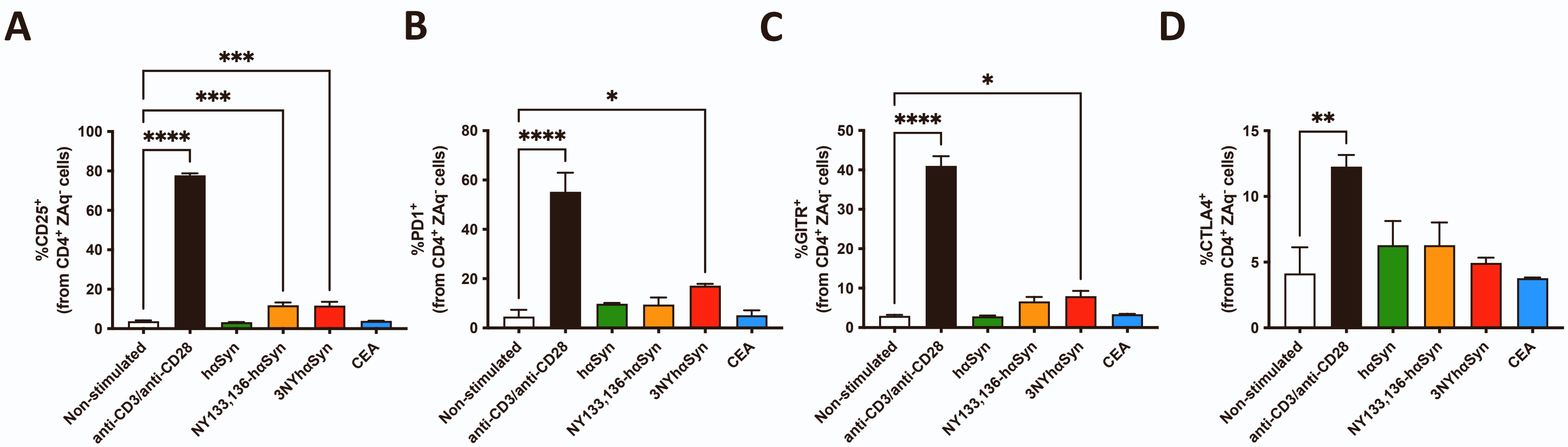

Figure S11. **Characterising CAR-Treg phenotype in response to antigen recognition. Related to Figure 2.** 96-well plates were incubated with hαSyn$_{111-140}$-derived peptides (at 1 µg/mL), including unmodified hαSyn (green), NY133,136-hαSyn (orange), and 3NY-hαSyn (red). As controls, plates were incubated with only vehicle (white), carcinoembrionary antigen (CEA;1 µg/mL, blue), or anti-CD3 (1 µg/mL) plus anti-CD28 (2 µg/mL) (black). Afterwards, CAR-Treg (transduced with the CAR1A11 in the VH-VL conformation; $5x10^4$ cells/well) were incubated with the different antigens for 24 h and the surface expression of CD25 (**A**), PD1 (B), GITR (C) and CTLA4 (D) were determined in live $CD4^+$ T-cells ($CD4^+$ ZAq^- population) by flow cytometry. Data represent the mean ± SEM of triplicates from one out of three experiments with similar results.

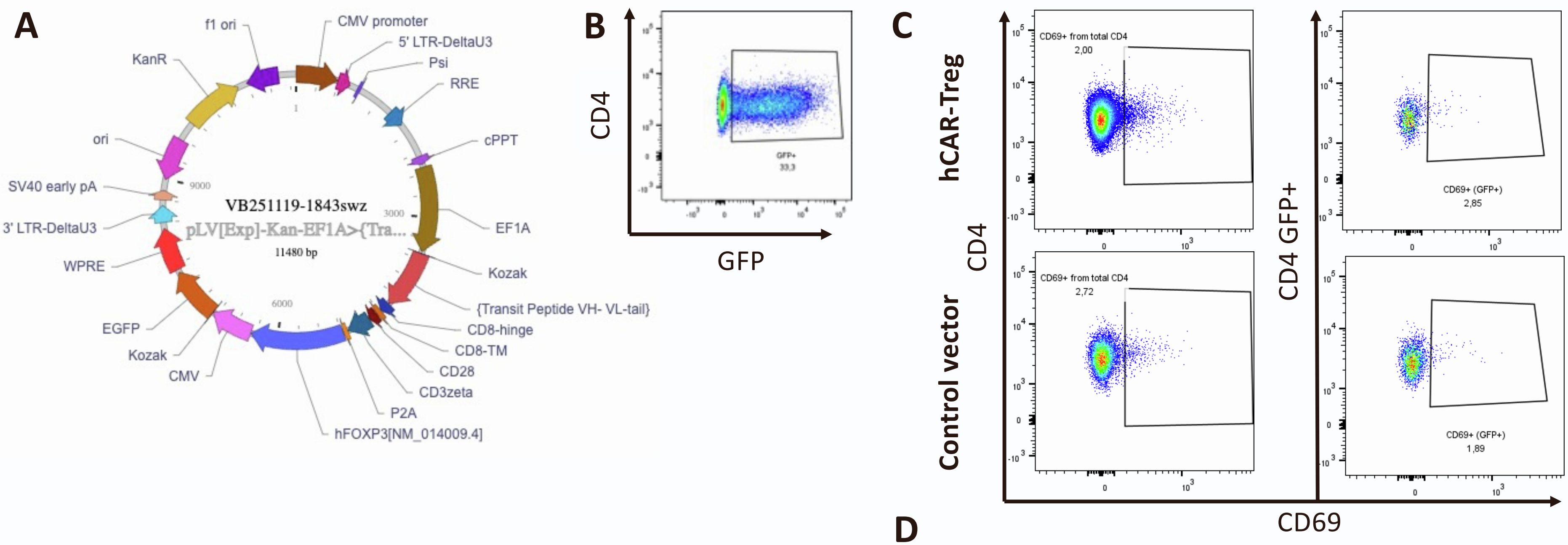

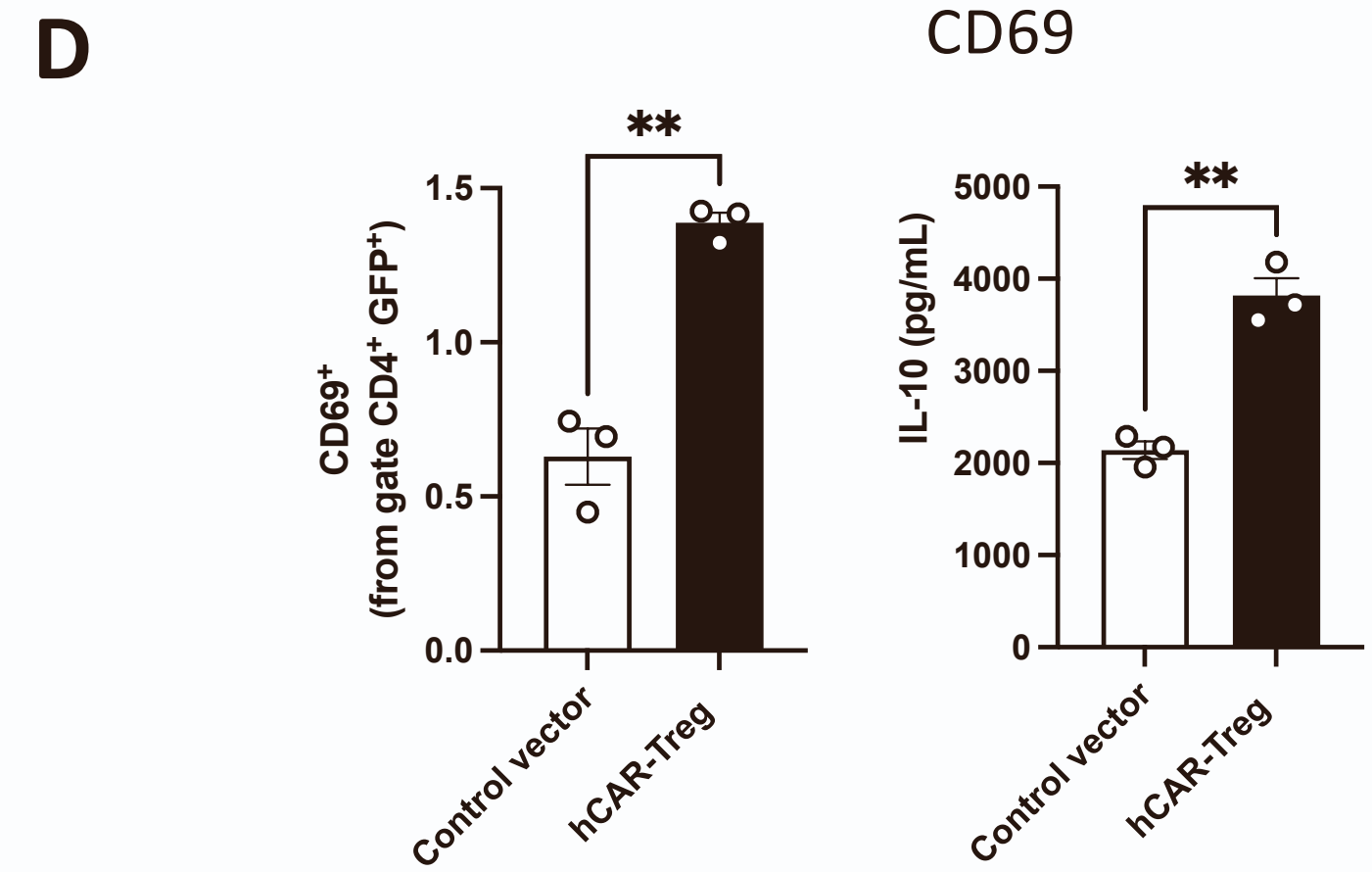

Figure S12. **Human CAR-Treg (hCAR-Treg) becomes activated and release IL-10 in response to antigen recognition. Related to Figure 2.** Human CD4+ T-cells were isolated by cell-sorting from PBMC and then transduced with a lentiviral vector encoding for the human version of the CAR-1A11 (in the VH-VL conformation) bearing the CD3z and CD28 signalling motifs and the human Foxp3 gene. (**A**) Illustration of the lentiviral vector encoding the hCAR, Foxp3 and GFP. (**B**) Representative dot-plot showing the transduction efficiency as determine by the extent of GFP^+ cells in the $CD4^+$ T-cell population by flow cytometry. (C-D) Human CD4+ T-cells transduced with the hCAR-Treg vector or with a control vector (encoding GFP only) were incubated with the 3NY-hαSyn peptide (1 µg/mL) for 24 h. The extent of activation was assessed by the surface CD69 expression and the production of IL-10 was determined in the culture supernatant as a measure of Treg function. (**C**) Representative dot-plots showing the extent of the surface CD69 expression on the CD4+ or the CD4+ GFP+ population. (D) The left panel shows the quantification of the relative CD69 expression in the CD4+ GFP+ population determined by flow cytometry. The right panel shows the quantification of IL-10 in the culture supernatant. Data represent the mean ± SEM of triplicates from one out of three experiments with similar results. **, p<0.01 by two-tailed unpaired Student's t-test.

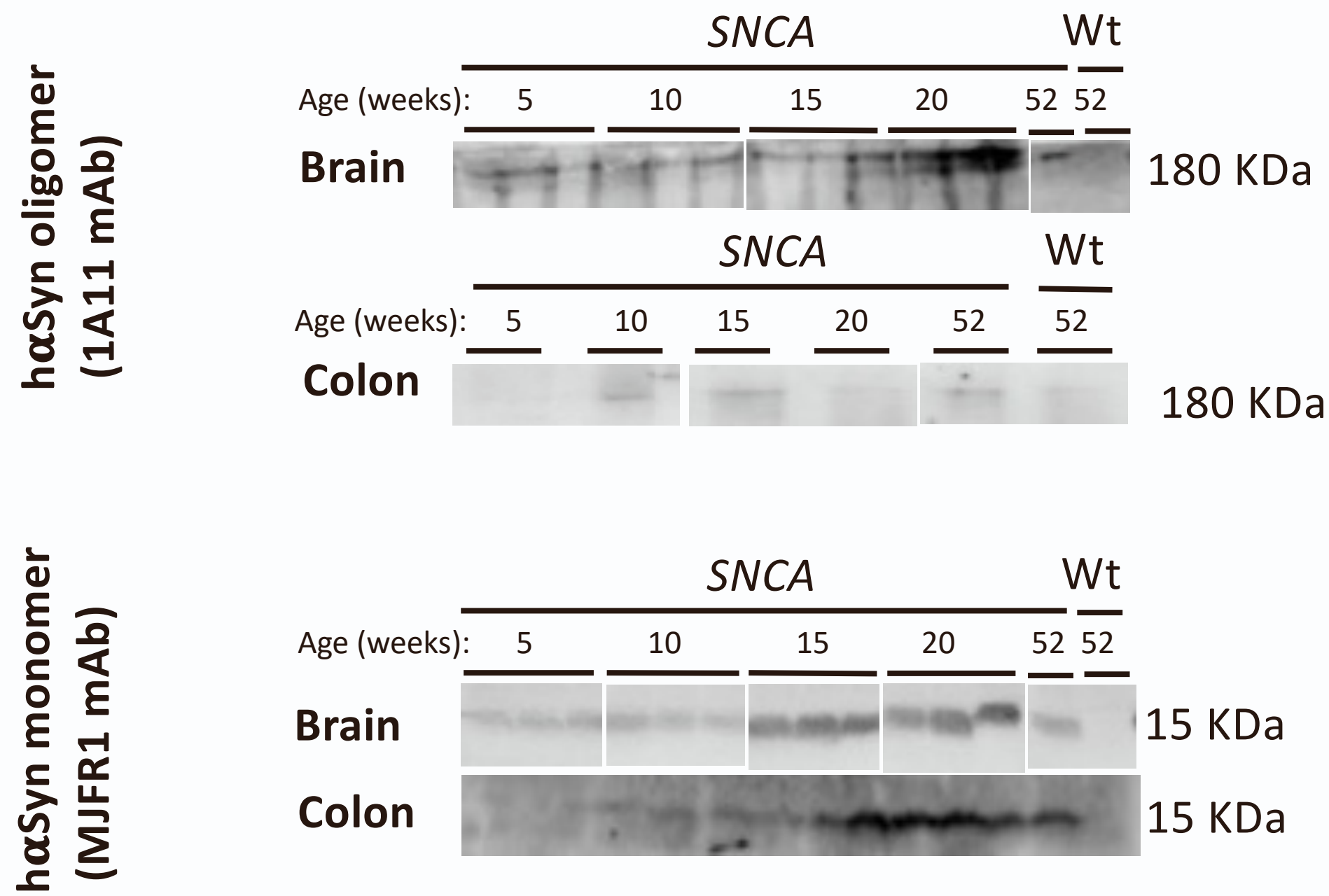

Figure S13. Kinetic analysis of human αSyn expression in brain and colon of *SNCA* mice. Related to Figures 1 and 2. Brain and colon lysates obtained from *SNCA* mice of different ages, or from 1-year old wild-type (Wt) mice were separated in SDS-PAGE 10% followed by western blot analysis using the 1A11 mAb or the MJFR1 mAb (specific for recognition of monomeric **α**Syn; Abcam, cat# ab138501).

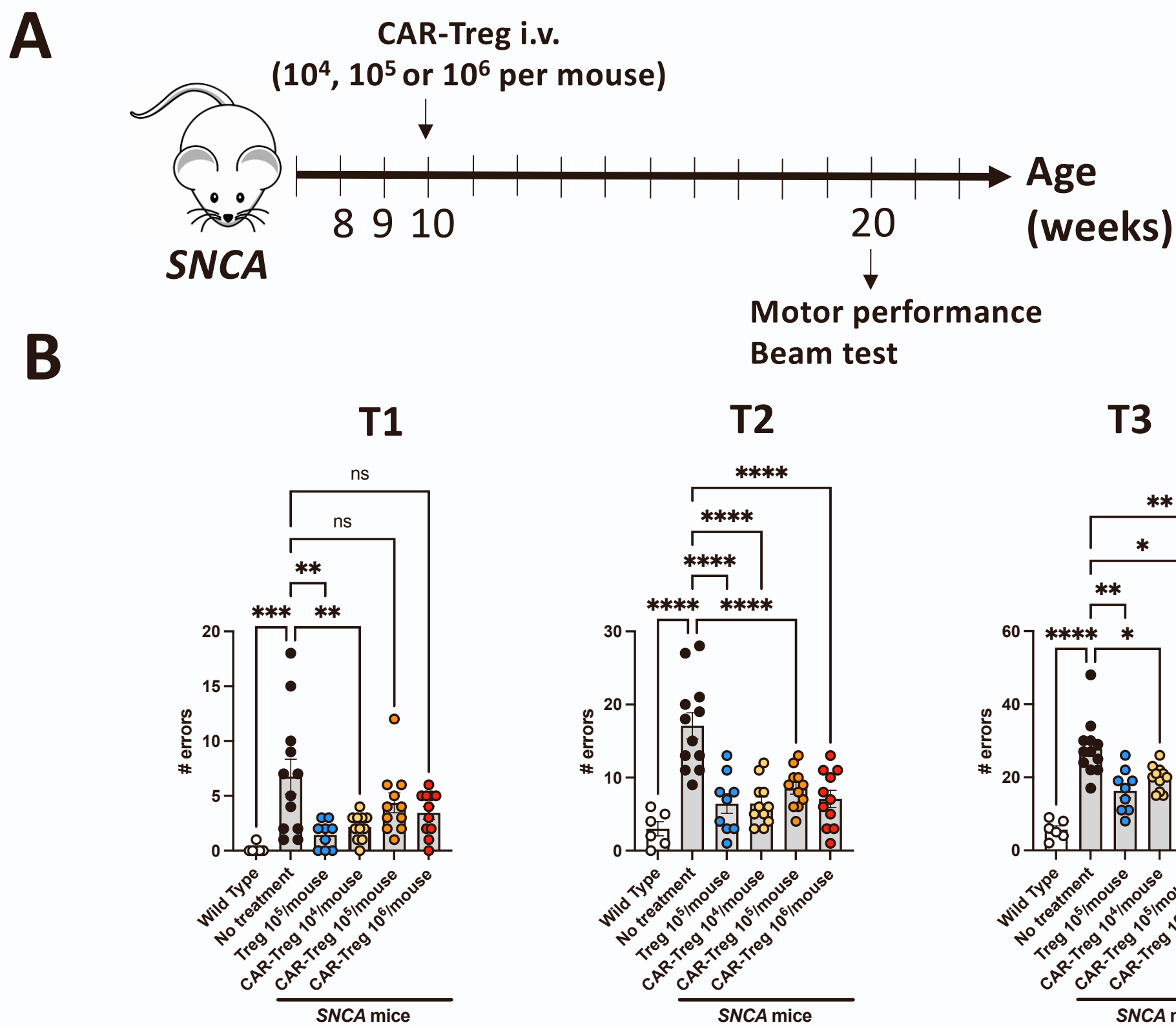

Figure S14. A single injection of the CAR-Treg therapy improves the motor decline in *SNCA* mice. Detailed beam test analysis. Related to Figure 3. Ten weeks old *SNCA* mice were treated with a single i.v. injection of CAR-Treg expressing the CAR1A11 in the VH-VL conformation (10^4 cells/mouse, yellow symbols; 10^5 cells/mouse, orange symbols; 10^6 cells/mouse, red symbols), or polyclonal Treg (10^5 cells/mouse, blue symbols). Non-treated *SNCA* mice (black symbols) were used as controls. Age matched wild-type mice (white symbols) were also evaluated as controls. The motor performance was evaluated at 20 weeks of age by the beam test. (**A**) Illustration of the experimental strategy. (**B**) The number of errors was quantified in each single tract (T1-T4). Each symbol represents an individual mouse (n = 6-12 mice per group). Mean ± SEM are shown. *, p<0.05; **, p<0.01; ***, p<0.001; ****, p<0.0001 compared to the control *SNCA* mice without treatment (black symbols) by one-way ANOVA followed by Dunnett's *post-hoc* test. ns, non-significant.

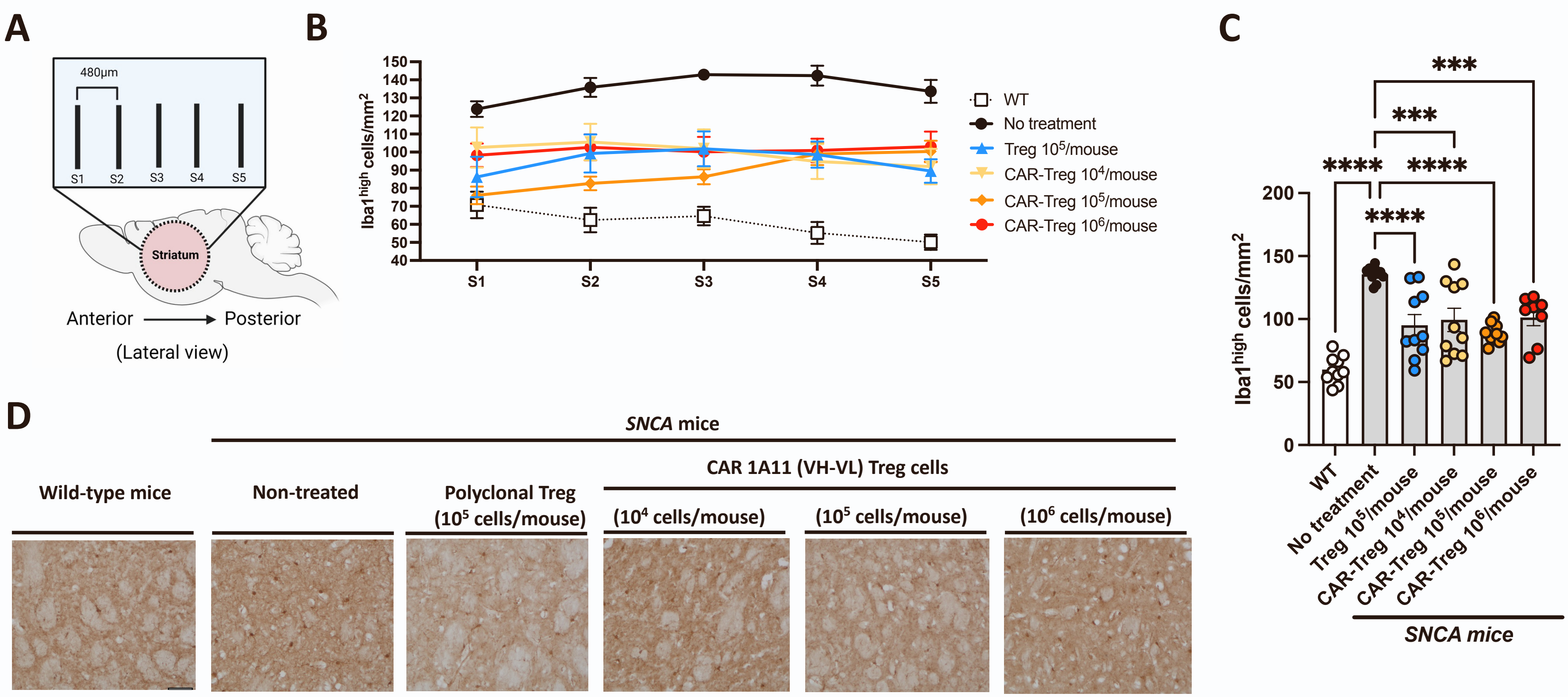

Figure S15. A single injection of CAR-Treg therapy reduces neuroinflammation in *SNCA* mice. Related to Figure 3. Ten weeks old *SNCA* mice were treated with a single i.v. injection of CAR-Treg expressing the CAR 1A11 in the VH-VL conformation (10^4 cells/mouse, beige; 10^5 cells/mouse, orange; 10^6 cells/mouse, red), or polyclonal Treg (10^5 cells/mouse, blue). Non-treated *SNCA* mice (black) or wild-type mice (WT, white) were used as controls. The extent of microgliosis was evaluated at 20 weeks of age by IHC analysis of Iba1 along the striatal antero-posterior axis. (**A**) Scheme illustrating the analysis of five different sections (S1-S5), separated by 480 µm, along the striatum. (B, C) Quantifying of the number of cells displaying high immunoreactivity for Iba1 per area in every single section (**B**) or the average from all the sections together (**C**). Each symbol represents an individual mouse (n = 8-10 mice per group). Mean ± SEM are shown. ***, p<0.001; ****, p<0.0001 by one-way ANOVA followed by Tukey's *post-hoc* test. (**D**) Representative images from section 3 are shown for each experimental group. Bar scale, 100 µm.

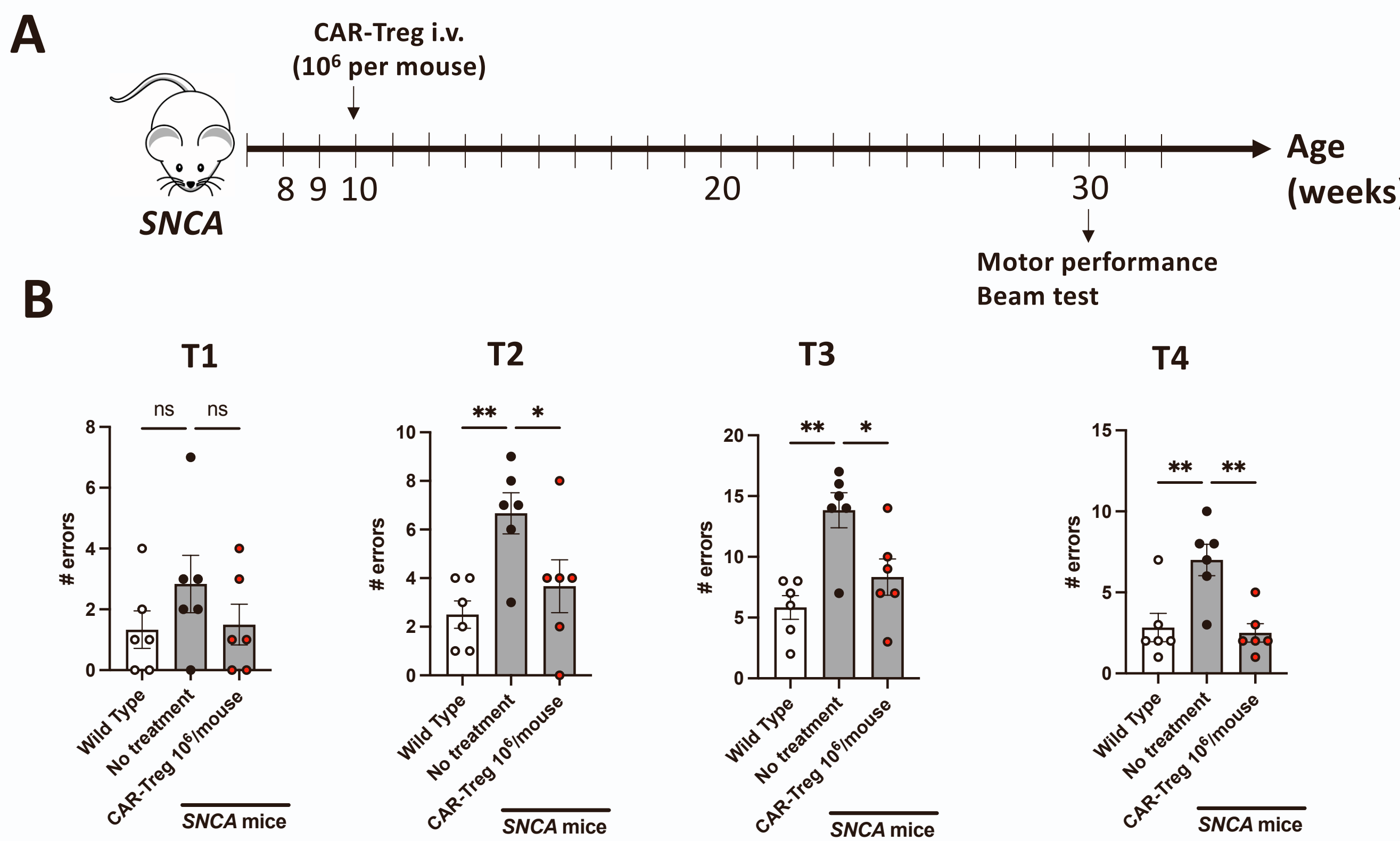

Figure S16. A single injection of the CAR-Treg therapy improves the motor decline in *SNCA* mice even 20 weeks after the treatment: Detailed beam test analysis. Related to Figure 3. Ten weeks old *SNCA* mice were treated with a single i.v. injection of CAR-Treg expressing the CAR1A11 in the VH-VL conformation (10^6 cells/mouse, red symbols). Non-treated *SNCA* mice (black symbols) were used as controls. Age matched wild-type mice (white symbols) were also evaluated as controls. The motor performance was evaluated at 30 weeks of age by the beam test. (**A**) Illustration of the experimental strategy. (**B**) The number of errors was quantified in each single tract (T1-T4). Each symbol represents an individual mouse (n = 6 mice per group). Mean ± SEM are shown. *, p<0.05; **, p<0.01 compared to the control *SNCA* mice without treatment (black symbols) by one-way ANOVA followed by Dunnett's *post-hoc* test. ns, non-significant.

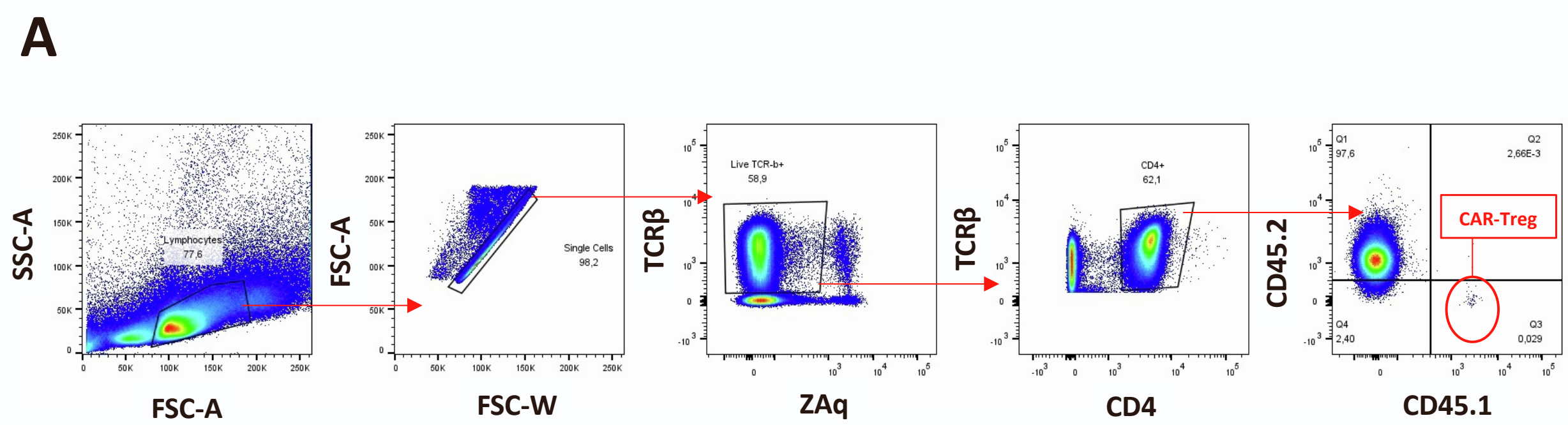

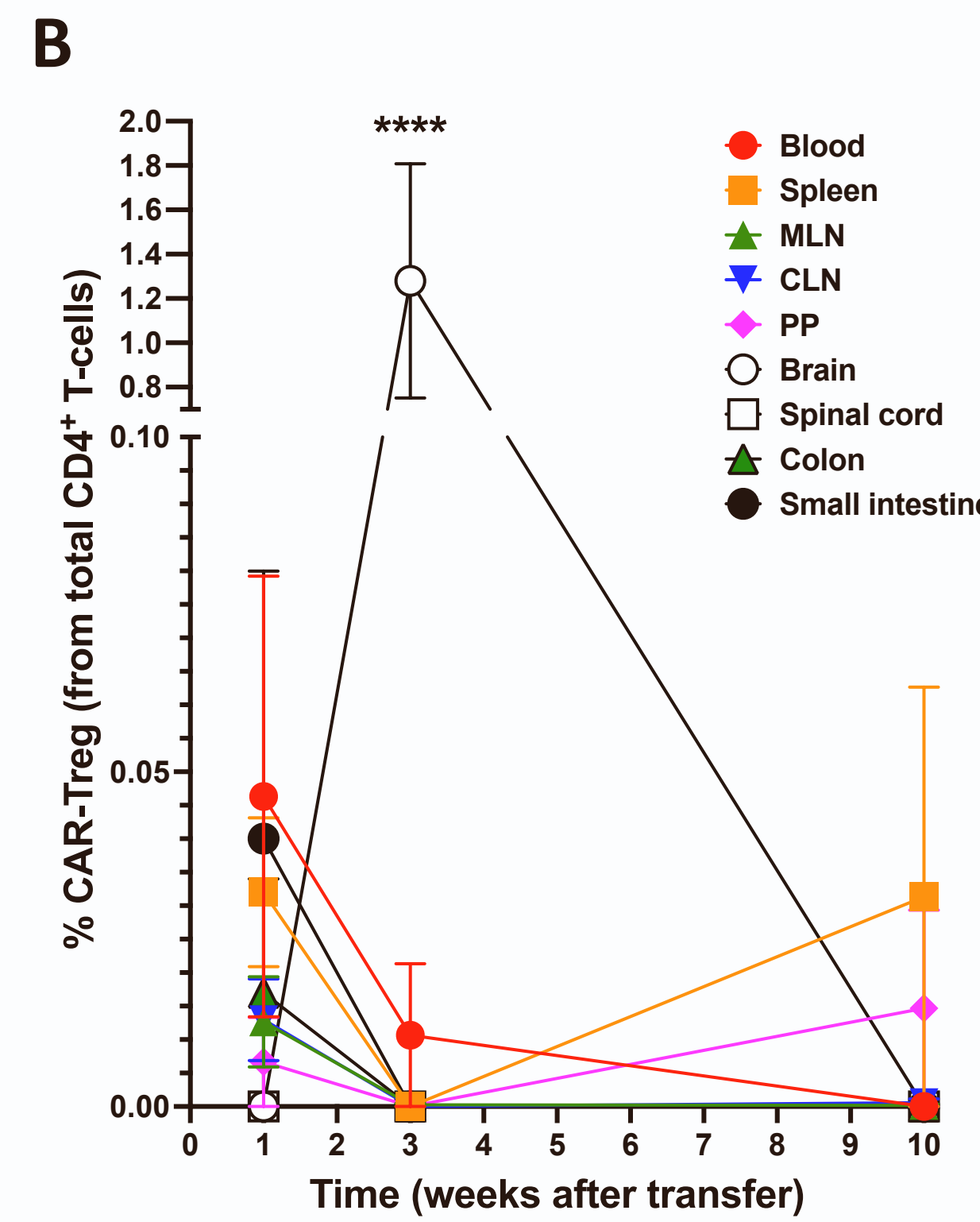

Figure S17. Spatiotemporal analysis of the distribution of CAR-Treg cells in *SNCA* mice. Related to Figure 3. Ten-week-old *SNCA* mice (CD45.2$^{+/+}$) received a single injection of congenic CAR-Treg cells (10^6 cells/mouse; CD45.1$^{+/+}$). One, three, or ten weeks later, mice were sacrificed and the presence of CAR-Treg cells was tracked by flow cytometry in different tissues, including blood, spleen, mesenteric lymph nodes (MLN), cervical lymph nodes (CLN), Peyer's patches (PP), brain, spinal cord, colonic mucosa, and small-intestinal mucosa. (**A**) Gating strategy used to analyse transferred cells (CD45.1$^+$) within the live (ZAq$^-$) CD4$^+$ T-cell population of each tissue. (**B**) Quantification of the relative percentage of donor cells (CD45.1$^+$) within the total CD4$^+$ T-cell population analysed in each tissue. Data represent mean ± SEM of three mice per group. ****, $p<0.0001$ compared to the percentage of CAR-Treg in the blood as determined by two-way ANOVA followed by Dunnett's post-hoc test.

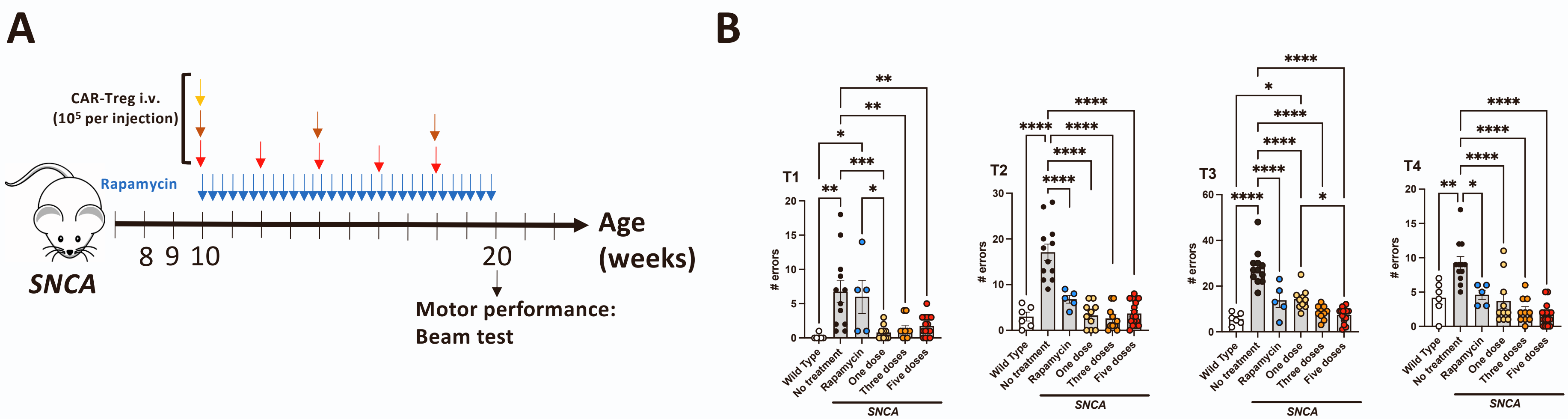

Figure S18. Evaluation of the efficacy of CAR-Treg therapy administered at different number of injections in *SNCA* mice at the level of motor performance. **Detailed beam test analysis. Related to Figure 3.** Ten weeks old *SNCA* mice (grey bars) were treated with a single injection (at week 10; beige symbols), three injections (at weeks 10, 14 and 18; orange symbols), or five injections (at weeks 10, 12, 14, 16, 18; red symbols) of i.v. CAR-Treg expressing the CAR1A11 in the VH-VL conformation (10^5 cells/mouse). *SNCA* mice treated with rapamycin (7,5 mg/Kg i.p. three times a week for 10 weeks, blue symbols) or non-treated (black symbols) were used as controls. Age matched wild-type mice (white bars, white symbols) were also evaluated as controls. At the week 20 of age, the motor performance was quantified by the beam test. (**A**) Illustration of the experimental strategy. (**B**) The number of errors in each single tract (T1-T4) quantified in the beam test. Each symbol represents an individual mouse (n = 5-16 mice per group). Mean ± SEM are shown. *, p<0.05; **, p<0.01; ***, p<0.001; ****, p<0.0001 by one-way ANOVA followed by Tukey's *post-hoc* test.

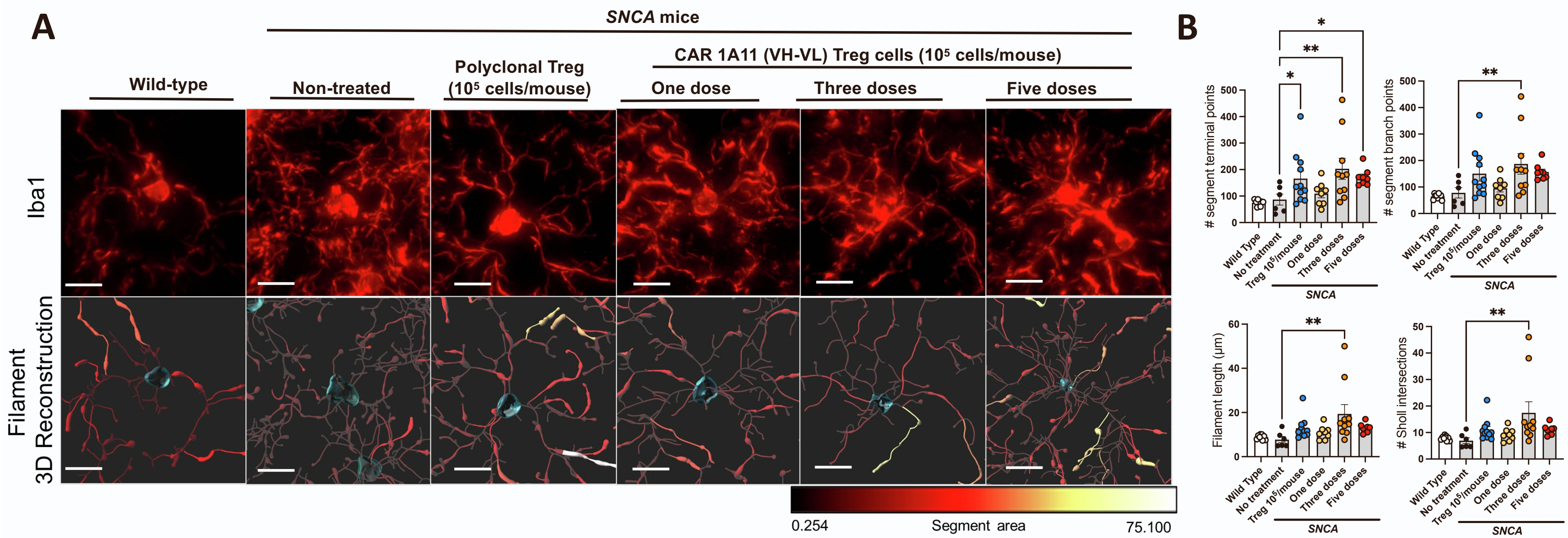

Figure S19. Morphometric analysis of microglia in *SNCA* mice treated with CAR-Treg therapy. Related to Figure 4. Ten weeks old *SNCA* mice (grey bars) were treated with a single injection (at week 10; yellow symbols), three injections (at weeks 10, 14 and 18; orange symbols), or five injections (at weeks 10, 12, 14, 16, 18; red symbols) of i.v. CAR-Treg expressing the CAR1A11 in the VH-VL conformation (10^5 cells/mouse). *SNCA* mice treated with a single injection of polyclonal Treg (10^5 cells/mouse, blue symbols) or non-treated (black symbols) were used as controls. Age matched wild-type mice (white bars, white symbols) were also evaluated as controls. At the week 20 of age, striatal microglia was identified by Iba1 fluorescent immunostaining and morphometric analysis was conducted. (**A**) Representative maximum intensity projections from confocal images showcasing microglial morphology are shown in top panels. Representative three-dimensional reconstructions (filament tool) of microglia from the different groups using Imaris are shown in bottom panels using color codes based on statistics of the filament area are shown in bottom panels. Scale bar: 10 µm. (**B**) Several morphometric quantifications are shown, including number of segment terminal points, number of segment branch points, filament length and number of sholl intersections. Each symbol represents an individual slice (four slices per mouse; n = 3-5 mice per group). Mean ± SEM are shown. *, $p<0.05$; **, $p<0.01$ compared to the control (non-treated *SNCA* mice; black symbols) by one-way ANOVA.

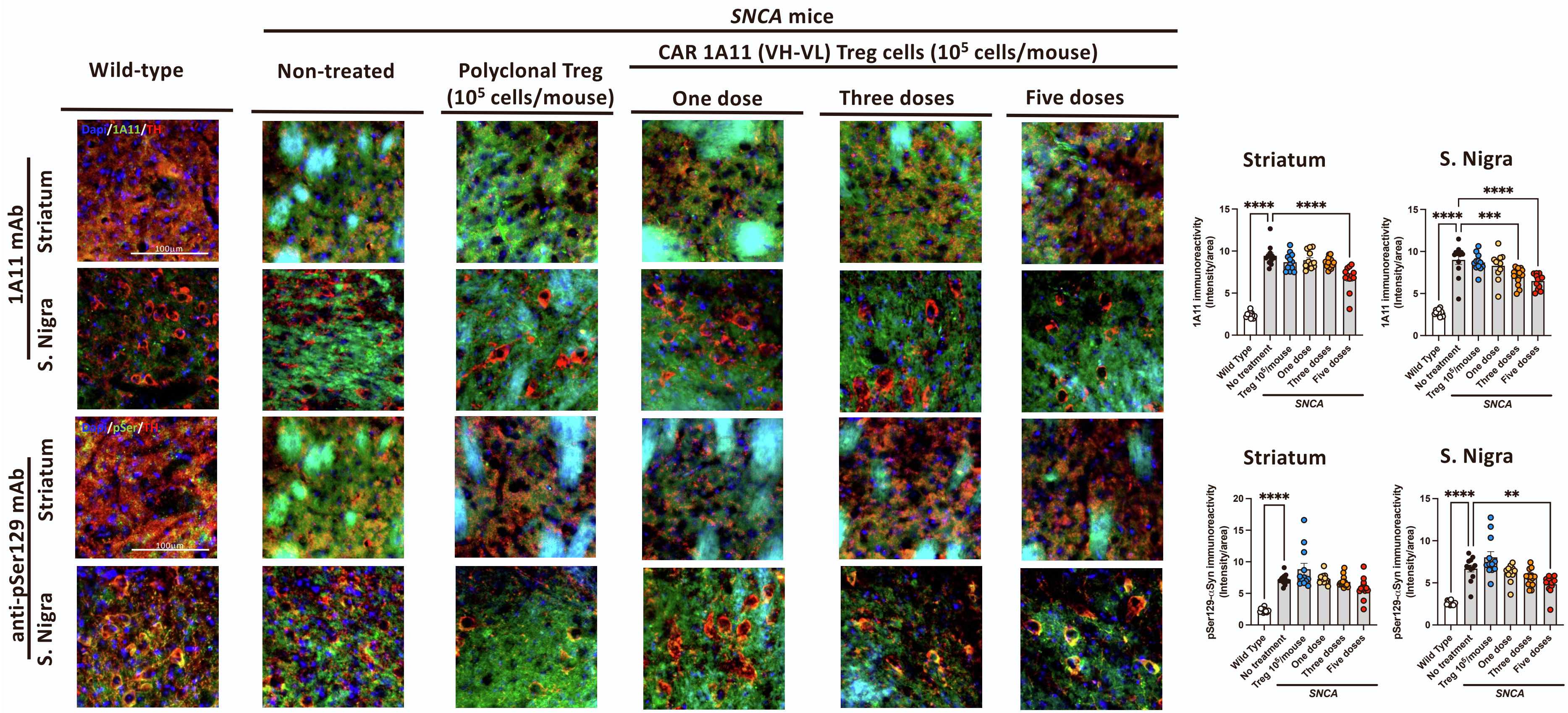

Figure S20. Synuclein pathology is reduced in the nigrostriatal pathway of *SNCA* mice treated with multiple injections of CAR-Treg therapy. Related to Figures 3 and 4. Ten weeks old *SNCA* mice (grey bars) were treated with a single injection (at week 10; yellow symbols), three injections (at weeks 10, 14 and 18; orange symbols), or five injections (at weeks 10, 12, 14, 16, 18; red symbols) of i.v. CAR-Treg expressing the CAR1A11 in the VH-VL conformation (10^5 cells/mouse). *SNCA* mice treated with a single injection of polyclonal Treg (10^5 cells/mouse, blue symbols) or non-treated (black symbols) were used as controls. Age matched wild-type mice (white bars, white symbols) were also evaluated as controls. At the week 20 of age, the immunoreactivity of the mAb 1A11 and pSer129-**α**Syn were analysed in the SN and striatum by immunofluorescence. TH and nuclei were also immunostained. Representative images are shown in left panels. Bar scale, 100 µm. The fluorescence intensity associated to mAb 1A11 and pSer129-**α**Syn normalised per area was quantified in right panels. Each symbol represents an individual slice (three slices per mouse; n = 5-6 mice per group). Mean ± SEM are shown. **, p<0.01; ***, p<0.001; ****, p<0.0001 compared to the control (non-treated *SNCA* mice; black symbols) by one-way ANOVA followed by Dunnett's *post-hoc* test.

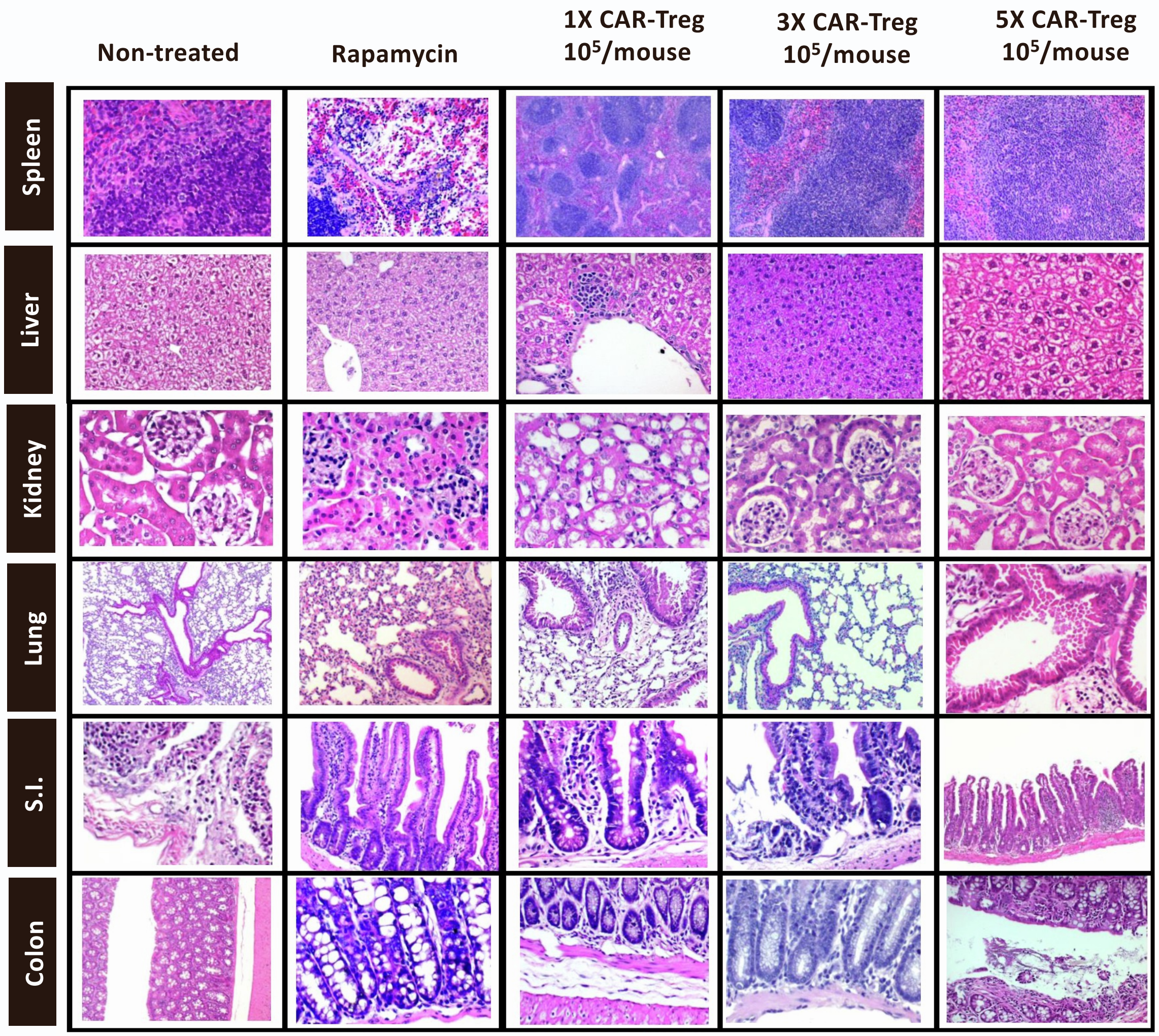

Figure S21. Histopathological analysis of different organs from *SNCA* mice treated with CAR-Treg therapy. **Related to Figures 3 and 4.** *SNCA* mice were treated as shown in figure 3A. At 20 weeks of age, mice were sacrificed and histological analysis was performed on the liver, spleen, kidney, lung, small intestine (S.I.), and colon. Representative images are shown. The group of mice treated with rapamycin was the only one displaying histological anomalies in most organs analysed.

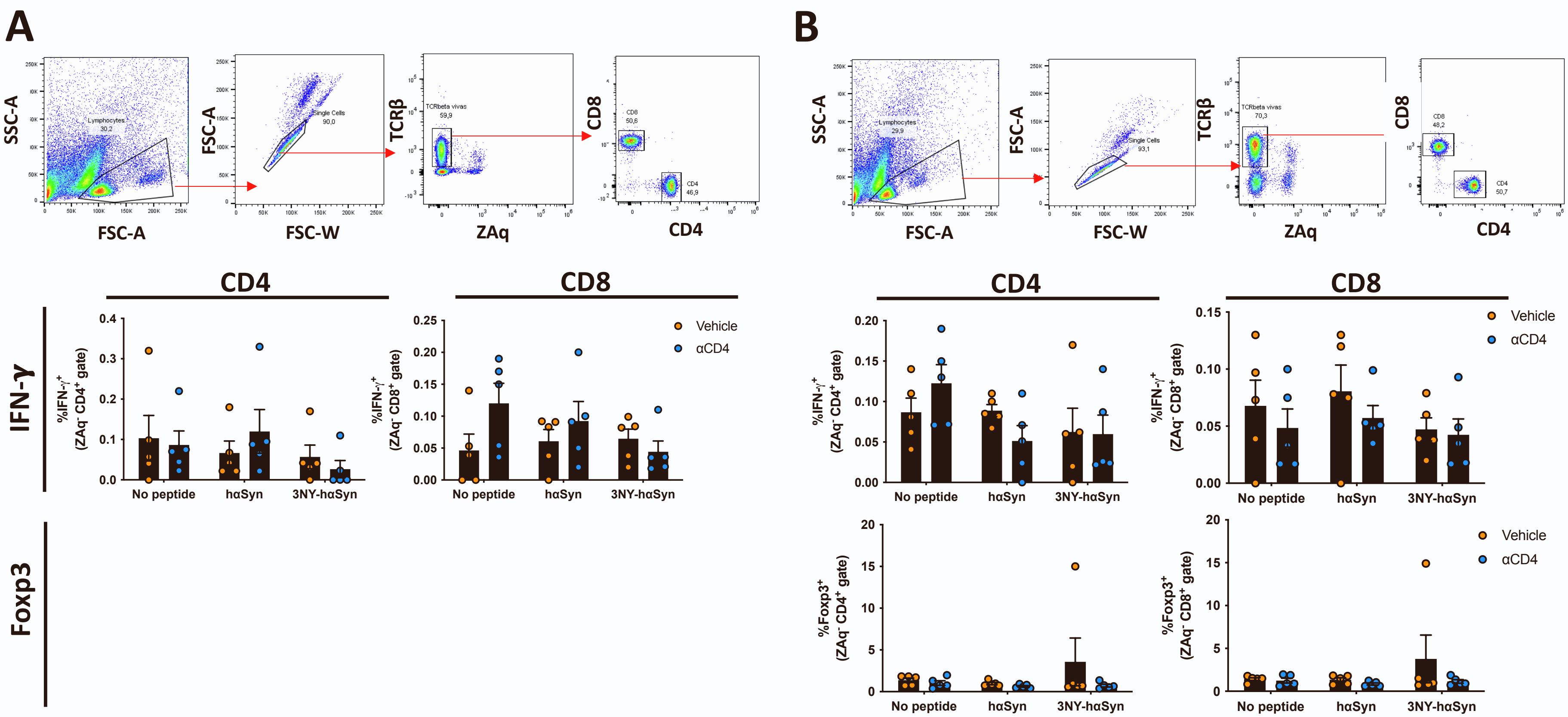

Figure S22. Analysing the T-cell response to αSyn-derived antigens in cervical and mesenteric lymph nodes upon transiently depleting CD4⁺ T-cells in *SNCA* mice treated with CAR-Treg. Related to Figure 5. Eight weeks old *SNCA* mice received three injections of a depleting anti-CD4 mAb (i.p., 100 µg/injection; blue symbols) or vehicle (orange symbols) separated for 24 h between injections. At week 10 of age, mice were treated with i.v. CAR-Treg (10^5 cells/mouse). At the week 20 of age, mononuclear cells were isolated from the cervical (**A**) mesenteric (**B**) lymph nodes. Cells were stimulated with peptides derived from unmodified hαSyn, 3NYhαSyn, or without peptides, and 24 h later the extent of IFN**γ** and Foxp3 expression in the CD4+ and CD8+ T-cell populations was analysed by intracellular immunostaining followed by flow cytometry. Values are the percentage of IFN**γ**+ or Foxp3+ cells in the $CD4^+$ $TCR\beta^+$ ZAq^- and $CD8^+$ $TCR\beta^+$ ZAq^- population. Top panels show the gating strategy and bottom panels show the quantification. Each symbol represents an individual mouse (n = 5 mice per group). Mean ± SEM are shown. No significant differences were found using the multiple t-test.

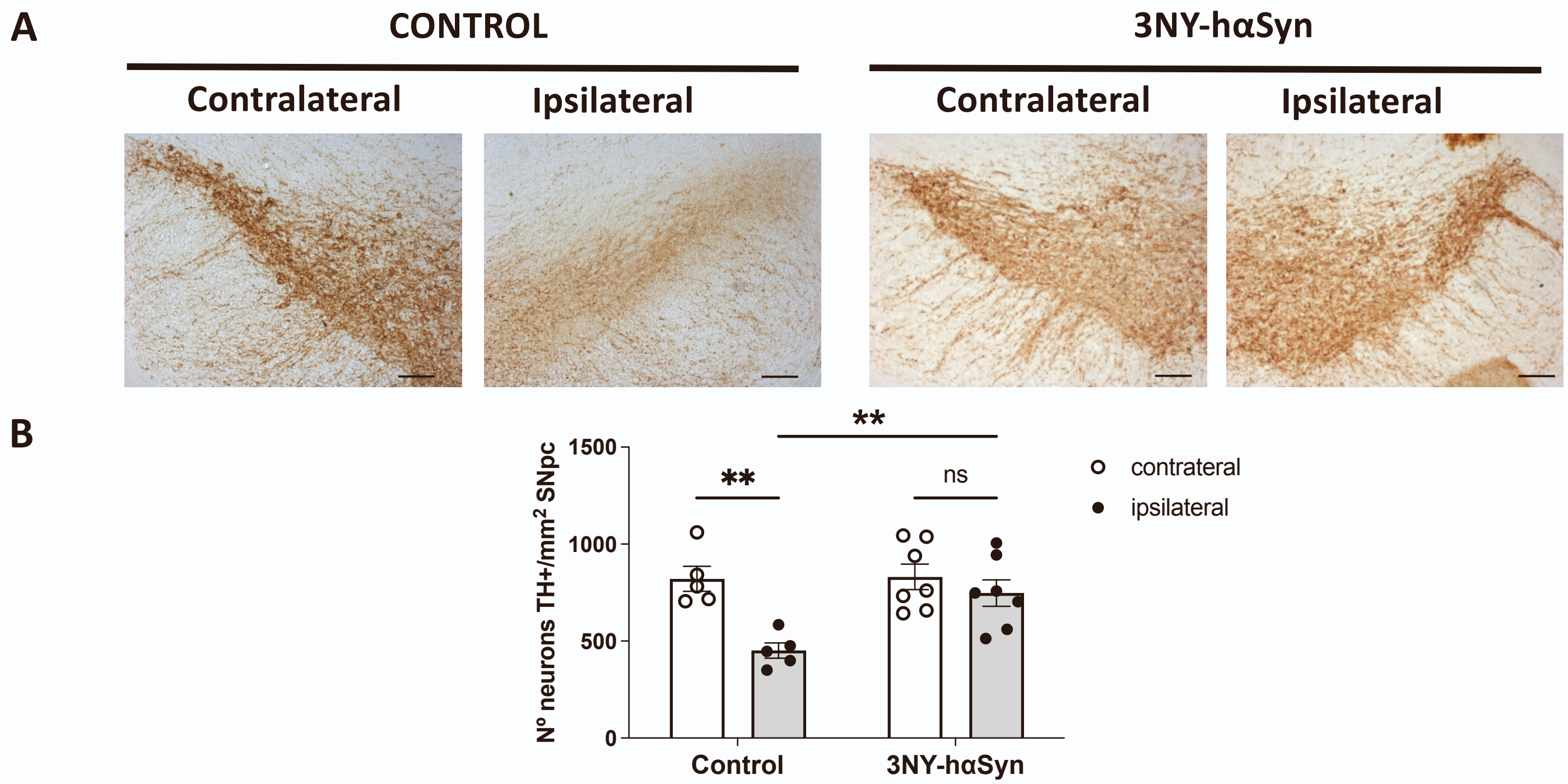

Figure S23. Vaccination with 3NYαSyn exerts a therapeutic effect attenuating neurodegeneration in the AAV-hαSyn mouse model. Related to Figure 6. C57BL/6 mice received unilateral stereotaxic injections of AAV-hαSyn (10^{10} vg/mice) in the right SN (ipsilateral). Three weeks later, mice were s.c. Immunised with complete Freund's adjuvant (CFA) containing 10 µg 3NY-hαSyn peptide (3NY-hαSyn group) or with CFA alone (Control group). Five-weeks after the first immunisation, mice in the 3NY-hαSyn group received a second s.c. immunisation with 10 µg 3NY-hαSyn peptide in incomplete Freund's adjuvant (IFA), while the control group received a s.c. injection of IFA alone. Seven-weeks after the second immunisation, mice were euthanised and the extent of dopaminergic neuron loss was determined by immunohistochemical (IHC) analysis of TH in the SN. (**A**) Representative images obtained for IHC analyses of TH are shown. Left SN were analysed as internal controls (contralateral). Scale bars, 80 **µ**m. (**B**) Quantification of TH+ neurons per area in the SNpc. **, $p<0.01$ by two-ways ANOVA followed by the Sidak's multiple comparison test; n = 5-7 mice per group. ns, not significant.

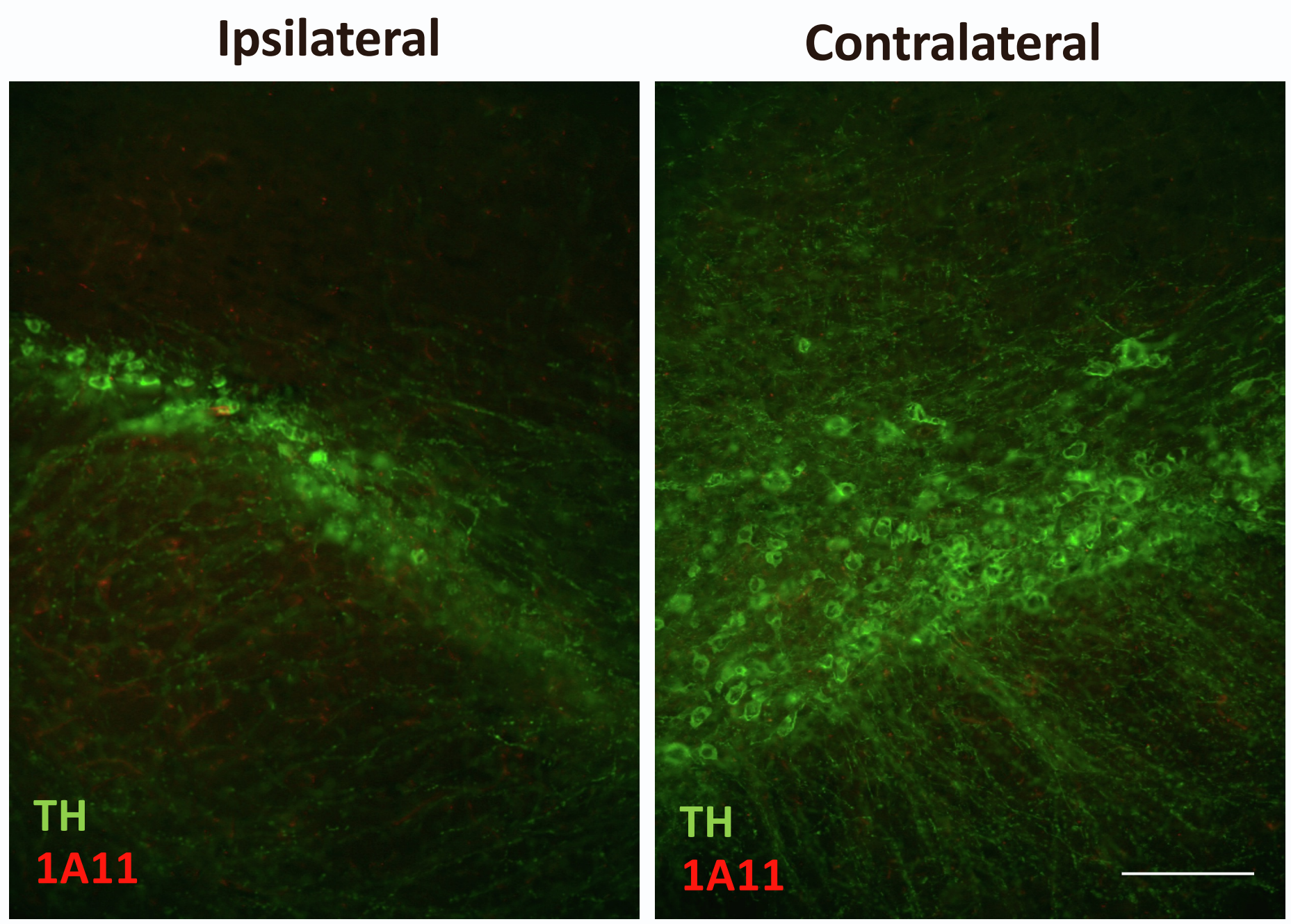

Figure S24. Immunoreactivity of 1A11 mAb in the SN of mice treated with AAV-hαSyn. Related to Figure 6. Wild-type C57BL/6 mice were anesthetized and received the stereotaxic inoculation of AAV encoding for human α-synuclein under the control of the chicken beta-actin (CBA) promoter (10^{10} vg/mouse) into the right substantia nigra (SN). After 15 weeks, mice were sacrificed and incubated with 1A11 mAb (1A11) followed by AlexaFluor546-coupled anti-mouse secondary antibody in brain slices containing the area of the SN. TH was also immunostained with a rabbit anti-TH pAb followed by an AlexaFluor488-coupled anti-rabbit secondary antibody. TH and 1A11 associated fluorescence was analysed by epifluorescence microscopy. Representative images of merge staining of TH (green) and 1A11 (red) are shown. Bar, 100 μm.

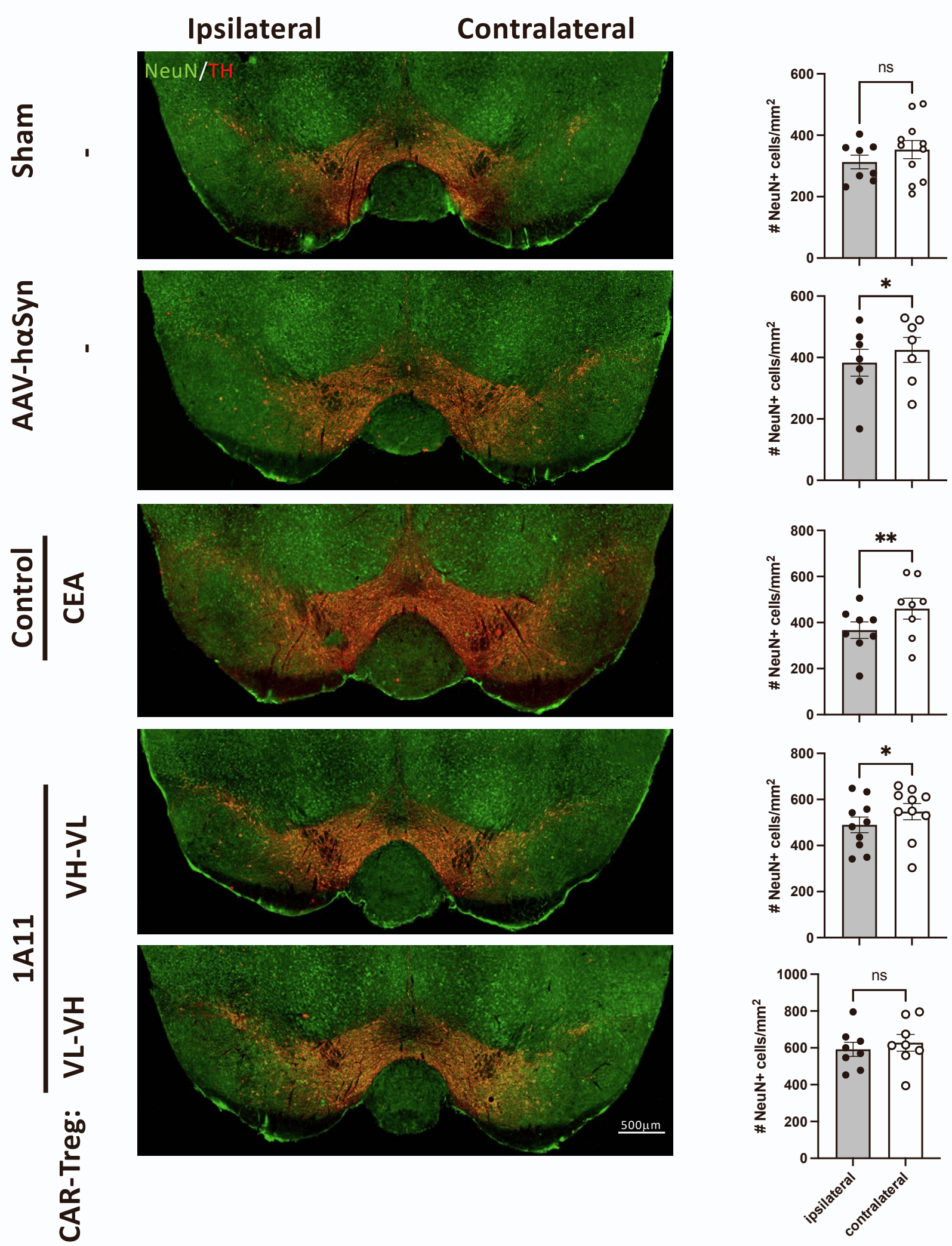

Figure S25. The reduction of the number of TH+ cells in the SN of mice treated with AAV-hαSyn and receiving CAR-1A11 T-cell therapy correlates with decreased number of NeuN+ neurons. Related to Figure 6. Wild-type C57BL/6 mice received the stereotaxic inoculation of AAV encoding human **α**-Synuclein (AAV-h**α**Syn; 10^{10} vg/mouse) or vehicle (sham) into the right substantia nigra (SN). Three weeks after the surgery, mice received the i.v. injection of CAR-Treg ($3x10^4$ cells per mouse) expressing the CAR1A11 in the VH-VL or VL-VH conformations. As a control group, some mice received the i.v. injection of CAR-Treg ($3x10^4$ cells per mouse) expressing the CAR-CEA (control CAR). Sixteen weeks after the stereotaxic surgery, nigral sections were co-immunostained for TH (red) and the pan-neuronal marker NeuN (green), and the number of NeuN+ cells were quantified in the SN (TH+ area). Left panels show representative images. Bar, 500 **µ**m. Right panels show the quantification of NeuN+ cells in the TH+ areas in the ipsilateral (grey bars) and the contralateral (white bars) SN. Data represent the mean with SEM from 2 slices per mouse from 4-5 mice per group. *, p<0.05; **, p<0.01 by paired Student's t-test. ns, non-significant.

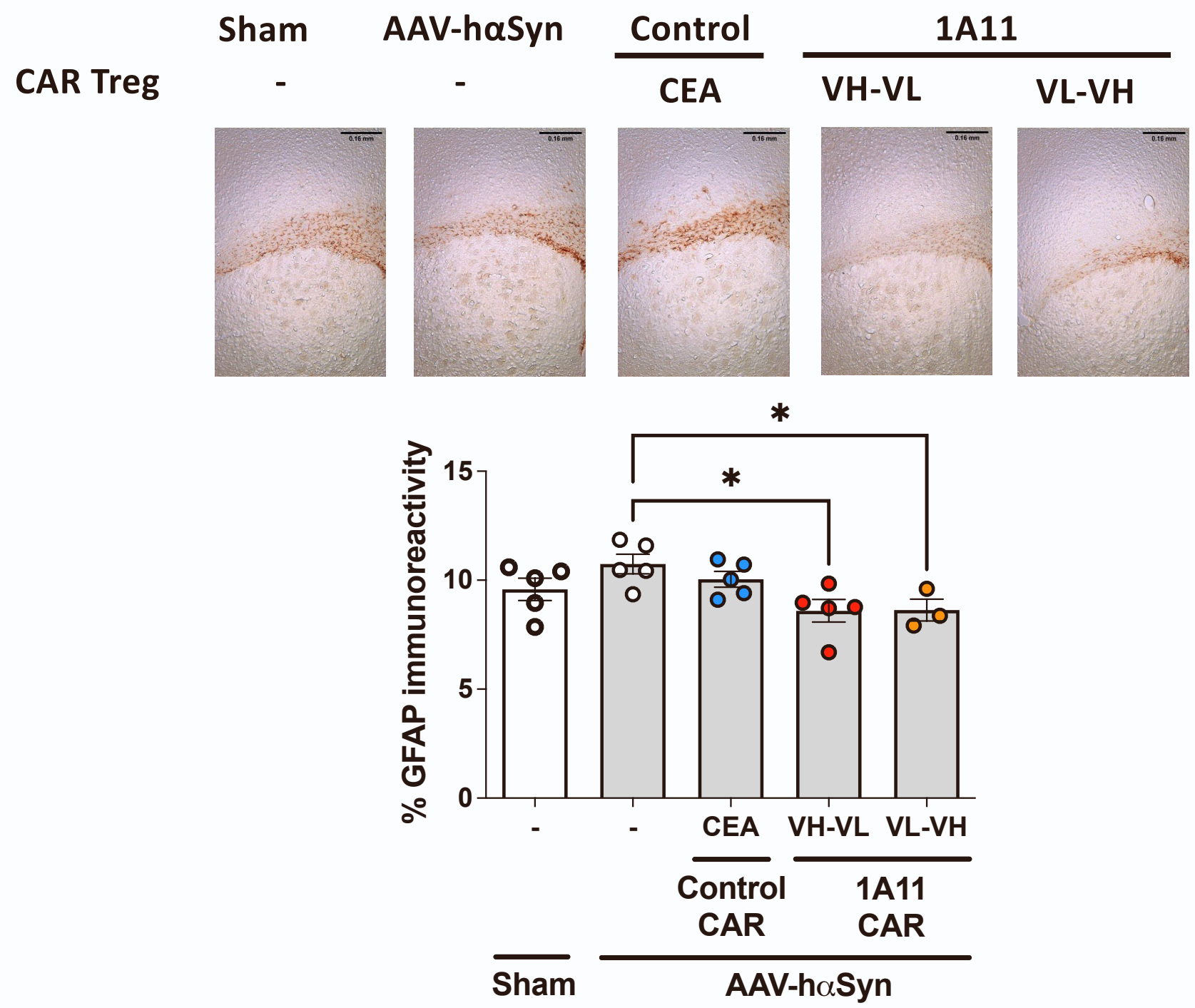

Figure S26. Analysis of astrogliosis in the striatum of mice treated with AAV-hαSyn and receiving CAR-1A11 T-cell therapy. Related to Figure 6. Wild-type C57BL/6 mice received the stereotaxic inoculation of AAV encoding human α-Synuclein (AAV-hαSyn; 10^{10} vg/mouse; grey bars) or vehicle (sham; white bar) into the right substantia nigra (SN). Three weeks after the surgery, mice received the i.v. injection of CAR-Treg ($3x10^4$ cells per mouse) expressing the CAR1A11 in the VH-VL (red symbols) or VL-VH (orange symbols) conformations. As a control group, some mice received the i.v. injection of CAR-Treg ($3x10^4$ cells per mouse) expressing the CAR-CEA (control CAR; blue symbols). Sixteen weeks after the stereotaxic surgery, mice were sacrificed and astrogliosis was evaluated by immunohistochemical analysis of GFAP in the striatum. Top panels show representative images for each experimental group. Scale bars, 160 μm. Bottom panel show the quantification of the density of GFAP immunoreactivity. Data represent the mean with SEM from 5 mice per group. *, $p<0.05$ by one-way ANOVA followed by Dunnett's *post-hoc* test.

Table S1. Peptides used in the study. Related to figure 1 A and B.

Name	Sequence	Provider
hαSyn111-140	GILEDMPVDPDNEAYEMPSEEGYQDYEPEA	Genscript
3NY-hαSyn	GILEDMPVDPDNEAY*EMPSEEGY*QDY*EPEA	Genscript
NY125-hαSyn	GILEDMPVDPDNEAY*EMPSEEGYQDYEPEA	Genscript
NY133-hαSyn	GILEDMPVDPDNEAYEMPSEEGY*QDYEPEA	Genscript
NY136-hαSyn	GILEDMPVDPDNEAYEMPSEEGYQDY*EPEA	Genscript
NY125,133-hαSyn	GILEDMPVDPDNEAY*EMPSEEGY*QDYEPEA	Genscript
NY125,136-hαSyn	GILEDMPVDPDNEAY*EMPSEEGYQDY*EPEA	Genscript
NY133,136-hαSyn	GILEDMPVDPDNEAYEMPSEEGY*QDY*EPEA	Genscript
3NY-scrambled	LQSEVEY*GAEAEDMGEPIPMPY*DNY*CDPEED	Genscript
3NY-scrambled	LQSEVEY*GAEAEDMGEPIPMPY*DNY*CDPEED	Biosynthesis
5FAM-hαSyn	#GILEDMPVDPDNEAYEMPSEEGYQDYEPEA	Genscript
5FAM-NY133,136-hαSyn	#GILEDMPVDPDNEAYEMPSEEGY*QDY*EPEA	Genscript
5FAM-3NY-hαSyn	#GILEDMPVDPDNEAY*EMPSEEGY*QDY*EPEA	Genscript

Y*, Indicates nitrated tyrosine; #, indicates 5FAM coupled in the N-terminus.

Table S2. Demographic features and disease activity of Parkinson's disease patients. Related to figure 1 D and E.

Gender (Female/Male)	11 (3/8)
Age	69,4 ± 12,3
MoCA test[1]	26,2 ± 4,3
UPDRS[2]	40,2 ± 24,9
Hoehn and Yahr[3]	2,1 ± 1,2
Schwab & England[4]	77 ± 23

[1] Montreal Cognitive Assessment (scale 0-30); [2] Unified Parkinson's Disease Rating Scale (scale 0-199); [3] Modified Hoehn and Yahr scale (scale 0-5); [4] Schwab & England activities of daily living scale (%).
