# Supplementary material for: Preclinical evaluation of nitrated α-synuclein-specific CAR-Treg therapy in Parkinson’s disease
Source: Cell Rep Med. 2026 Aug 12;7(9):102983. doi: 10.1016/j.xcrm.2026.102983 (PMC13589471; doi:10.1016/j.xcrm.2026.102983)
Supplement: Supplementary file 2 [file mmc2.pdf]

Cell Reports
Medicine

Article

Preclinical evaluation of nitrated α-synuclein-specific CAR-Treg therapy in Parkinson's disease

Graphical abstract

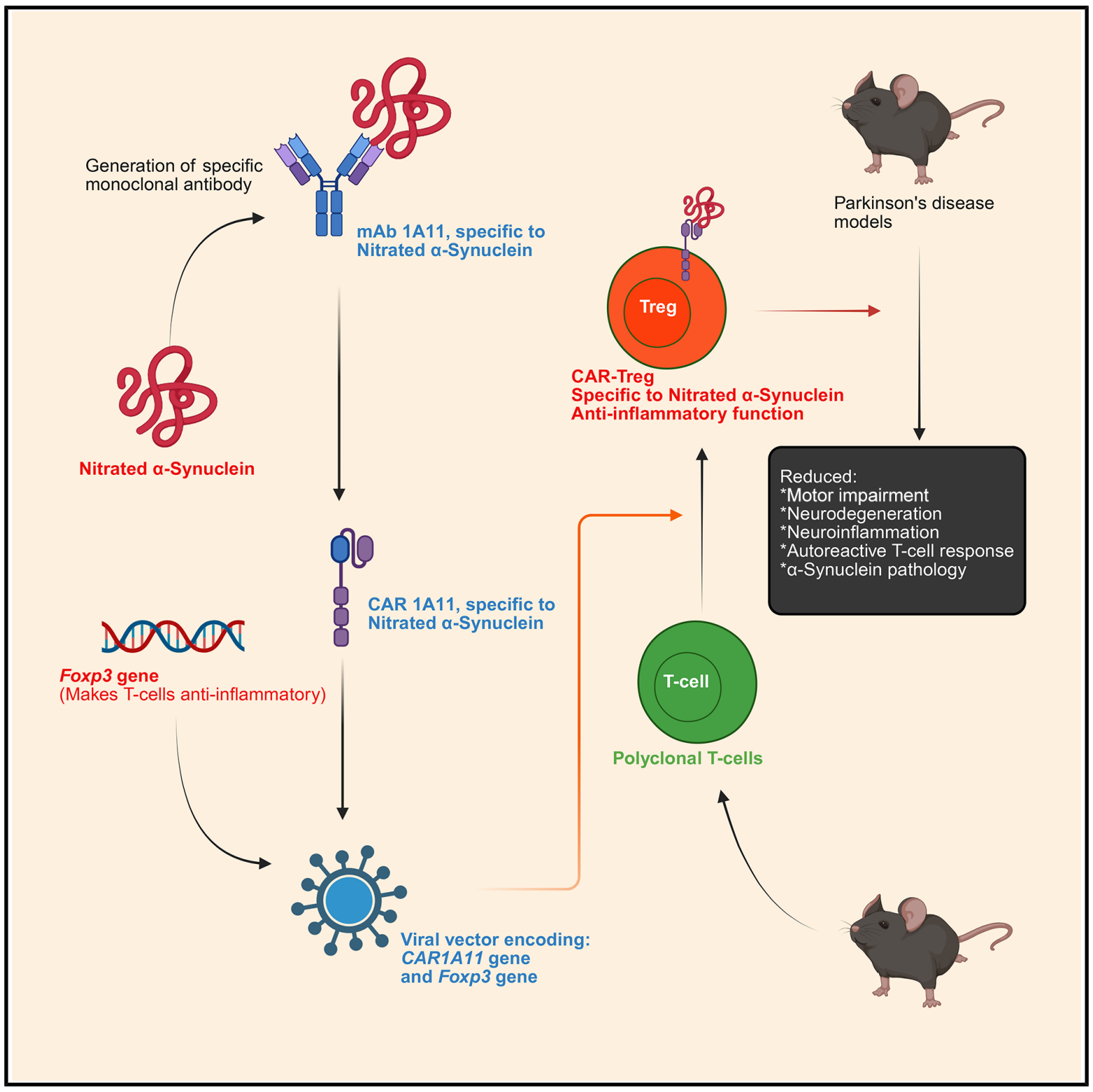

Highlights

- An anti-nitrated α-synuclein antibody detects higher signal in PD serum
- Serum anti-nitrated α-synuclein signal correlates with PD stage
- Nitrated α-synuclein-specific CAR-Treg improve motor function
- CAR-Treg reduce brain pathology and striatal Iba1-high myeloid-cell abundance

Authors

Valentina Ugalde, Daniela Elgueta, Sandra Espinoza, ..., Sergio A. Quezada, Roque Villagra, Rodrigo Pacheco

Correspondence

rpacheco@cienciavida.org

In brief

Ugalde et al. develop a regulatory CAR-T cell therapy that targets nitrated α-synuclein, a disease-associated form of α-synuclein. In two Parkinson's disease mouse models, this therapy reduces harmful α-synuclein-specific T cell responses, limits brain pathology, and improves motor function.

Ugalde et al., 2026, Cell Reports Medicine 7, 102983
September 15, 2026 © 2026 The Author(s). Published by Elsevier Inc.
https://doi.org/10.1016/j.xcrm.2026.102983

CellPress

Cell Reports Medicine

CellPress OPEN ACCESS

Article

Preclinical evaluation of nitrated α-synuclein-specific CAR-Treg therapy in Parkinson's disease

Valentina Ugalde,[1,2] Daniela Elgueta,[1,14] Sandra Espinoza,[1,14] Ornella Chovar-Vera,[1,3] Ernesto López,[4] Alejandra Catenaccio,[5] Francisca Álvarez-Astudillo,[6] Dafne Franz,[4] Vanesa Sanchez-Guajardo,[7] María Inés Becker,[8,9] Diego Figueroa,[10] Zulmary Manjarres,[11] Sofía Hidalgo,[2,10] Álvaro Lladser,[3,10] Cheril Tapia-Rojas,[5,12] Alejandra Loyola,[6,12] Sergio A. Quezada,[4] Roque Villagra,[13] and Rodrigo Pacheco[1,3,15,*]

[1]Laboratory of Neuroimmunology, Centro Científico y Tecnológico de Excelencia Ciencia & Vida, Fundación Ciencia & Vida, Santiago, Chile
[2]Fundación Arturo López Pérez OECI Cancer Center, Santiago, Chile
[3]Facultad de Medicina, Universidad San Sebastián, Santiago, Chile
[4]Cancer Immunology Unit, University College London (UCL) Cancer Institute, London, UK
[5]Laboratory of Neurobiology of Aging, Centro Científico y Tecnológico de Excelencia Ciencia & Vida, Fundación Ciencia & Vida, Santiago, Chile
[6]Laboratory of Epigenetics and Chromatin, Centro Científico y Tecnológico de Excelencia Ciencia & Vida, Fundación Ciencia & Vida, Santiago, Chile
[7]BRIGHT, Technical University of Denmark, 2800 Kongens Lyngby, Denmark
[8]Fundación Ciencia y Tecnología para El Desarrollo (FUCITED), Santiago, Chile
[9]Departamento de Investigación y Desarrollo Biosonda Corporation, Santiago, Chile
[10]Laboratory of Immunoncology, Centro Científico y Tecnológico de Excelencia Ciencia & Vida, Fundación Ciencia & Vida, Santiago, Chile
[11]Department of Neuroscience, Center for Advanced Pain Studies, University of Texas at Dallas, Richardson, TX 75080, USA
[12]Facultad de Ciencias, Universidad San Sebastián, Santiago, Chile
[13]Departamento de Ciencias Neurológicas Oriente, Facultad de Medicina, Universidad de Chile, 7500691 Santiago, Chile
[14]These authors contributed equally
[15]Lead contact
*Correspondence: rpacheco@cienciavida.org
https://doi.org/10.1016/j.xcrm.2026.102983

SUMMARY

Current evidence indicates that Parkinson's disease (PD) involves T cell-mediated inflammation, which plays a fundamental role in promoting neuroinflammation and neurodegeneration in patients and animal models. These T cells are specific to α-synuclein-derived antigens, including nitrated α-synuclein (NαSyn). Here, we sought to develop an experimental immunotherapy for PD based on the generation of regulatory T cells (Treg) specific to NαSyn, using the chimeric antigen receptor (CAR) technology. Accordingly, we first obtained an antibody specific to human α-synuclein containing three nitrated tyrosine residues (3NY-hαSyn), which displayed specific immunoreactivity in the serum of PD patients which correlated with the clinical score. Afterward, we generated CAR-Treg specific to 3NY-hαSyn and tested them in two PD models involving human α-synuclein. The CAR-Treg therapy substantially inhibited the inflammatory T cell response specific to α-synuclein-derived antigens, neuroinflammation, neurodegeneration, and the motor decline. This preclinical study indicates that the CAR-Treg therapy represents a promising therapeutic strategy for treating PD patients.

INTRODUCTION

Parkinson's disease (PD) is a neurodegenerative disorder characterized by the loss of dopaminergic neurons of the nigrostriatal pathway and the deposition of Lewy bodies in the brain, which are protein inclusions composed mainly of α-synuclein (αSyn) containing pathogenic posttranslational modifications. Importantly, Lewy bodies have been shown to stimulate Toll-like receptors in microglial cells, favoring the acquisition of M1 phenotype in these cells, leading to chronic neuroinflammation, a fundamental process required for the development of neurodegeneration in PD.[1] However, depending on the cytokine milieu and other T cell-derived molecular cues, microglial cells might also acquire the anti-inflammatory M2 functional phenotype, dampening neuroinflammation.[2] Thus, microglia constitute the central player in the control of neuroinflammation, and T cells represent a major regulator of microglial activation.[3]

Evidence in human and animal models has shown the generation of nitrated forms of αSyn (NαSyn) in the substantia nigra (SN) of individuals with PD,[4–6] which are found mainly in the

© 2026 The Author(s). Published by Elsevier Inc.
This is an open access article under the CC BY license (http://creativecommons.org/licenses/by/4.0/).

αSyn inclusions contained in Lewy bodies. Of note, the nitration of αSyn, which is a consequence of oxidative stress, might lead to the generation of neo-antigens.[7] Furthermore, studies in mice and recently in humans, have shown that oxidized αSyn constitutes a major antigen for the T cell-mediated immune response involved in PD.[5,8–10] In this regard, it has been shown that NαSyn might be captured and presented by antigen-presenting cells (APCs) in the deep cervical[5] and mesenteric (unpublished data) lymph nodes to naive T cells with specificity for these neo-antigens. Once activated, $CD4^+$ T cells acquire inflammatory phenotypes, such as T helper-1 (Th1) and Th17, they infiltrate the SN where microglial cells act as local APCs presenting NαSyn on major histocompatibility complex (MHC) class II, thus re-stimulating T cells.[5,11–13] Restimulated $CD4^+$ T cells produce high local levels of IFN-γ and TNF-α, thus promoting further inflammatory activation of microglial cells (M1 microglia).[14–16] Activated M1 microglia produces high levels of glutamate, TNF-α, and reactive oxygen and nitrogen species, which, in turn, induce neuronal death and further generation of oxidized and nitrated proteins, including NαSyn.[1] Thus, this mechanism constitutes a vicious cycle, which results in chronic neuroinflammation and represents the engine of the progression of neurodegeneration (Figure S1). Moreover, $CD8^+$ T cells specific to αSyn-derived antigens, including NαSyn, might be activated by APCs in PD, where they might directly kill neurons presenting their cognate antigen on MHC class I.[7,10,17] Accordingly, several studies have shown that T cell deficiency leads to a significant protection of neurodegeneration in mouse models of PD,[5,15,18–20] thereby indicating that T cell-driven inflammation plays a critical role in the development of neurodegeneration of the nigrostriatal pathway. Importantly, the natural attenuation of inflammatory $CD4^+$ and $CD8^+$ T cell responses is exerted by regulatory $CD4^+$ T cells (Treg), which inhibit the inflammatory function of conventional T cells with specificity for the same antigen. Of note, Treg-mediated responses have been shown to promote M2 phenotype in microglial cells, dampening the development of neuroinflammation in PD animal models.[7] Moreover, Treg cells have been shown to limit the extent of astrogliosis induced by ischemic brain injury, reducing neuroinflammation and potentiating neurological recovery in mice.[21]

Here, we hypothesized that treating PD individuals with NαSyn-specific Treg attenuates neuroinflammation and neurodegeneration of the nigrostriatal pathway (Figure S1). Thus, we aimed to generate an experimental therapy for PD based on inhibiting the inflammatory T cell response to nitrated forms of αSyn, which seem to constitute leading antigens driving the chronic inflammation in this disorder. To this end, we generated an immunosuppressive Treg-mediated response specifically directed to a short highly nitrated segment of the human αSyn (hαSyn), which was tested in two PD mouse models: (1) transgenic mice overexpressing hαSyn that develop the disease spontaneously with age (the *SNCA* mouse model), and (2) the PD mouse model induced by the stereotaxic delivery of adeno-associated viral vector (AAV) encoding the overexpression of hαSyn in the SN (the AAV-hαSyn model). For this purpose, we used retroviral transduction of polyclonal T cells with a chimeric antigen receptor (CAR) and Foxp3 (the master transcription factor controlling the Treg suppressive phenotype) to generate NαSyn-specific Treg cells as a therapeutic approach for PD. It is noteworthy that CAR T therapy has been proven to be safe in humans and approved for the use in cancer patients by the Food and Drug Administration (FDA) of the United States (i.e., Yescara and Kymriah).

RESULTS

Generation and characterization of monoclonal antibodies specific to 3NY-hαSyn

As NαSyn accumulates in pathogenic inclusions in PD and other synucleinopathies,[4] we aimed to develop a Treg-mediated immunosuppressive response specific to NαSyn by using CAR technology, which enables MHC-independent T cell activation through antibody-derived antigen-recognition domains linked to T cell signaling modules.[22,23] To generate the CAR extracellular domain, we developed monoclonal antibodies (mAbs) against the C-terminal $h\alpha Syn_{111-140}$ peptide containing three nitrated tyrosine residues (3NY-hαSyn; Table S1). BALB/c mice were immunized with 3NY-hαSyn (Figure S2), inducing serum immunoreactivity to 3NY-hαSyn but not to unmodified αSyn (Figures S3 and S4). Hybridoma screening identified two mAbs selectively recognizing 3NY-hαSyn: 1A11 (IgG1κ) and 8E7 (IgAκ) (Figures S5A and S5B). Cross-reactivity analyses using a nitrated scrambled peptide showed that 1A11, unlike 8E7, recognized 3NY-hαSyn but not 3NY-scrambled (Table S1) or unmodified hαSyn (Figure S6). Thus, 1A11 was selected for CAR generation, and its variable domains of the light (VL) and heavy (VH) chain sequences were obtained to produce a recombinant IgG2a 1A11 mAb.

To further analyze the specificity of the epitope recognized by the 1A11 mAb, we evaluated the binding of the antibody to a set of peptides derived from the $h\alpha Syn_{111-140}$ containing only one nitrated tyrosine residue (Y125, Y133, or Y136), two nitrated tyrosine residues, or all three nitrated tyrosine residues (Table S1). The results show a significant binding of the 1A11 mAb only to the peptides NY133,136-hαSyn and 3NYhαSyn, indicating that nitrations on Y133 and Y136, but not on Y125, are essential for antigen recognition by the 1A11 mAb (Figure 1A). In addition, we determined the affinity of 1A11 mAb binding to the peptides NY133,136-hαSyn, 3NYhαSyn, and NY133-hαSyn (as a control). The results revealed high affinity for both peptides, being in the nanomolar (nM) range for 3NYhαSyn (Kd = 13.38 nM) and in the picomolar (pM) range for NY133,136-hαSyn (Kd = 393 pM) (Figure 1B). Altogether, these results indicated that the 1A11 mAb recognizes an epitope of the $h\alpha Syn_{111-140}$ containing nitrated Y133 and Y136 with high affinity. To analyze whether the epitope recognized by the 1A11 mAb is accessible in the native conformation of pathogenic hαSyn deposits, we tested the immunoreactivity of this mAb in *SNCA* mice. As previous studies have shown that *SNCA* mice develop αSyn pathology in the intestine,[24] we tested the immunoreactivity of the recombinant 1A11 mAb in the colonic mucosa of these mice. The results showed high 1A11 mAb immunoreactivity in the colonic tissue of *SNCA* mice compared with age-matched wild-type (WT) littermates (Figure 1C). Thus, these analyses suggest that the epitope recognized by the 1A11 mAb is accessible on the surface of hαSyn inclusions in parkinsonian mice. Further characterization

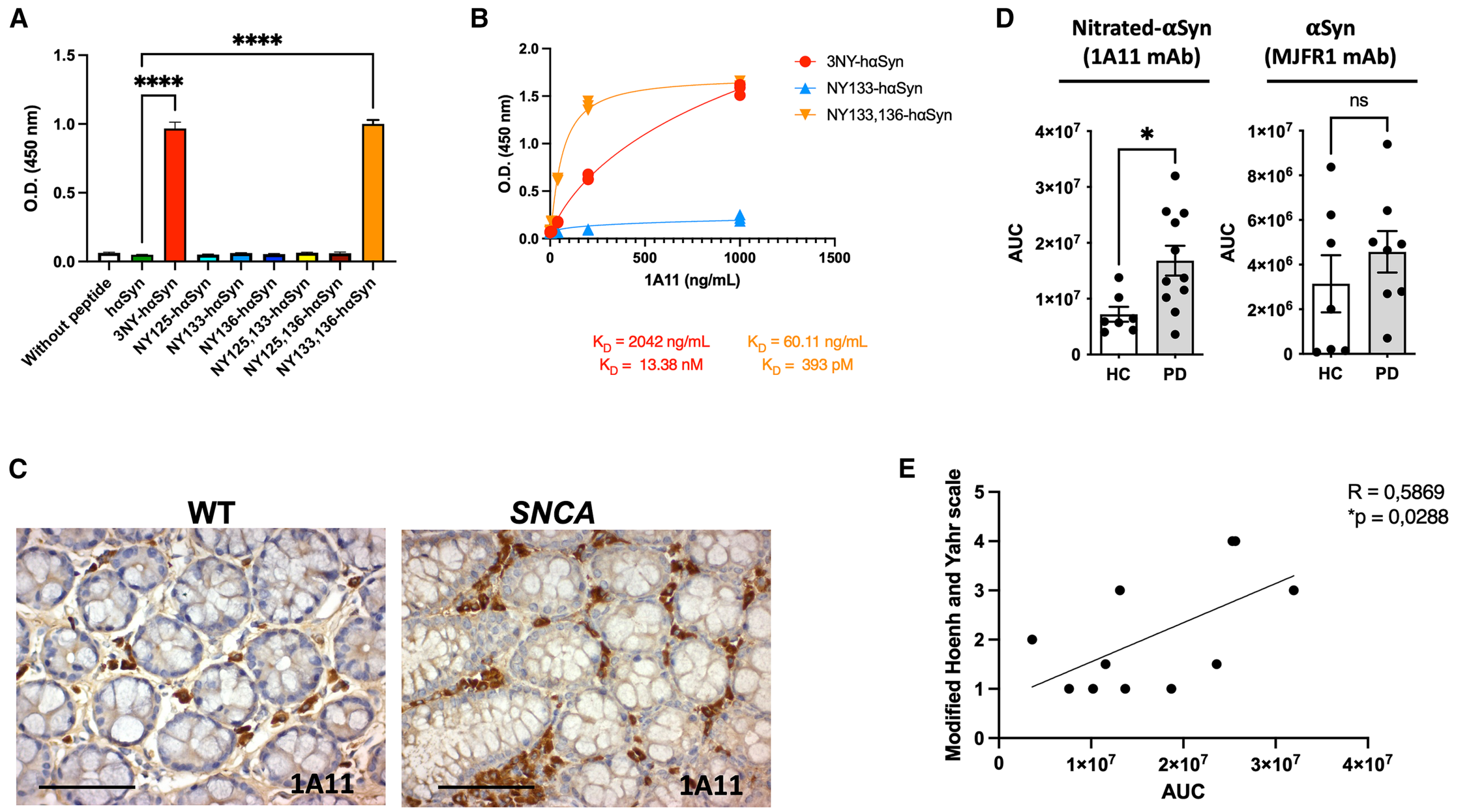

Figure 1. Characterizing the immunoreactivity of the 1A11 mAb

(A) Evaluating 1A11 mAb specificity. ELISA plates were activated with peptides (10 μg/mL) derived from the hαSyn$_{111–140}$ sequence, including the unmodified (hαSyn, green), containing all three tyrosine residues Y125, Y133, and Y136, and nitrated (3NY-hαSyn, red bar), containing only one nitrated tyrosine (blue tone bars) or two nitrated tyrosine residues (yellow-orange tone bars). Afterward, the plates were incubated with recombinant 1A11 mAb (1 μg/mL).

(B) Evaluating 1A11 mAb affinity. The NY133-hαSyn (blue), NY133,136-hαSyn (orange), or 3NY-hαSyn (red) peptides (10 μg/mL) were incubated in plates, and then the recombinant 1A11 mAb was added at different concentrations. In (A) and (B), detection of immunoreactivity was conducted by ELISA using a secondary anti-mouse Ig (H + L) antibody conjugated to horseradish peroxidase (HRP) followed by the addition of 3,3’5,5’-tetramethylbenzidine (TMB). The optic density was quantified at 450 nm. In (A), values are mean ± SD from triplicates, and in (B), all values are shown (triplicates). Non-linear regression was done to calculate the Kd for each respective peptide (following the color code), and the value is expressed as nM or ng/mL. Kd for NY133-hαSyn was not calculable.

(C) Immunoreactivity of 1A11 mAb in the distal colon of 13-week-old *SNCA* mice and WT littermates. Representative images of immunohistochemical analysis of 1A11 mAb (0.5 μg/mL) immunoreactivity. Scale bars, 100 μm.

(D) Quantification of the immunoreactivity of nitrated (1A11 mAb; left) or total (MJFR1 mAb; right) αSyn in serum from PD patients and HCs.

(E) 1A11 mAb immunoreactiviy in the serum of PD patients versus clinical score. R value is indicated. In (D), mean ± SEM values are shown. In (D) and (E), area under the curve (AUC) values were quantified by densitometry of dot blots. Each symbol represents data from an individual subject. *, $p < 0.05$; ****, $p < 0.0001$, by (A) one-way ANOVA followed by Tukey’s post-hoc test, (D) Student’s t test, and (E) Pearson’s correlation test. ns, non-significant.

of the 1A11 mAb by western blot analysis revealed that its immunoreactivity, similarly to that of the anti-pSer129-hαSyn mAb, was associated with aggregated hαSyn species (≈30, 60, and 180 kDa), whereas monomeric hαSyn was not detected by this antibody in the brain and colonic extracts obtained from *SNCA* mice (Figures S7A and S7B). Conversely, the 1A11 mAb was able to detect the monomeric form of hαSyn (≈17 kDa) in serum samples obtained from patients with PD (Figure S7C). To assess the relevance of the epitope recognized by the 1A11 mAb in humans, we conducted an exploratory evaluation of the immunoreactivity of this antibody in the serum of a small group of PD patients (see Table S2) and compared it with heathy controls (HCs). These exploratory results showed significantly higher immunoreactivity of the 1A11 mAb in the serum from PD patients than in that from HCs (Figure 1D). Conversely, the immunoreactivity of an antibody detecting total monomeric αSyn (MJFR1 mAb) was comparable in serum samples from PD patients and HCs (Figure 1D). Moreover, the extent of 1A11 mAb immunoreactivity in the serum of PD patients significantly correlated with the clinical score determined by the modified Hoehn and Yahr scale (Figure 1E). Although it did not reach a significant p value with the small number of individuals tested, the extent of 1A11 mAb immunoreactivity in the serum of PD patients was nearly significantly correlated with other common scales used to determine PD severity, including the Schwab and England score, UPDRS, and Montreal cognitive assessement (MoCA) (Figures S8A–S8C) but was not correlated with the age (Figure S8D). Altogether, these results encouraged us to choose the 1A11 hybridoma for further generation of CARs specific to 3NY-hαSyn.

Generation of Treg expressing a functional CAR specific to 3NY-hαSyn

To transform polyclonal conventional T cells in Treg with specificity to 3NY-hαSyn, we attempted to generate viral vectors encoding Foxp3, the master transcription factor controlling

the Treg phenotype,[25] and a CAR specific to 3NY-hαSyn. Accordingly, we analyzed the sequences encoding the antigen recognition from the VH and VL chains of the 1A11 hybridoma. To generate a single-chain fragment variable (scFv) in the extracellular domain of the CAR-1A11, we assembled VH and VL sequences separated by a spacer sequence in two different conformations: 5′-VH-spacer-VL-3' (VH-VL) and 5′-VL-spacer-VH-3' (VL-VH). The scFv region was linked to the transmembrane region of CD4 by an extracellular hinge region from IgG1. Signaling motifs of CD3z and CD28 responsible of triggering T cell activation and co-stimulation were included in the intracellular region of the CAR-1A11. *Foxp3* was inserted at 3′ of the CAR-1A11 construct, separated by a sequence encoding the peptide 2A.[26] Both genes were inserted in a pBullet vector at the 3′ side of the cytomegalovirus (CMV) promoter (see the design of the CAR-1A11 viral vector in Figure S9). To generate cell lines stably producing CAR-1A11/Foxp3 retroviral particles in the supernatant, ECO Phoenix™ retroviral packaging cells (which express endogenous gag, pol, and envelope proteins) were transfected with the viral vector, and the IgG1$^+$ cells were isolated by cell sorting, as described before.[27] CAR-1A11/Foxp3 retroviral particles contained in the fresh supernatant of IgG1$^+$ ECO Phoenix™ retroviral packaging cells were used to transduce total splenic CD3$^+$CD4$^+$ T cells, obtaining approximately 14% transduction efficiency (Figure S10). IgG1$^+$CD4$^+$ T cells were isolated by cell sorting and used for further experiments. To determine whether the CAR1A11 was able to bind to the epitope recognized by the 1A11 mAb, we studied the binding of NY133,136-hαSyn and 3NY-hαSyn peptides to the cells expressing CAR1A11. The results showed selective binding of CAR1A11, with high affinity to both NY133,136-hαSyn (Kd = 386 nM) and 3NY-hαSyn (Kd = 43 nM), but not to hαSyn (Kd > 10 μM) (Figure 2A). To evaluate the functionality and specificity of CAR-Treg bearing the CAR1A11, the extent of T cell activation and IL-10 production was determined *in vitro* when incubated with the cognate antigen contained in NY133,136-hαSyn and 3NY-hαSyn peptides, a polyclonal stimulus independent of the CAR1A11 (anti-CD3/anti-CD28), and irrelevant antigens, including the unmodified-hαSyn peptide and the carcinoembrionary antigen (CEA). The results showed a significant increase in the activation marker CD69 on the cell surface and in the production of IL-10 when CAR-Treg cells were stimulated by anti-CD3/anti-CD28 or by the cognate antigen (Figures 2B and 2C). The same antigens also upregulated the surface expression of CD25, PD1, and GITR, but not CTLA4 (Figure S11). *In vitro* suppression assays confirmed the ability of CAR-Treg cells to reduce the activation of T cells obtained from *SNCA* mice and revealed that this effect depends, at least in part, on IL-10, as the blockade of IL-10 reduced their suppressive potency (Figures 2D and 2E). To explore the translational potential of CAR-Treg therapy in human cells, human peripheral blood CD4$^+$ T cells were transduced with lentiviral vectors encoding the CAR1A11 construct generated using fully human components, including the hinge and transmembrane regions, signaling domains, and the human *FOXP3* gene (Figures S12A and S12B). Stimulation of these human CAR-Treg cells (hCAR-Treg) with the cognate antigen induced T cell activation and IL-10 production (Figures S12C and S12D), supporting the translational potential of this technology for human application. Altogether these results suggested that CAR-Treg cells bearing the CAR1A11 recognize the same antigen as the 1A11 mAb and that antigen binding triggers T cell activation and the Treg function.

CAR-Treg therapy specific to 3NY-hαSyn attenuates the motor decline and neuroinflammation in the *SNCA* mouse model

To determine the therapeutic potential of these CAR-Treg cells in a preclinical model of PD, we chose the transgenic *SNCA* mice, which involve a mild overexpression of hαSyn (≈4-folds the levels of endogenous αSyn), starting after birth and developing the disease spontaneously with age.[28] Of note, this mouse model develops the disease in a manner dependent on the gut microbiota[29] and displays αSyn pathology in the colonic mucosa (unpublished data) as early as 13 weeks of age (Figure 1C), which agrees with the gut-brain axis associated with most PD patients.[30] Indeed, NαSyn was detected at low levels in the colon of *SNCA* mice as early as 10 weeks of age (pre-symptomatic) and subsequently increased with age (Figure S13). Interestingly, a sharp increase in NαSyn and monomeric αSyn levels was observed in both the colon and brain at 15 weeks of age (Figure S13), coinciding with the onset of motor decline in this mouse model (unpublished data). We recently observed that the *SNCA* mice develop T cell-driven inflammation in response to hαSyn-derived antigens in the mesenteric lymph nodes (MLNs) (unpublished data). To test whether 3NY-hαSyn-specific CAR-Treg cells were able to limit the inflammatory T cell response to hαSyn-derived antigens *in vivo*, a single injection of CAR-Treg (10^5 cells per mouse) was administered to the pre-symptomatic *SNCA* mice, and 10 weeks after, the inflammatory T cell response to hαSyn-derived antigens associated with the colonic mucosa was analyzed in the MLNs. The results showed a substantial inhibition of the inflammatory T cell response to 3NY-hαSyn and unmodified hαSyn in those *SNCA* mice receiving the CAR-Treg therapy (Figure 2F). These results suggest that the *in vivo* recognition of NαSyn by CAR-Treg efficiently dampens the inflammatory T cell response to hαSyn-derived antigens. Accordingly, we next tested the therapeutic potential of CAR-Treg cells at the level of motor decline when administered at different doses—10^4, 10^5, and 10^6 cells per mouse—starting at a pre-symptomatic age (10 weeks of age), and analyzing the effects at a symptomatic age (20 weeks of age) (Figure 3A). As a negative control of motor impairment, we used age-matched WT littermates, and as a control of ani-inflammatory therapy, we included a group of *SNCA* mice treated with polyclonal Treg, which has been shown to reduce neuroinflammation and neurodegeneration in PD animal models.[31] Notably, all doses tested of CAR-Treg exerted significant reduction in the motor impairment when the total number of errors was quantified (Figures 3A and 3B; Figure S14). Nevertheless, when single tracts were analyzed separately, 10^4 CAR-Treg/mouse was better in tract 1 and 10^6 CAR-Treg/mouse was better in tract 4 (Figure S14). To evaluate whether the therapeutic effect of CAR-Treg therapy observed in *SNCA* mice at the level of motor performance was associated with a reduction in neuroinflammation, we next determined the extent of microgliosis by immunohistochemical analysis of Iba1 through the anteroposterior axis of the striatum (Figure S15A). The results showed that all

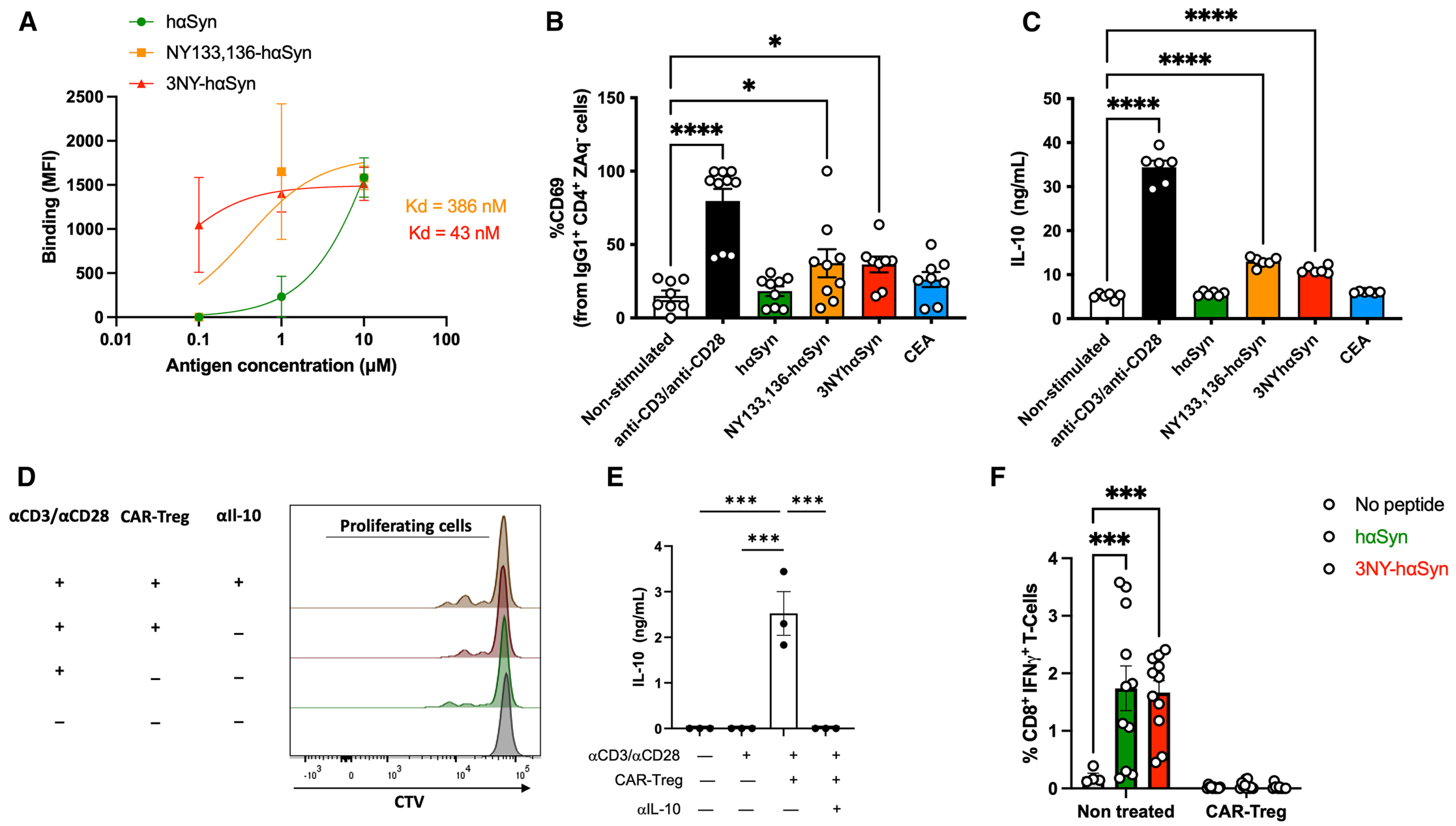

Figure 2. Confirming the functionality of the CAR1A11 *in vitro* and *in vivo*

(A) NIH cells were transduced with CAR1A11 (VH-VL conformation) and then incubated with increasing concentrations of the 5-FAM-coupled peptides derived from the C terminal of the hαSyn (hαSyn$_{111–140}$) without modifications (green), containing two nitrated tyrosine residues (NY133,136-hαSyn; orange), or containing three nitrated tyrosine residues (3NY-hαSyn; red) for 10 min. After washing the cells, the fluorescence associated with 5-FAM was quantified in the population of live cells expressing CAR1A11 (IgG1$^+$ ZAq$^-$) by flow cytometry. Values are the mean fluorescence intensity (MFI). Data represent the mean ± SEM from triplicates. Kd values were determined by non-linear regression and are shown in the color associated with the corresponding peptide. Kd for the hαSyn peptide was not calculable.

(B and C) CAR-Treg response to antigen recognition. 96-well plates were incubated with hαSyn$_{111–140}$-derived peptides (at 1 μg/mL), including unmodified hαSyn (green), NY133,136-hαSyn (orange), and 3NY-hαSyn (red). As controls, the plates were incubated with only vehicle (white), carcinoembrionary antigen (CEA;1 μg/mL, blue), or anti-CD3 (1 μg/mL) plus anti-CD28 (2 μg/mL) (black). Afterward, CAR-Treg cells (transduced with the CAR1A11 in the VH-VL conformation; 5×10^4 cells/well) were incubated with the different antigens for 24 h, and the surface CD69 expression was determined in live CD4$^+$ T cells expressing CAR1A11 (IgG1$^+$ ZAq$^-$ population) by flow cytometry (B), while IL-10 production was assessed in the culture supernatant by ELISA (C). Data from three and two independent experiments conducted in triplicates are shown in (B) and (C), respectively. Mean ± SEM values are shown.

(D and E) CAR1A11 (VH-VL conformation) was activated and then co-cultured with CellTrace Violet (CTV)-loaded CD25$^-$ TCRβ$^+$ T cells isolated from 20-week-old *SNCA* mice (ratio, 3:100) and incubated with anti-CD3 (1 μg/mL) plus anti-CD28 (2 μg/mL) and 133,136-NYhαSyn in the presence or absence of a neutralizing anti-IL-10 mAb for 72 h.

(D) Representative histograms showing the extent of proliferating T cells, determined by the dilution of CTV-associated fluorescence.

(E) IL-10 was quantified in the co-culture supernatant by ELISA. Mean ± SEM values from triplicates are shown.

(F) Ten weeks old *SNCA* mice were treated with the intravenous (i.v.) injection of CAR-Treg (10^5 cells/mouse) expressing CAR 1A11 in the VH-VL conformation or only vehicle (non-treated). Ten weeks later, the MLNs were isolated and stimulated with peptides derived from unmodified hαSyn, 3NYhαSyn, or without peptides, and 24 h later, the IFN-γ production by CD8$^+$ T cells was analyzed by intracellular immunostaining followed by flow cytometry. Values are the percentage of IFN-γ$^+$ cells in the CD8$^+$ TCRβ$^+$ ZAq$^-$ population. Each symbol represents an individual mouse.

Mean ± SEM values are shown. *, $p < 0.05$; ***, $p < 0.001$; ****, $p < 0.0001$, compared with the control by one-way ANOVA followed by Dunnett's post-hoc test (B, C, and E) or by two-way ANOVA followed by Sidak's post-hoc test (F).

doses tested (10^4, 10^5, and 10^6 CAR-Treg cells per mouse) induced a significant decrease in microglial activation when all the anteroposterior sections were considered together (Figures S15B–S15D). To determine whether the therapeutic effect of CAR-Treg cells was sustained over time, we assessed the motor performance of a subset of mice at 30 weeks of age. The results showed that the motor function remained substantially improved, even 20 weeks after the administration of a single dose of 10^6 CAR-Treg cells per mouse (Figures 3C and 3D; Figure S16). To examine the distribution and persistence of CAR-Treg cells in different tissues over time following transfer into *SNCA* mice, we injected CD45.1$^+$ CAR-Treg cells into CD45.2$^+$ congenic recipients and quantified the relative abundance of donor cells among total CD4$^+$ T cells in blood, spleen, Peyer's patches, brain, spinal cord, intestine, cervical lymph nodes (CLNs), and MLNs. Very low levels ($\geq$0.05%) of CAR-Treg cells were detected in blood, spleen, CLNs, MLNs, and intestine one week after cell transfer, and these levels progressively declined at weeks 3 and 10 post-transfer (Figure S17). Notably, CAR-Treg abundance selectively increased in the brain

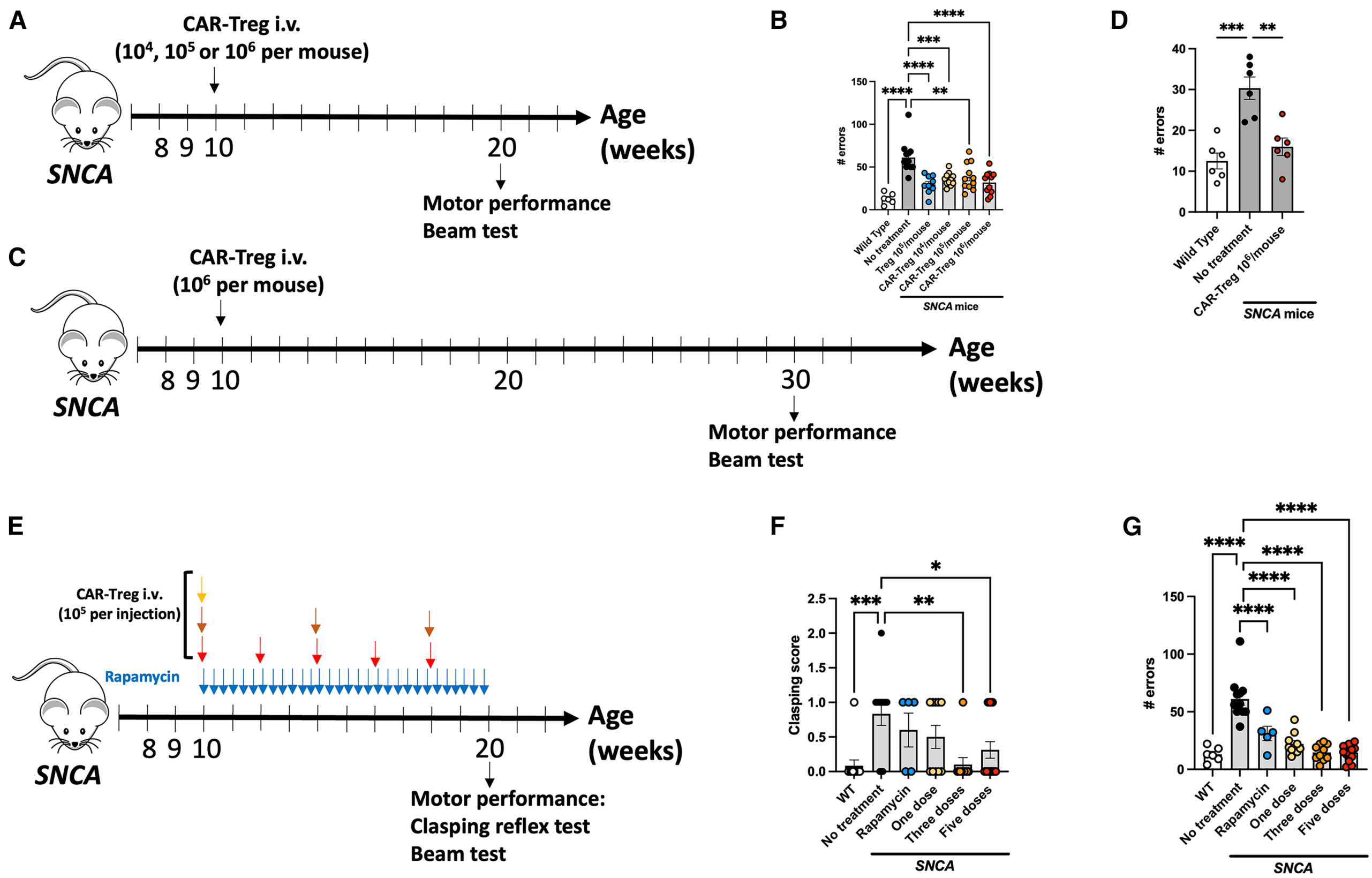

Figure 3. Evaluation of the efficacy of CAR-Treg therapy in *SNCA* mice at the level of motor performance

(A–D) 10-week-old *SNCA* mice (gray bars) were treated with a single intravenous (i.v.) injection of CAR-Treg expressing CAR1A11 in the VH-VL conformation (10^4, yellow symbols; 10^5, orange symbols; or 10^6 cells/mouse, red symbols), or polyclonal Treg (10^5 cells/mouse, blue symbols). Non-treated *SNCA* mice (black symbols) were used as controls. Age-matched wild-type mice (white bars and symbols) were also evaluated as controls.

(E–G) 10-week-old *SNCA* mice (gray bars) were treated with a single i.v. injection (at week 10; yellow symbols), three injections (at weeks 10, 14, and 18; orange symbols), or five injections (at weeks 10, 12, 14, 16, and 18; red symbols) of CAR-Treg (10^5 cells/mouse). *SNCA* mice treated with rapamycin (7.5 mg/kg intraperitonially [i.p.] three times a week for 10 weeks, blue symbols) or non-treated mice (black symbols) were used as controls. Age-matched wild-type mice (white bars and symbols) were also evaluated as controls.

(F and G) At week 20 (A, B, and E–G) or 30 (C and D), the motor performance was quantified by the hindlimb clasping reflex test (F) and the beam test (B, D, and G). (A), (C), and (E) illustrate the experimental strategy for the analysis shown in (B), (D), (F), and (G), respectively. (B), (D), and (G) show the total number of errors in all four tracts quantified in the beam test. (F) shows the clasping score. Each symbol represents an individual mouse (n = 5–16 mice per group). Mean ± SEM values are shown. *, $p < 0.05$; **, $p < 0.01$; ***, $p < 0.001$; ****, $p < 0.0001$, compared with control non-treated *SNCA* mice (black symbols), by Kruskal-Wallis test followed by Dunn's post-hoc test (F) or one-way ANOVA followed by Dunnet's post-hoc test (B, D, and G).

(up to 1% of total $CD4^+$ T cells) 3 weeks after transfer but was completely lost by week 10 (Figure S17). CAR-Treg cells were undetectable in the spinal cord at all assessed time points (Figure S17). Because the number of CAR-Treg cells declined over time in blood, gut, and brain, we sought to compare the therapeutic efficacy of administering different numbers of CAR-Treg injections within the treatment time frame in *SNCA* mice. We chose 10^5 CAR-Treg cells per mouse to determine the motor performance of *SNCA* mice receiving one (at week 10 of age), three (at weeks 10, 14, and 18 of age), or five injections (at weeks 10, 12, 14, 16, and 18 of age) of 10^5 CAR-Treg cells per mouse (Figure 3E). As a positive control we included a group of *SNCA* mice treated with rapamycin, which has been shown to reduce neuroinflammation and neurodegeneration in PD models.[32–34] To gain more robust conclusions on the motor performance in these experiments, in addition to the beam test, we used a second paradigm, the hindlimb clasping reflex test, which does not need a training stage and is associated with basal ganglia dysfunction.[35] Interestingly, although all administration regimes of the CAR-Treg therapy induced a significant reduction in the motor impairment in the beam test (Figure 3G; Figure S18), the administration of three and five doses of the therapy induced a significant improvement in the hindlimb clasping reflex test (Figure 3F). These differing conclusions obtained from the beam test and the hindlimb clasping reflex test following a single CAR-Treg injection in *SNCA* mice may be explained by the fact that the latter test is less sensitive and discriminative than the beam test.[36] Of note, five doses of the therapy had no better effects than three doses at the level of motor decline (Figures 3F and 3G). However, when neuroinflammation was evaluated in these animals, the analysis of activated microglia in single striatal sections as well as the analysis of

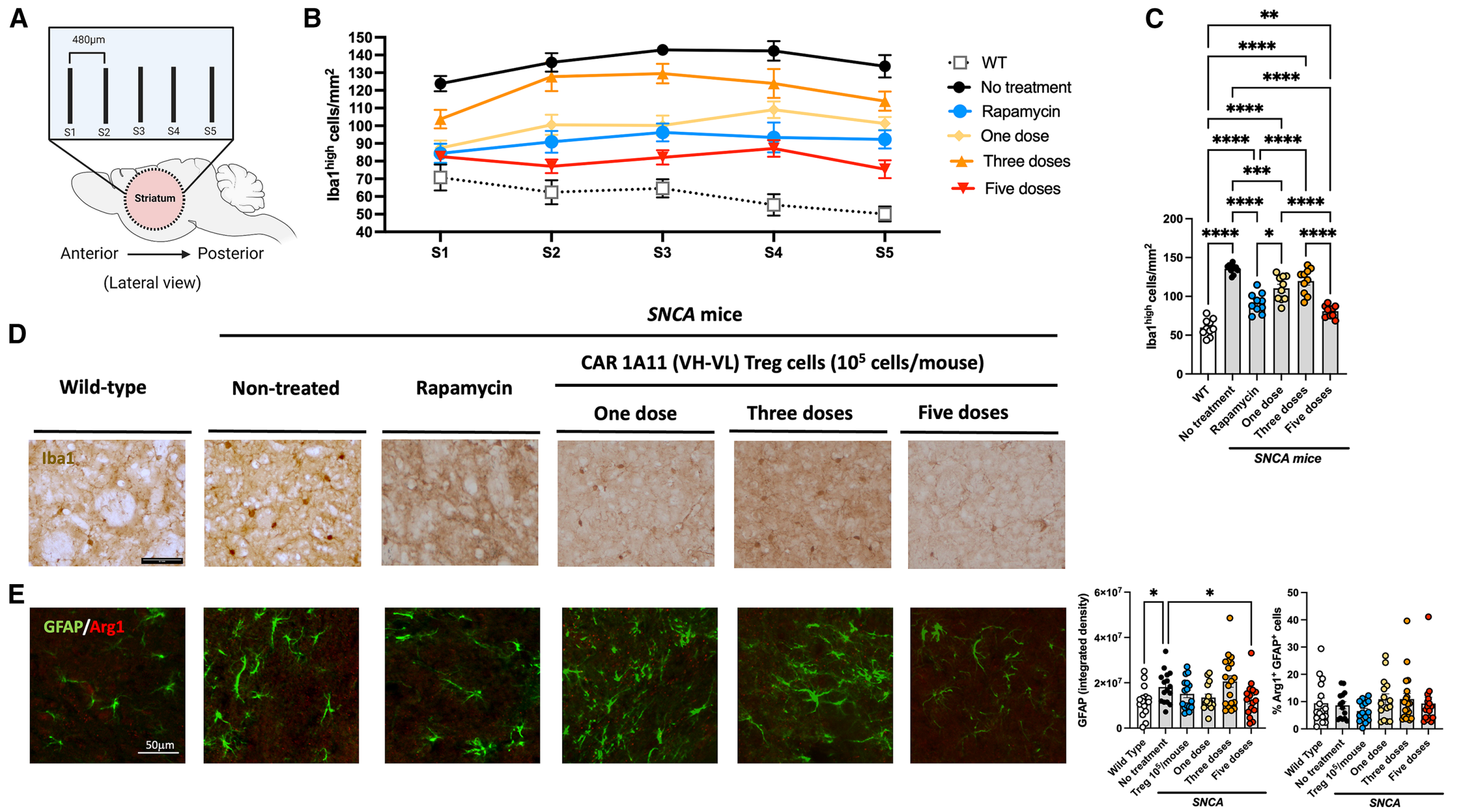

Figure 4. Evaluation of neuroinflammation based on the number of CAR-Treg injections in *SNCA* mice

(A–E) Ten weeks old *SNCA* mice were treated with a single intravenous (i.v.) injection (at week 10; beige), three injections (at weeks 10, 14, and 18; orange), or five injections (at weeks 10, 12, 14, 16, and 18; red) of CAR-Treg expressing CAR1A11 in the VH-VL conformation (10^5 cells/mouse). *SNCA* mice treated with rapamycin (A–D; 7.5 mg/kg intraperitoneally [i.p.] three times a week for 10 weeks, blue symbols) or polyclonal Treg (E; a single i.v. injection of 10^5 cells/mouse given at week 10 of age; blue symbols) or non-treated (black symbols) were used as controls.

(A–D) The extent of microgliosis was evaluated at 20 weeks by IHC analysis of Iba1 along the striatal antero-posterior axis.

(A) Scheme illustrating the analysis of five different sections (S1–S5), separated by 480 μm, along the striatum.

(B and C) Quantification of the number of cells displaying high immunoreactivity for Iba1 per area in every single section (B) or the average from all the sections together (C). Each symbol represents an individual mouse (n = 5–16 mice per group).

(D) Representative images from section 3 are shown for each experimental group. Scale bars, 100 μm.

(E) Astrogliosis was determined by immunofluorescence analysis of GFAP together with the anti-inflammatory marker arginase-1 (Arg1). Left: representative images; scale bars, 50 μm. Right: quantification of reactive astrocytes as the intensity of GFAP-associated fluorescence (integrated intensity), and the extent of anti-inflammatory astrocytes as the percentage of $Arg1^+$ cells from the $GFAP^+$ population.

Each symbol represents a striatal slice analyzed (n = 4–5 mice per group; 4 slices per mouse). In (B), (C), and (E), mean ± SEM values are shown. *, $p < 0.05$; **, $p < 0.01$; ***, $p < 0.001$; ****, $p < 0.0001$, by one-way ANOVA followed by Tukey's post-hoc test.

activated microglia in the whole anteroposterior striatal axis revealed a more potent therapeutic effect of the CAR-Treg therapy when administered in five doses compared with that administered in one or three doses (Figures 4B–4D). Morphometric analysis of striatal $Iba1^+$ cells revealed a lower degree of microglia with inflammatory shape (number of segment terminal points) in the *SNCA* mice receiving three or five doses of the CAR-Treg therapy, or polyclonal Treg, compared with the non-treated *SNCA* mice (Figure S19). To obtain a broader view of the neuroinflammatory landscape, we also analyzed striatal astrocytes by immunofluorescence for glial fibrillary acidic protein (GFAP) and the anti-inflammatory marker arginase-1 (Arg1). Consistent with the therapeutic effect in dampening microgliosis (Figures 4B–4D), the extent of astrogliosis observed in *SNCA* mice, as indicated by the intensity of GFAP expression, was significantly reduced only in mice receiving five doses of CAR-Treg therapy (Figure 4E). However, no significant differences were detected among the experimental groups when the subpopulation of anti-inflammatory astrocytes ($GFAP^+Arg1^+$ cells) was quantified (Figure 4E). Similarly, the analysis of synuclein pathology in the nigrostriatal pathway revealed that the burden of NαSyn (1A11 mAb immunoreactivity) and phosphorylated αSyn (pSer129-αSyn immunoreactivity) deposits was significantly reduced in the SN of mice receiving either five doses or three doses of CAR-Treg therapy (the latter only in the case of NαSyn) but not in mice receiving a single injection (Figure S20). In the striatum, a reduction in NαSyn deposits was observed only in mice treated with five CAR-Treg injections (Figure S20). Of note, the histopathological analysis of liver, spleen, kidney, lung, small intestine, and colon obtained from the mice receiving the 1, 3, or 5 doses of the CAR-Treg therapy (10^5 cells per mouse) revealed no evident signs of tissue damage (Figure S21). Thus, taken together, these results indicated that the administration of CAR-Treg is safe and induces a significant therapeutic effect, improving motor performance, dampening synuclein pathology, and decreasing the abundance of $Iba1^{high}$ cells within the

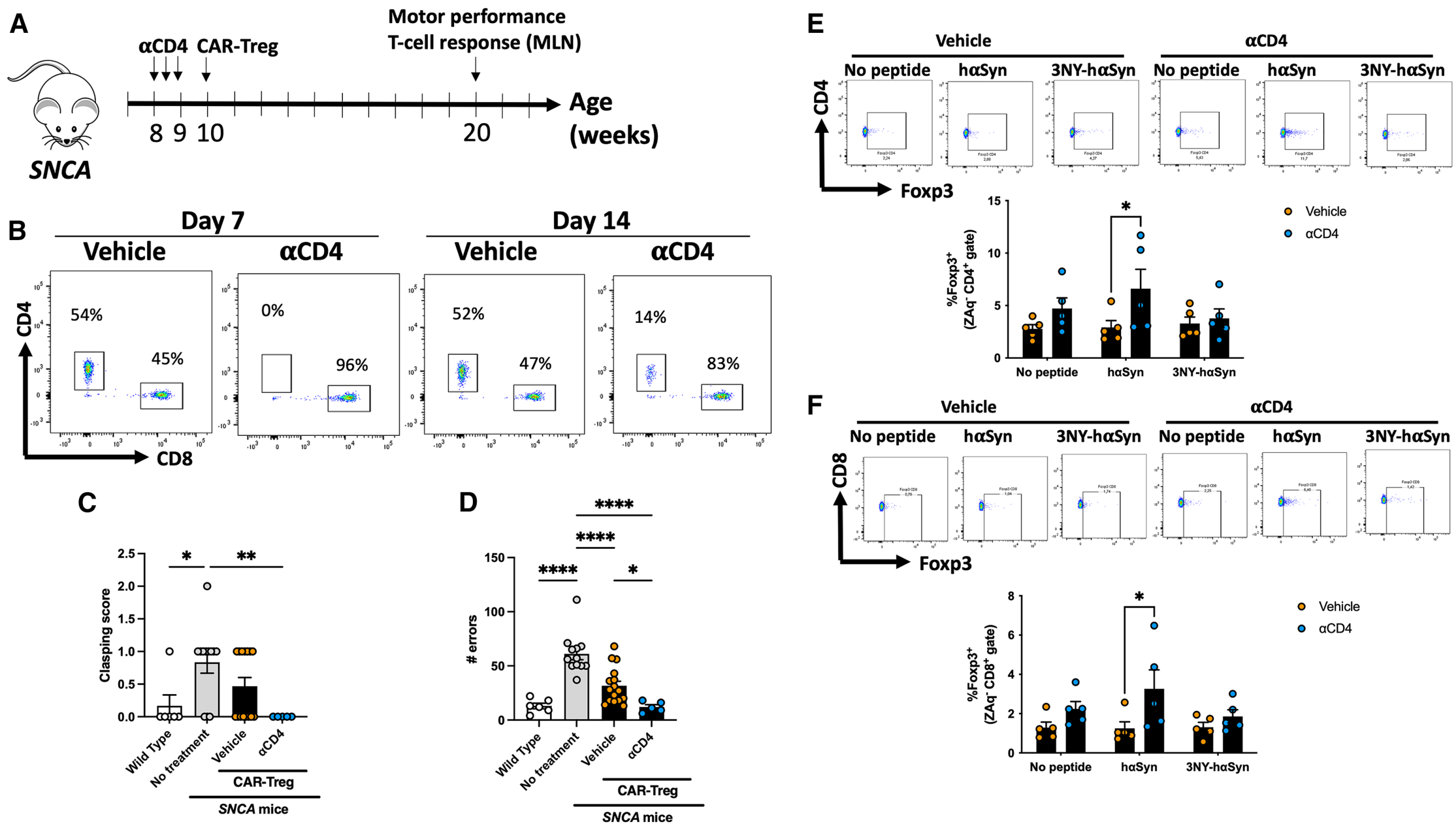

Figure 5. Transiently depleting CD4+ T cells improves the efficacy of CAR-Treg therapy in *SNCA* mice

(A–F) Eight weeks old *SNCA* mice received three injections of a depleting anti-CD4 mAb (100 μg/injection, intraperitoneal [i.p.]; blue symbols) or vehicle (orange symbols) separated for 24 h between injections. At week 10 of age, the mice were treated with intravenous (i.v.) CAR-Treg expressing CAR1A11 in the VH-VL conformation (10^5 cells/mouse). Age-matched *SNCA* (gray symbols) and wild-type (white symbols) mice without treatments were used as controls. At week 20 of age, the motor performance was quantified by the hindlimb clasping reflex test or the beam test.

(A) Illustration of the experimental strategy.

(B) Representative dot plots showing the percentage of $CD4^+$ and $CD8^+$ T cells from peripheral blood $TCR\beta^+$ lymphocytes 7 and 14 days after the treatment with anti-CD4 mAb or vehicle.

(C) The clasping score.

(D) Total number of errors in all four tracts.

(E and F) At week 20 of age, mononuclear cells were isolated from the mesenteric lymph nodes (MLNs) of mice treated with anti-CD4 mAb or vehicle and then with CAR-Treg. The cells were stimulated with peptides derived from unmodified hαSyn, 3NYhαSyn, or without peptides, and, 24 h later, the extent of Foxp3 expression in the $CD4^+$ (E) and $CD8^+$ (F) T cell populations was analyzed by intracellular immunostaining followed by flow cytometry. Values are the percentage of $Foxp3^+$ cells in the $CD4^+$ $TCR\beta^+$ ZAq^- (E) and $CD8^+$ $TCR\beta^+$ ZAq^- (F) populations. Top: representative dot plots; bottom: quantification.

Each symbol represents an individual mouse. $n = 5$–16 mice per group in (C) and (D); $n = 5$ mice per group in (E) and (F). Mean ± SEM values are shown. *, $p < 0.05$; **, $p < 0.01$; ****, $p < 0.0001$, by Kruskal-Wallis test (C), one-way ANOVA (D), or multiple *t* test (E and F).

striatum in *SNCA* mice, and the highest efficacy is achieved when five injections of 10^5 CAR-Treg cells are administered per mouse in the time frame of weeks 10–20 of age.

Lymphodepletion enhances CAR-T efficacy by eliminating endogenous lymphocytes that otherwise act as cytokine sinks, thereby increasing the availability of key homeostatic cytokines such as IL-2, IL-7, and IL-15 to support robust expansion and persistence of infused cells. In addition, it improves the trafficking capacity of adoptively transferred T cells, facilitating more efficient engagement with target tissues and enhancing the therapeutic impact.[37] Because previous studies have observed higher potency of T cell immunotherapy after lymphodepletion,[37] we wondered whether the transient depletion of $CD4^+$ T cells improved the efficacy of the CAR-Treg therapy. To this end, we treated *SNCA* mice with a depleting anti-CD4 antibody for 3 days and, 10 days later, performed the transfer of 10^5 CAR-Treg cells per mouse (Figure 5A). Of note, the transfer of therapeutic cells was executed before endogenous $CD4^+$ T cells repopulated peripheral blood (Figure 5B). This pre-treatment improved the efficacy of CAR-Treg therapy at the level of motor performance, as shown by the hindlimb clasping reflex test (Figure 5C) and the beam test (Figure 5D). Additionally, transient $CD4^+$ T cell depletion increased the proportion of $Foxp3^+$ $CD4^+$ and $CD8^+$ T cells in the CLNs (Figures 5E and 5F), although this effect was not reproduced in the MLNs (Figure S22B). The frequency of inflammatory $CD4^+$ and $CD8^+$ T cells was already low in the CLNs and MLNs of mice treated with CAR-Treg therapy alone (vehicle), and it was not further reduced by transient $CD4^+$ T cell depletion (Figures S22A and S22B). Therefore, the beneficial effect of lymphodepletion in potentiating CAR-Treg therapy appears to be associated with a higher abundance of anti-inflammatory lymphocytes in the CLNs at the 20-week time point.

CellPress
OPEN ACCESS

CAR-Treg therapy specific to 3NY-hαSyn reduces neurodegeneration and neuroinflammation in the AAV-hαSyn mouse model

To validate the relevance of the 3NY-hαSyn-specific T cell response in another preclinical model of PD, we chose the AAV-hαSyn mouse model, which develops not only neuroinflammation but also neurodegeneration 15 weeks after the stereotaxic surgery.[38] We first attempted to induce an immunosuppressive response selectively directed to 3NY-hαSyn by immunizing the AAV-hαSyn mice with this peptide (Table S1) under a regime that promote Treg responses.[8,39] The results showed a marked inhibition of the neurodegeneration of nigral dopaminergic neurons when the mice overexpressing hαSyn were immunized with the 3NY-hαSyn peptide and complete Freund's adjuvant CFA, but not with CFA alone (Figure S23). We also observed that the epitope recognized by the 1A11 mAb was increased on the ipsilateral side (Figure S24). Thus, these results suggest that modulation of the adaptive immune response to 3NYαSyn in the periphery dampens the neurodegenerative process involved in the AAV-hαSyn preclinical model.

To test the therapeutic potential of Treg cells expressing CAR-1A11, PD was induced in mice by AAV-hαSyn, and 3 weeks later, the mice received the intravenous (i.v.) transfer of Treg expressing CAR-1A11 bearing the scFv assembled in the VH-VL or VL-VH conformation. To control the relevance of CAR specificity, a group of mice were selected for the transfer of Treg cells transduced with a CAR specific to CEA, an antigen unrelated to PD.[40] Sixteen weeks after the stereotaxic surgery, the extent of dopaminergic neuron loss was determined by immunohistochemical analysis of tyrosine hydroxylase (TH). The results showed that the transfer of Treg cells expressing CAR-1A11 significantly attenuated the loss of dopaminergic neurons in the SN, which was ≈31% in AAV-hαSyn mice without treatment and only ≈18% or ≈19% in those AAV-hαSyn mice who received the CAR-Treg bearing the CAR1A11 with the VH-VL or the VL-VH conformation, respectively (Figure 6A). Conversely, the treatment of AAV-hαSyn mice with Treg expressing the CAR-CEA resulted in the loss of ≈35% dopaminergic neurons in the SN, which was not different from the extent of neurodegeneration observed in untreated AAV-hαSyn mice (Figure 6A). To further explore the extent of neurodegeneration, we assessed the loss of dopaminergic terminals in the striatum by analyzing immunoreactivity associated with the dopamine transporter (DAT). However, this analysis revealed only one significant difference: mice treated with CAR-1A11 (VH-VL conformation) had a higher DAT density than the non-treated AAV-hαSyn mice (Figure 6B). We performed additional analyses in which nigral sections were co-immunostained for the pan-neuronal marker NeuN and the dopaminergic neuronal marker TH (Figure S25). The numbers of $NeuN^+$ and TH^+ cells were subsequently quantified. Importantly, the reduction in TH^+ cells correlated with a decrease in $NeuN^+$ cells (Figure 6C), suggesting that the reduced number of TH^+ cells reflects neuronal loss, rather than a mere downregulation of TH expression.

To analyze the involvement of neuroinflammation in the reduction of neurodegeneration of dopaminergic neurons in AAV-hαSyn mice receiving the CAR-Treg therapy, we next analyzed the extent of microglial activation in the same experimental groups described above. For this purpose, microglial activation was quantified in the striatum as the density of cells expressing high levels of Iba1 and displaying ameboid shapes, as described before.[41] The results showed that the mice treated with Treg expressing CAR-1A11 bearing the scFv assembled in either the VH-VL conformation or the VL-VH conformation presented a significant attenuation of microglial activation in the striatum (Figure 6D). Indeed, while microgliosis in the AAV-hαSyn mice that did not receive Treg transfer reached ≈222% relative to microgliosis observed in the sham group; AAV-hαSyn mice receiving the CAR-1A11 Treg displayed ≈100% (VH-VL conformation) and ≈123% (VL-VH conformation) microglial activation (Figure 6D). AAV-hαSyn mice receiving the transfer of Treg cells expressing the CAR-CEA presented a mild reduction of microgliosis (≈150%; Figure 6D), suggesting that, irrespective to the specificity, Treg treatment was able to limit neuroinflammation to a low degree. Thus, these results indicate that the CAR-Treg therapy dampens the microglial activation in the AAV-hαSyn mouse model. In addition, the CAR-Treg therapy reduced the extent of motor impairment in the group of AAV-hαSyn mice receiving the CAR-1A11 Treg in VL-VH conformation, although the group of mice receiving CAR-1A11 Treg in VH-VL conformation did not show a significant reduction (Figure 6E), probably due to the high dispersion of data. To further explore the mechanism of how Treg cells bearing a CAR specific to 3NY-hαSyn might attenuate neuroinflammation, we next analyzed the extent of activation of astrocytes, which play a pivotal role in this process.[42] To this end, the extent of astrogliosis was quantified as the density of GFAP expression in the striatum, as described.[41] Interestingly, the results showed that the AAV-hαSyn mice receiving Treg bearing CAR-1A11 with either VH-VL or VL-VH scFv conformation displayed a lower extent of astrogliosis in the striatum (Figure S26). In contrast, the extent of astrogliosis was not significantly changed in those AAV-hαSyn mice that received Treg bearing the CAR-CEA (Figure S26). Altogether, these results indicate that using Treg cells expressing a CAR with specificity to 3NY-hαSyn reduces the extent of neurodegeneration and decreases the abundance of $Iba1^{high}$ cells and GFAP immunoreactivity in the striatum in the mouse model of PD induced by AAV-hαSyn.

DISCUSSION

Here, we propose that generating CAR-Treg specific to the pathogenic forms of αSyn represents a promising therapeutic strategy for treating PD patients. In addition, in this study, we obtained a mAb specific to the pathogenic forms of αSyn, which displays a promising diagnostic potential as its immunoreactivity in serum is higher in PD patients than in HCs and correlates with PD severity. Although these observations were obtained from a small cohort of PD patients and HCs and did not address the specificity of the 1A11 mAb for PD relative to other synucleinopathies, we aim to evaluate its diagnostic potential in human biological fluids, including serum and cerebrospinal fluid (CSF), in a larger cohort of patients with PD and other synucleinopathies in future studies.

Here, we developed an experimental immunotherapy for PD, which not only efficiently attenuated the neuroinflammatory

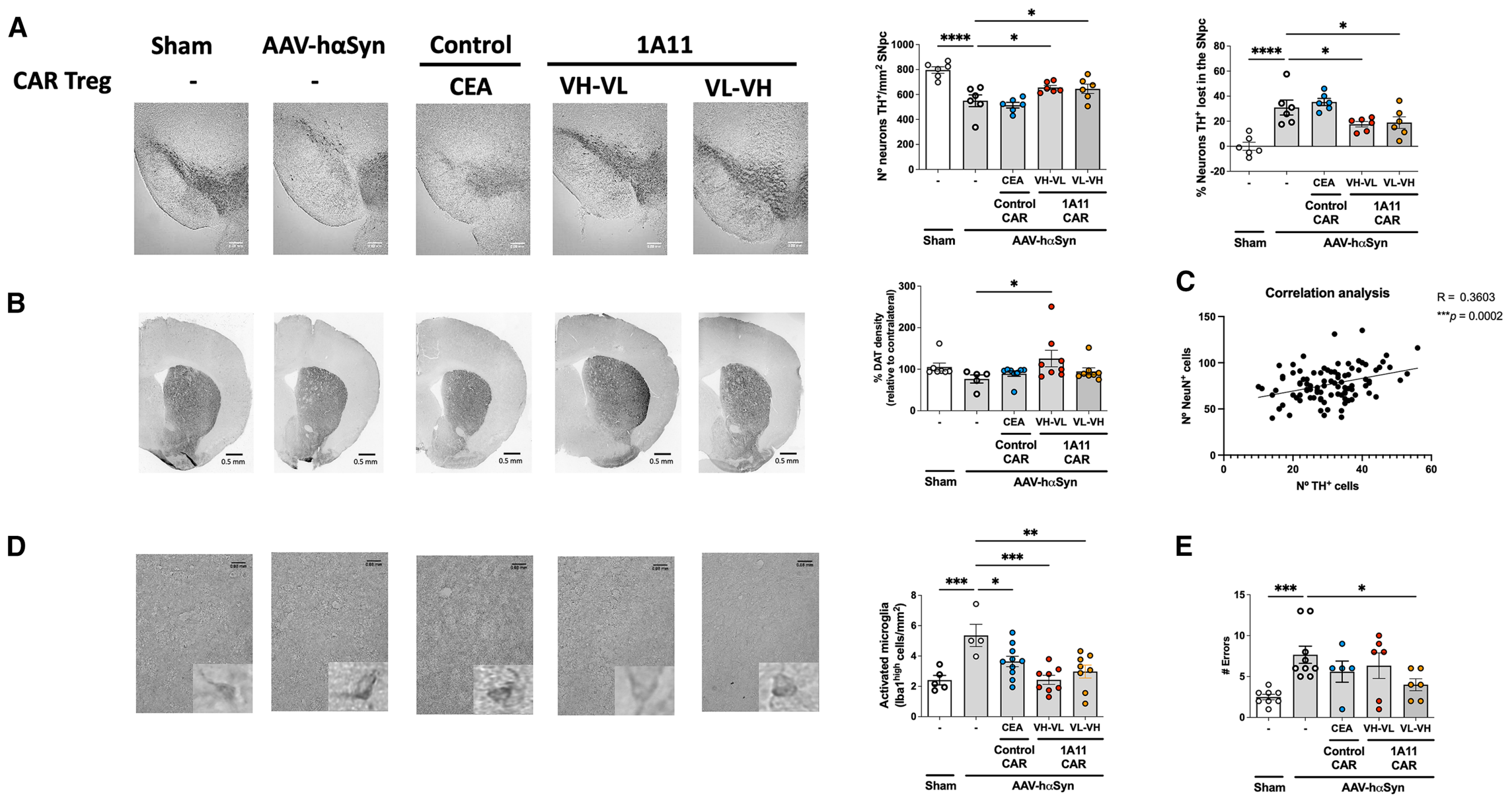

Figure 6. CAR-Treg therapy attenuates neurodegeneration, neuroinflammation, and motor impairment in mice treated with AAV-hαSyn

(A–E) Ten-week-old male wild-type C57BL/6 mice received the stereotaxic inoculation of AAV encoding human αSyn (AAV-hαSyn; 10^{10} vg/mouse; gray bars) or vehicle (sham; white bar) into the right substantia nigra (SN). Three weeks after the surgery, the mice received intravenous (i.v.) injection of CAR-Treg (3×10^4 cells per mouse) expressing CAR1A11 in the VH-VL (red symbols) or VL-VH (orange symbols) conformation. As a control group, some mice received the i.v. injection of CAR-Treg (3×10^4 cells per mouse) expressing CAR-CEA (control CAR; blue symbols). Fifteen weeks after the stereotaxic surgery, the motor performance was evaluated by the beam test, and one week later, the extent of dopaminergic neurodegeneration and neuroinflammation was quantified in the nigrostriatal pathway.

(A) Neurodegeneration was evaluated by immunohistochemical analysis of tyrosine hydroxylase (TH) in the right SN pars compacta (SNpc) and quantified as the number of neurons per area (middle) or as the percentage of neurons lost (right).

(B) Loss of dopaminergic terminals was estimated by immunofluorescence analysis of dopamine transporter (DAT) in the striatum.

(C) Nigral sections were co-immunostained for TH and the pan-neuronal marker NeuN, and the TH^+ cells and $NeuN^+$ cells were quantified. Correlation analysis of the number of $NeuN^+$ cells and TH^+ cells is shown.

(D) Neuroinflammation was assessed as microglial activation determined by immunohistochemical analysis of Iba1 in the striatum.

(E) Motor performance quantified as the number of total errors in the beam test.

(A), (B), and (D) show representative images for each experimental group (left); scale bars, 80 μm (A and D) and 500 μm (B). In (A)–(D), each symbol represents an individual mouse. Data from 6 (A), 5–8 (B), 4–5 (C), 4–10 (D), and 5–9 (E) mice per group are shown. In (A), (B), (D), and (E), mean ± SEM values are shown. *, $p < 0.05$; ***, $p < 0.001$; ****, $p < 0.0001$, compared with the group of mice receiving AAV-hαSyn without treatment (gray bar, white symbols), by one-way ANOVA followed by Dunnett's post-hoc test. (C) p and R values are shown for the correlation analysis performed with Pearson's correlation test.

process and the neurodegeneration of dopaminergic neurons in the SN but also represents a safe therapeutic strategy from different points of view. First, our therapeutic approach here is based on the generation of Treg response highly specific to 3NY-hαSyn but not to unmodified hαSyn or to other antigens containing nitro-tyrosine residues. Thereby, this immunotherapy should not actively develop tolerogenic responses to unmodified hαSyn in healthy individuals but only in response to the pathogenic NαSyn present in PD patients, thus representing a safe therapy from the point of view of antigen specificity. Secondly, our therapeutic strategy does not include the usage of CFA, which is highly toxic to humans.[43] Third, our immunotherapy is based on CAR technology, which has been proven to be safe in humans and approved for use in cancer patients by the FDA.[44] Fourth, here we tested the doses at which the administered CAR-Treg therapy efficiently dampens disease development and found that it was safe in several organs analyzed, including the liver, spleen, kidney, lung, small intestine, and colon. Therefore, the CAR-Treg therapy presented here represents an efficient immunotherapy attenuating neurodegeneration, neuroinflammation, and motor decline in preclinical models of PD and constitutes a safe strategy from multiple points of view. Future clinical research should evaluate the therapeutic potential of CAR-Treg therapy in humans, initially through *in vitro* suppression assays using peripheral blood mononuclear cells obtained from PD patients, followed by clinical trials to assess the safety, tolerability, and efficacy of CAR-Treg cells.

Limitations of the study

Our results show that five injections of CAR-Treg cells induce a significantly greater therapeutic effect than one or three injections, as reflected by the extent of microgliosis attenuation (Figure 4). This effect is likely due to the fact that CAR-Treg cells

reach the brain 3 weeks after adoptive transfer and subsequently disappear from this tissue (Figure S17). Further studies should determine whether this outcome is due to cell death, phenotypic instability, and/or migration to other tissues. Understanding these mechanisms could provide crucial insights to improve the efficacy of CAR-Treg therapy.

The main parameter used in this study to assess the effect of CAR-Treg therapy on neuroinflammatory processes was the abundance of Iba1^{high} cells within the striatum, a feature commonly associated with the increased activity of microglia and other myeloid cell populations. However, this approach does not allow discrimination between resident microglia, border-associated macrophages, and peripheral monocytes/macrophages infiltrating the brain. Therefore, future mechanistic studies will be required to determine the specific impact of CAR-Treg therapy on each of these myeloid cell populations and to further define the cellular mechanisms underlying its therapeutic effects.

The present study provides evidence that CAR-Treg therapy is safe based on the histological analysis of several organs, including the spleen, liver, kidney, lung, small intestine, and colon (Figure S21). Although this qualitative analysis suggests that the therapy is generally not harmful to major organs, future research should include more specific and quantitative analyses of different cell populations across multiple tissues in order to rule out potential detrimental side effects.

Both *SNCA* transgenic mice[45,46] and AAV-αSyn mice[20,47] are valuable animal models of PD that involve the participation of inflammatory T cells infiltrating the brain. Nevertheless, these animal models do not reproduce all the features of PD; rather, they capture only selected aspects of the pathology. Thus, their generalizability to the human, chronic, and heterogeneous diseases is limited. Despite evidence of autoreactive T cells specific to αSyn-derived antigens in humans,[10] their role as drivers of pathology has not been firmly established. Therefore, another limitation of the present study is that the translational significance of this CAR-Treg therapy remains uncertain. The present study includes an exploratory analysis of the immunoreactivity of the 1A11 mAb in serum samples from a small cohort of HC and PD patients (Figures 1D and 1E); however, it does not evaluate the specificity of this immunoreactivity in relation to other synucleinopathies. Future studies should, therefore, evaluate the diagnostic potential of the 1A11 mAb in a larger cohort of HC and patients, as well as its ability to discriminate between samples from patients with PD, dementia with Lewy bodies (DLB), multiple system atrophy (MSA), and Alzheimer disease (AD).

In conclusion, our findings demonstrate that inducing an NαSyn-specific Treg response using CAR technology exerts a therapeutic effect by attenuating neurodegeneration of nigral dopaminergic neurons, reducing neuroinflammation, and ameliorating motor decline in two distinct preclinical models of PD. This work represents a preclinical proof-of-concept study proposing CAR technology as a potential therapeutic strategy for PD. Our findings provide robust evidence of its efficacy while also suggesting an overall favorable safety profile. However, from a translational perspective, the present study has several limitations that should be addressed in future research to facilitate the progression of CAR-Treg therapy from mouse models to human application.

RESOURCE AVAILABILITY

Lead contact

Requests for further information, resources, and reagents should be directed to and will be fulfilled by the lead contact, Rodrigo Pacheco (rpacheco@cienciavida.org).

Materials availability

There are restrictions to the availability of vectors encoding CAR1A11 (in VL-VH or VH-VL conformation of the scFv) because this therapeutic approach is under IP protection (patent application).

Data and code availability

- Original dot blot images, western blots images, microscopy data, and other data reported in this paper will be shared by the lead contact upon request.
- This paper does not report original code.
- Any additional information required to reanalyze the data reported in this paper is available from the lead contact upon request.

ACKNOWLEDGMENTS

We thank Dr. Hinrich Abken for providing us with the pBullet vector, Miss María José Fuenzalida for her technical assistance in cell sorting, and Sebastián Valenzuela and Micaela Ricca for their valuable veterinary assistance in our animal facility. We thank Sebastián Belmar and Miguel Vargas form MERKEN biotech for their technical help in histological analysis of the 1A11 mAb immunoreactivity in colonic tissue. This work was supported by the grants from Agencia Nacional de Investigación y Desarrollo: "Financiamiento Basal para Centros Científicos y Tecnológicos de Excelencia" Centro Ciencia & Vida (FB210008) (to Fundación Ciencia & Vida), FONDECYT (1250021 to R.P.; 1251312 to A. Lladser; and 3220788 to S.H.), and FONDEF (ID22I10070 to R.P.). This study was also supported by The Michael J. Fox Foundation for Parkinson's Research grant (MJFF-15076 to R.P.).

AUTHOR CONTRIBUTIONS

Conceptualization: R.P. and A. Lladser.; methodology, V.U., E.L., S.E., A.C., F.A.-A., D. Franz, V.S.-G., M.I.B., D. Figueroa, A. Lladser, S.H., Z.M., C.T.-R., A. Loyola, and S.A.Q.; investigation, V.U., D.E., S.E., O.C.-V., E.L., A.C., F.A.-A., D. Franz, and D. Figueroa; visualization, D.E., S.E., V.U., and R.P.; funding acquisition, R.P.; project administration, R.P.; supervision, R.P.; writing – original draft, R.P.; writing – review & editing, R.P., M.I.B., E.L., and V.S.-G.

DECLARATION OF INTERESTS

A. Lladser and R.P. are named inventors on a pending PCT patent application describing the therapeutic use of CAR-Treg with specificity to neoantigens derived from αSyn as treatment for PD. The PCT application has not yet been published and is currently under review.

DECLARATION OF GENERATIVE AI AND AI-ASSISTED TECHNOLOGIES IN THE WRITING PROCESS

During the preparation of this work, the authors used Chat GPT 5.2 in order to improve the grammar and for redaction in some parts of the text. After using this tool/service, the authors reviewed and edited the content as needed and take full responsibility for the content of the published article.

STAR★METHODS

Detailed methods are provided in the online version of this paper and include the following:

- KEY RESOURCES TABLE
- EXPERIMENTAL MODEL AND STUDY PARTICIPANT DETAILS
  - Animals
  - Human subjects
- METHOD DETAILS
  - Generation of monoclonal antibodies specific to nitrated α-synuclein
  - Determining 1A11 mAb immunoreactivity in human serum by dot-blot
  - Western blot analysis
  - Chimeric antigen receptor engineering and production
  - Transduction of T-cells with CARs and adoptive transfer
  - Determination of the CAR1A11 affinity
  - *In vitro* activation assay for CAR-Treg
  - *In vitro* suppression assay for CAR-Treg
  - Generation and *in vitro* activation assays of human CAR-Treg
  - Mouse models and treatments
  - Transient depletion of CD4⁺ T-cells
  - Tracking CAR-Treg distribution *in vivo*
  - Flow cytometry
  - Tissue processing
  - Immunohistochemical and immunofluorescent analysis
  - Beam test
  - Hindlimb clasping reflex
- QUANTIFICATION AND STATISTICAL ANALYSIS
- ADDITIONAL RESOURCES

SUPPLEMENTAL INFORMATION

Supplemental information can be found online at https://doi.org/10.1016/j.xcrm.2026.102983.

Received: May 28, 2025
Revised: May 12, 2026
Accepted: July 16, 2026
Published: August 12, 2026

REFERENCES

1. Roodveldt, C., Bernardino, L., Oztop-Cakmak, O., Dragic, M., Fladmark, K.E., Ertan, S., Aktas, B., Pita, C., Ciglar, L., Garraux, G., et al. (2024). The immune system in Parkinson's disease: what we know so far. Brain *147*, 3306–3324. https://doi.org/10.1093/brain/awae177.
2. González, H., and Pacheco, R. (2014). T-cell-mediated regulation of neuroinflammation involved in neurodegenerative diseases. J. Neuroinflammation *11*, 201. https://doi.org/10.1186/s12974-014-0201-8.
3. González, H., Contreras, F., and Pacheco, R. (2015). Regulation of the Neurodegenerative Process Associated to Parkinson's Disease by CD4+ T-cells. J. Neuroimmune Pharmacol. *10*, 561–575. https://doi.org/10.1007/s11481-015-9618-9.
4. Giasson, B.I., Duda, J.E., Murray, I.V., Chen, Q., Souza, J.M., Hurtig, H.I., Ischiropoulos, H., Trojanowski, J.Q., and Lee, V.M. (2000). Oxidative damage linked to neurodegeneration by selective alpha-synuclein nitration in synucleinopathy lesions. Science *290*, 985–989. https://doi.org/10.1126/science.290.5493.985.
5. Benner, E.J., Banerjee, R., Reynolds, A.D., Sherman, S., Pisarev, V.M., Tsiperson, V., Nemachek, C., Ciborowski, P., Przedborski, S., Mosley, R.L., and Gendelman, H.E. (2008). Nitrated alpha-synuclein immunity accelerates degeneration of nigral dopaminergic neurons. PLoS One *3*, e1376. https://doi.org/10.1371/journal.pone.0001376.
6. Yu, Z., Xu, X., Xiang, Z., Zhou, J., Zhang, Z., Hu, C., and He, C. (2010). Nitrated alpha-synuclein induces the loss of dopaminergic neurons in the substantia nigra of rats. PLoS One *5*, e9956. https://doi.org/10.1371/journal.pone.0009956.
7. Pacheco, R. (2021). T-cell based immunotherapies for Parkinson's disease. Explor. Neuroprotective Ther. *1*, 72–85. https://doi.org/10.37349/ent.2021.00007.
8. Christiansen, J.R., Olesen, M.N., Otzen, D.E., Romero-Ramos, M., and Sanchez-Guajardo, V. (2016). alpha-Synuclein vaccination modulates regulatory T cell activation and microglia in the absence of brain pathology. J. Neuroinflammation *13*, 74. https://doi.org/10.1186/s12974-016-0532-8.
9. Reynolds, A.D., Stone, D.K., Hutter, J.A.L., Benner, E.J., Mosley, R.L., and Gendelman, H.E. (2010). Regulatory T cells attenuate Th17 cell-mediated nigrostriatal dopaminergic neurodegeneration in a model of Parkinson's disease. J. Immunol. *184*, 2261–2271. https://doi.org/10.4049/jimmunol.0901852.
10. Sulzer, D., Alcalay, R.N., Garretti, F., Cote, L., Kanter, E., Agin-Liebes, J., Liong, C., McMurtrey, C., Hildebrand, W.H., Mao, X., et al. (2017). T cells from patients with Parkinson's disease recognize alpha-synuclein peptides. Nature *546*, 656–661. https://doi.org/10.1038/nature22815.
11. Reynolds, A.D., Glanzer, J.G., Kadiu, I., Ricardo-Dukelow, M., Chaudhuri, A., Ciborowski, P., Cerny, R., Gelman, B., Thomas, M.P., Mosley, R.L., and Gendelman, H.E. (2008). Nitrated alpha-synuclein-activated microglial profiling for Parkinson's disease. J. Neurochem. *104*, 1504–1525. https://doi.org/10.1111/j.1471-4159.2007.05087.x.
12. Reynolds, A.D., Kadiu, I., Garg, S.K., Glanzer, J.G., Nordgren, T., Ciborowski, P., Banerjee, R., and Gendelman, H.E. (2008). Nitrated alpha-synuclein and microglial neuroregulatory activities. J. Neuroimmune Pharmacol. *3*, 59–74. https://doi.org/10.1007/s11481-008-9100-z.
13. Reynolds, A.D., Stone, D.K., Mosley, R.L., and Gendelman, H.E. (2009). Nitrated {alpha}-synuclein-induced alterations in microglial immunity are regulated by CD4+ T cell subsets. J. Immunol. *182*, 4137–4149. https://doi.org/10.4049/jimmunol.0803982.
14. Barcia, C., Ros, C.M., Annese, V., Gómez, A., Ros-Bernal, F., Aguado-Llera, D., Martínez-Pagán, M.E., de Pablos, V., Fernandez-Villalba, E., and Herrero, M.T. (2012). IFN-gamma signaling, with the synergistic contribution of TNF-alpha, mediates cell specific microglial and astroglial activation in experimental models of Parkinson's disease. Cell Death Dis. *3*, e379. https://doi.org/10.1038/cddis.2012.123.
15. González, H., Contreras, F., Prado, C., Elgueta, D., Franz, D., Bernales, S., and Pacheco, R. (2013). Dopamine Receptor D3 Expressed on CD4+ T Cells Favors Neurodegeneration of Dopaminergic Neurons during Parkinson's Disease. J. Immunol. *190*, 5048–5056. https://doi.org/10.4049/jimmunol.1203121.
16. González, H., Elgueta, D., Montoya, A., and Pacheco, R. (2014). Neuroimmune regulation of microglial activity involved in neuroinflammation and neurodegenerative diseases. J. Neuroimmunol. *274*, 1–13. https://doi.org/10.1016/j.jneuroim.2014.07.012.
17. Matheoud, D., Cannon, T., Voisin, A., Penttinen, A.M., Ramet, L., Fahmy, A.M., Ducrot, C., Laplante, A., Bourque, M.J., Zhu, L., et al. (2019). Intestinal infection triggers Parkinson's disease-like symptoms in Pink1(-/-) mice. Nature *571*, 565–569. https://doi.org/10.1038/s41586-019-1405-y.
18. Brochard, V., Combadiere, B., Prigent, A., Laouar, Y., Perrin, A., Beray-Berthat, V., Bonduelle, O., Alvarez-Fischer, D., Callebert, J., Launay, J.M., et al. (2009). Infiltration of CD4+ lymphocytes into the brain contributes to neurodegeneration in a mouse model of Parkinson disease. J. Clin. Investig. *119*, 182–192.
19. Karikari, A.A., McFleder, R.L., Ribechini, E., Blum, R., Bruttel, V., Knorr, S., Gehmeyr, M., Volkmann, J., Brotchie, J.M., Ahsan, F., et al. (2022). Neurodegeneration by alpha-synuclein-specific T cells in AAV-A53T-alpha-synuclein Parkinson's disease mice. Brain Behav. Immun. *101*, 194–210. https://doi.org/10.1016/j.bbi.2022.01.007.

20. Williams, G.P., Schonhoff, A.M., Jurkuvenaite, A., Gallups, N.J., Standaert, D.G., and Harms, A.S. (2021). CD4 T cells mediate brain inflammation and neurodegeneration in a mouse model of Parkinson disease. Brain *144*, 2047–2059. https://doi.org/10.1093/brain/awab103.

21. Ito, M., Komai, K., Mise-Omata, S., Iizuka-Koga, M., Noguchi, Y., Kondo, T., Sakai, R., Matsuo, K., Nakayama, T., Yoshie, O., et al. (2019). Brain regulatory T cells suppress astrogliosis and potentiate neurological recovery. Nature *565*, 246–250. https://doi.org/10.1038/s41586-018-0824-5.

22. Sadelain, M., Brentjens, R., and Rivière, I. (2013). The basic principles of chimeric antigen receptor design. Cancer Discov. *3*, 388–398. https://doi.org/10.1158/2159-8290.CD-12-0548.

23. Lee, D.W., Barrett, D.M., Mackall, C., Orentas, R., and Grupp, S.A. (2012). The future is now: chimeric antigen receptors as new targeted therapies for childhood cancer. Clin. Cancer Res. *18*, 2780–2790. https://doi.org/10.1158/1078-0432.CCR-11-1920.

24. Wang, L., Magen, I., Yuan, P.Q., Subramaniam, S.R., Richter, F., Chesselet, M.F., and Taché, Y. (2012). Mice overexpressing wild-type human alpha-synuclein display alterations in colonic myenteric ganglia and defecation. Neuro Gastroenterol. Motil. *24*, e425–e436. https://doi.org/10.1111/j.1365-2982.2012.01974.x.

25. Sakaguchi, S., Vignali, D.A.A., Rudensky, A.Y., Niec, R.E., and Waldmann, H. (2013). The plasticity and stability of regulatory T cells. Nat. Rev. Immunol. *13*, 461–467. https://doi.org/10.1038/nri3464.

26. Ibrahimi, A., Vande Velde, G., Reumers, V., Toelen, J., Thiry, I., Vandeputte, C., Vets, S., Deroose, C., Bormans, G., Baekelandt, V., et al. (2009). Highly efficient multicistronic lentiviral vectors with peptide 2A sequences. Hum. Gene Ther. *20*, 845–860. https://doi.org/10.1089/hum.2008.188.

27. Contreras, F., Prado, C., González, H., Franz, D., Osorio-Barrios, F., Osorio, F., Ugalde, V., Lopez, E., Elgueta, D., Figueroa, A., et al. (2016). Dopamine Receptor D3 Signaling on CD4+ T Cells Favors Th1- and Th17-Mediated Immunity. J. Immunol. *196*, 4143–4149. https://doi.org/10.4049/jimmunol.1502420.

28. Choi, I., Zhang, Y., Seegobin, S.P., Pruvost, M., Wang, Q., Purtell, K., Zhang, B., and Yue, Z. (2020). Microglia clear neuron-released alpha-synuclein via selective autophagy and prevent neurodegeneration. Nat. Commun. *11*, 1386. https://doi.org/10.1038/s41467-020-15119-w.

29. Sampson, T.R., Debelius, J.W., Thron, T., Janssen, S., Shastri, G.G., Ilhan, Z.E., Challis, C., Schretter, C.E., Rocha, S., Gradinaru, V., et al. (2016). Gut Microbiota Regulate Motor Deficits and Neuroinflammation in a Model of Parkinson's Disease. Cell *167*, 1469–1480.e12. https://doi.org/10.1016/j.cell.2016.11.018.

30. Manjarres, Z., Calvo, M., and Pacheco, R. (2023). Regulation of Pain Perception by Microbiota in Parkinson Disease. Pharmacol. Rev. *76*, 7–36. https://doi.org/10.1124/pharmrev.122.000674.

31. Huang, Y., Liu, Z., Cao, B.B., Qiu, Y.H., and Peng, Y.P. (2020). Treg Cells Attenuate Neuroinflammation and Protect Neurons in a Mouse Model of Parkinson's Disease. J. Neuroimmune Pharmacol. *15*, 224–237. https://doi.org/10.1007/s11481-019-09888-5.

32. Zhang, G., Yin, L., Luo, Z., Chen, X., He, Y., Yu, X., Wang, M., Tian, F., and Luo, H. (2021). Effects and potential mechanisms of rapamycin on MPTP-induced acute Parkinson's disease in mice. Ann. Palliat. Med. *10*, 2889–2897. https://doi.org/10.21037/apm-20-1096.

33. Zhang, Y., He, X., Wu, X., Lei, M., Wei, Z., Zhang, X., Wen, L., Xu, P., Li, S., and Qu, S. (2017). Rapamycin upregulates glutamate transporter and IL-6 expression in astrocytes in a mouse model of Parkinson's disease. Cell Death Dis. *8*, e2611. https://doi.org/10.1038/cddis.2016.491.

34. Zhu, F., Fan, M., Xu, Z., Cai, Y., Chen, Y., Yu, S., and Zeng, L. (2018). [Neuroprotective effect of rapamycin against Parkinson's disease in mice]. Zhejiang Da Xue Xue Bao Yi Xue Ban *47*, 465–472.

35. Aniszewska, A., Bergström, J., Ingelsson, M., and Ekmark-Lewén, S. (2022). Modeling Parkinson's disease-related symptoms in alpha-synuclein overexpressing mice. Brain Behav. *12*, e2628. https://doi.org/10.1002/brb3.2628.

36. Brooks, S.P., and Dunnett, S.B. (2009). Tests to assess motor phenotype in mice: a user's guide. Nat. Rev. Neurosci. *10*, 519–529. https://doi.org/10.1038/nrn2652.

37. Bechman, N., and Maher, J. (2021). Lymphodepletion strategies to potentiate adoptive T-cell immunotherapy - what are we doing; where are we going? Expert Opin. Biol. Ther. *21*, 627–637. https://doi.org/10.1080/14712598.2021.1857361.

38. Elgueta, D., Chovar, O., Ugalde, V., Sanchez-Guajardo, V., Catenaccio, A., Court, F., and Pacheco, R. (2022). Analyzing the Parkinson's Disease Mouse Model Induced by Adeno-associated Viral Vectors Encoding Human alpha-Synuclein. J. Vis. Exp. *10*, 3791–63313.

39. Sanchez-Guajardo, V., Annibali, A., Jensen, P.H., and Romero-Ramos, M. (2013). alpha-Synuclein vaccination prevents the accumulation of parkinson disease-like pathologic inclusions in striatum in association with regulatory T cell recruitment in a rat model. J. Neuropathol. Exp. Neurol. *72*, 624–645. https://doi.org/10.1097/NEN.0b013e31829768d2.

40. Blat, D., Zigmond, E., Alteber, Z., Waks, T., and Eshhar, Z. (2014). Suppression of murine colitis and its associated cancer by carcinoembryonic antigen-specific regulatory T cells. Mol. Ther. *22*, 1018–1028. https://doi.org/10.1038/mt.2014.41.

41. Elgueta, D., Contreras, F., Prado, C., Montoya, A., Ugalde, V., Chovar, O., Villagra, R., Henríquez, C., Abellanas, M.A., Aymerich, M.S., et al. (2019). Dopamine Receptor D3 Expression Is Altered in CD4+ T-Cells From Parkinson's Disease Patients and Its Pharmacologic Inhibition Attenuates the Motor Impairment in a Mouse Model. Front. Immunol. *10*, 981. https://doi.org/10.3389/fimmu.2019.00981.

42. Ma, M., Paryani, F., Jakubiak, K., Xia, S., Antoku, S., Kannan, A., Lee, J., Madden, N., Senthil Kumar, S., Li, J., et al. (2025). The spatial landscape of glial pathology and T cell response in Parkinson's disease substantia nigra. Nat. Commun. *16*, 7146. https://doi.org/10.1038/s41467-025-62478-3.

43. Sivakumar, S.M., Safhi, M.M., Kannadasan, M., and Sukumaran, N. (2011). Vaccine adjuvants - Current status and prospects on controlled release adjuvancity. Saudi Pharm. J. *19*, 197–206. https://doi.org/10.1016/j.jsps.2011.06.003.

44. Frigault, M.J., and Maus, M.V. (2020). State of the art in CAR T cell therapy for CD19+ B cell malignancies. J. Clin. Investig. *130*, 1586–1594. https://doi.org/10.1172/JCI129208.

45. Iba, M., Kim, C., Sallin, M., Kwon, S., Verma, A., Overk, C., Rissman, R.A., Sen, R., Sen, J.M., and Masliah, E. (2020). Neuroinflammation is associated with infiltration of T cells in Lewy body disease and alpha-synuclein transgenic models. J. Neuroinflammation *17*, 214. https://doi.org/10.1186/s12974-020-01888-0.

46. Sommer, A., Fadler, T., Dorfmeister, E., Hoffmann, A.C., Xiang, W., Winner, B., and Prots, I. (2016). Infiltrating T lymphocytes reduce myeloid phagocytosis activity in synucleinopathy model. J. Neuroinflammation *13*, 174. https://doi.org/10.1186/s12974-016-0632-5.

47. Theodore, S., Cao, S., McLean, P.J., and Standaert, D.G. (2008). Targeted overexpression of human alpha-synuclein triggers microglial activation and an adaptive immune response in a mouse model of Parkinson disease. J. Neuropathol. Exp. Neurol. *67*, 1149–1158. https://doi.org/10.1097/NEN.0b013e31818e5e99.

48. Becker, M.I., Carrasco, I., Beltran, C., Torres, M., Jaureguiberry, B., and De Ioannes, A.E. (1998). Development of monoclonal antibodies to gizzerosine, a toxic component present in fish meal. Hybridoma *17*, 373–381. https://doi.org/10.1089/hyb.1998.17.373.

49. Köhler, G., and Milstein, C. (1975). Continuous cultures of fused cells secreting antibody of predefined specificity. Nature *256*, 495–497. https://doi.org/10.1038/256495a0.

50. Chmielewski, M., Hombach, A., Heuser, C., Adams, G.P., and Abken, H. (2004). T cell activation by antibody-like immunoreceptors: increase in affinity of the single-chain fragment domain above threshold does not increase T cell activation against antigen-positive target cells but decreases selectivity. J. Immunol. *173*, 7647–7653. https://doi.org/10.4049/jimmunol.173.12.7647.

51. Paxinos, G., and Franklin, K.B.J. (2001). The Mouse Brain in Stereotaxic Coordinates (Academic press).

52. Montoya, A., Elgueta, D., Campos, J., Chovar, O., Falcón, P., Matus, S., Alfaro, I., Bono, M.R., and Pacheco, R. (2019). Dopamine receptor D3 signalling in astrocytes promotes neuroinflammation. J. Neuroinflammation *16*, 258. https://doi.org/10.1186/s12974-019-1652-8.

53. Fleming, S.M., Salcedo, J., Fernagut, P.O., Rockenstein, E., Masliah, E., Levine, M.S., and Chesselet, M.F. (2004). Early and progressive sensorimotor anomalies in mice overexpressing wild-type human alpha-synuclein. J. Neurosci. *24*, 9434–9440. https://doi.org/10.1523/JNEUROSCI.3080-04.2004.

STAR★METHODS

KEY RESOURCES TABLE

REAGENT or RESOURCE	SOURCE	IDENTIFIER
Antibodies		
Anti-aggregated/fibril alpha-synuclein; MJFR-14-6-4-2	Abcam	Cat# ab209538; RRID: AB_2714215
Anti-phospho Ser129 alpha-synuclein	Biolegend	Cat# 825701; RRID: AB_2564891
Anti-phospho Ser129 alpha-synuclein	Invitrogen	Cat# PA5-77815; RRID: AB_2736285
Anti-monomeric alpha-synuclein; MJFR1	Abcam	Cat# ab138501; RRID: AB_2537217
Anti-nitrated alpha-synuclein; clone 1A11 mAb mouse IgG2a	this study	N/A
Anti-nitrated alpha-synuclein; clone 1A11 mAb human IgG4	this study	N/A
Anti-GFAP, clone 2E1.E9	Biolegend	Cat# 644702; RRID: AB_2294566
Anti-GFAP	Abcam	Cat# ab218309; RRID: AB_2909614
Anti-Iba1	Wako	Cat# 019-19741; RRID: AB_839504
Anti-Iba1	Abcam	Cat# ab178846; RRID: AB_2636859
Anti-arginase-1, clone 24H4L3	Invitrogen	Cat# 702730; RRID: 2734792
Anti-TH	Invitrogen	Cat# PA1-4679; RRID: 561880
Anti-TH	Millipore	Cat# AB152; RRID: AB_390204
Anti-DAT	Millipore	Cat# MAB369; RRID: AB_2190413
Anti-NeuN	Millipore	Cat# MAB377; RRID: AB_2298772
Anti-CD3 (LEAF) for mouse	Biolegend	Cat# 100371; RRID: AB_2800555
Anti-CD3 (LEAF) for human, clone OKT3	Biolegend	Cat# 317304; RRID: AB_571925
Anti-CD28 (LEAF) for mouse	Biolegend	Cat# 102131; RRID: AB_2810332
Anti-CD28 (LEAF) for human, clone CD28.2	Biolegend	Cat# 302914; RRID: AB_314316
Anti-IL-10 (LEAF); clone JES5-16E3	Biolegend	Cat# 505001; RRID: 315355
APC-Cy7-coupled anti human-CD69; clone FN50	Biolegend	Cat# 310914; RRID: AB_314849
PE-Cy7-coupled anti mouse-CD69; clone H1.2F3	Biolegend	Cat# 104512; RRID: AB_493564
Anti-CD4 depleting antibody; clone GK1.5, Rat isotype IgG2bκ	InVivoMAb	Cat# BE0003-1; RRID: AB_1107636
Bv421-coupled Anti-CD45.1, clone A20	Biolegend	Cat# 110722; RRID: AB_492866
AF488-coupled Anti-CD45.1, clone A20	Biolegend	Cat# 110718; RRID: AB_492862
APC-Cy7-coupled Anti-CD45.2, clone 104	Biolegend	Cat# 109824; RRID: AB_830789
PE-Cy7-coupled Anti-CD45.2, clone 104	Biolegend	Cat# 109830; RRID: AB
PerCP-coupled Anti-CD45.2, clone 104	Biolegend	Cat# 109826; RRID: AB_893349
AF546-coupled anti-rabbit	Thermo Fisher Scientific	Cat# A11010; RRID: AB_2534077
PE-conjugated anti-mouse IgG1 mAb, clone RMG1-1	Biolegend	Cat# 406608; RRID: AB_10551618
AF647-conjugated anti-sheep	Abcam	Cat# AB150179; RRID: AB_2884038
AF488-coupled anti-mouse	Invitrogen	Cat# A-21202; RRID: AB_141607
AF647-coupled anti-mouse	Invitrogen	Cat#A-21463; RRID: AB_2535869
AF488-coupled anti-rabbit	Invitrogen	Cat# A-21206; RRID: AB_2535792
AF555-coupled anti-rabbit	Thermo Fisher Scientific	Cat# A-21428; RRID: AB_2535849
AF546-coupled anti-rat	Thermo Fisher Scientific	Cat# A-11081; RRID: AB_2534125

(*Continued on next page*)

Continued		
REAGENT or RESOURCE	SOURCE	IDENTIFIER
Bacterial and virus strains		
AAV5-CAG-hαSyn	University of Iowa Viral Vector Core Facility	N/A
Chemicals, peptides, and recombinant proteins		
Concholepas concholepas hemocyanin (CCH); Immunocyanin	Biosonda Corp	N/A
Maleimide	Sigma-Aldrich	Cat# 129585
Glutaraldehyde (solution 25%)	Polyscience	Cat# 00376
Imject^{TM} Complete Freund's Adjuvant (CFA)	Thermo-Fisher Scientific	Cat# 77140
Imject^{TM} Incomplete Freund's Adjuvant (IFA)	Thermo-Fisher Scientific	Cat# 77145
Bovine Serum Albumin (BSA)	Rockland	Cat# BSA-50
mouse Interleukin-2 (IL-2)	Peprotech	Cat# SKU 212-12
mouse Interleukin-7 (IL-7)	Biolegend	Cat# 577804
Retinoic acid (RA)	Sigma Aldrich	Cat# R2625
Turbofect	Thermo Scientific	Cat# R-0531
Retronectin	Takara	Cat# T100B
Peptides derived from C-terminal of human αSyn (hαSyn$_{111-140}$) with postraslational modifications	GenScript/Biosynthesis	Table S1
Recombinant CEA (carcinoembrionary antigen)	Abcam	Cat# ab158095
Rapamycin	MedChemExpress	Cat# HY-10219
Fluoromount-G	Invitrogen	Cat# 00–4958-02
Hoechst 33342	Invitrogen	Cat# I35103C
Critical commercial assays		
CD4^{+} T cell negative selection kit, mouse	Miltenyi biotec	Cat# 130-104-454
CD4^{+} T cell Microbeads positive selection kit, human	Miltenyi biotec	Cat # 130-097-048
CellTrace Violet (CTV) Cell proliferation kit	Thermo Scientific	Cat# C34557
Mouse IL-10 ELISA kit	Biolegend	Cat# 431414
Cytometric bead assay (CBA) for human cytokines	Becton Dickinson	Cat# 560484
MACSibead T cell activation/expansion kit, mouse	Miltenyi biotec	Cat# 130-092-357
Fixation/Permeabilisation Foxp3 staining kit	Invitrogen	Cat# 00-5523-00
Zombie Aqua (ZAq) fixable viability kit	Biolegend	Cat# 423102
NucleoBond Xtra Maxi EF, Maxi kit for endotoxin-free plasmid DNA	Macherey-Nagel	Cat# 740424.10
Imject Maleimide Activated Blue Carrier Protein kit	Thermo Scientific	Cat# 77662
Rapid Antibody Isotyping kit	Pierce	Cat# 26178
Protein A-agarose	Millipore	Cat# 16-156
Quantitect Reverse Transcription Kit	Qiagen	Cat# 205311
Platinum 2*X* SuperFi DNA Polymerase PCR Mastermix	Thermo Fisher Scientific	Cat# 12358010
mouse-on-mouse HRP Polymer secondary antibody staining system	Biocare Medical	Cat# MM620H
ImmPACT DAB chromogenic substrate	Vector	Cat# SK-4105
RPMI medium	GIBCO	Cat# A10491-01
Fetal bovine serum (FBS)	GIBCO	Cat #A5256701

(*Continued on next page*)

Continued

REAGENT or RESOURCE	SOURCE	IDENTIFIER
Experimental models: Cell lines		
NS0/2 myeloma cells	Provided by Dr. C. Milstein from the MRC Laboratory of Molecular Biology, Cambridge, UK.	N/A
Phoenix ECO cells	ATCC	Cat # CRL-3214; RRID: CVCL_H717
NIH/3T3 cells	ATCC	Cat # CRL-1658; RRID: CVCL_0594
Experimental models: Organisms/strains		
C57BL/6N-Tg(Thy1-*SNCA*)15Mjff/J (*SNCA* mice)	The Jackson Laboratory	Strain #:017682; RRID:IMSR_JAX:017682
C57BL/6J (CD45.2)	The Jackson Laboratory	Strain #:000664 RRID:IMSR_JAX:000664
C57BL/6J (CD45.1)	The Jackson Laboratory	Strain #:002014 RRID:IMSR_JAX:002014
BALB/c	The Jackson Laboratory	Strain #:000651 RRID:IMSR_JAX:000651
Oligonucleotides		
caggcatcccagggtcaccatg (Outer primer for mouse IgG1)	Integrated DNA technologies	N/A
accatggagttagtttgggcagcagatc (Inner primer for mouse IgG1)	Integrated DNA technologies	N/A
caagaagcacacgactgaggcac (Outer primer for mouse Igκ light chain)	Integrated DNA technologies	N/A
ggcacctccagatgttaactgctcactg (Inner primer for mouse Igκ light chain)	Integrated DNA technologies	N/A
Recombinant DNA		
pBullet vector CAR cassette	Provided by Dr. Hinrich Abken	N/A
pBullet-CAR1A11 VH-VL-Foxp3 vector	This study	N/A
pBullet-CAR1A11 VL-VH-Foxp3 vector	This study	N/A
pBullet-CARCEA-Foxp3 vector	This study	N/A
pCL-Eco	Addgene	Cat# 12371; RRID: Addgene_12371
pLV-hCAR1A11 VH-VL-hFOXP3-EGFP	VectorBuilder	N/A
pLV-EGFP	VectorBuilder	N/A
Software and algorithms		
ImageJ	NIH	RRID:SCR_002285
Imaris	Oxford instruments	V 10.2.0; RRID: SCR_007370
Graphpad prism	GraphPad Software	V 10.6.1; RRID: SCR_002798
FACSDiva	Becton Dickinson	RRID:SCR_1456
FlowJo	Tree Star	V9/10; RRID:SCR_008520
Other		
Stereotaxic frame	Stoelting, Wood Dale, IL, USA	N/A

EXPERIMENTAL MODEL AND STUDY PARTICIPANT DETAILS

Animals

Female C57BL/6J wild-type (WT) homozygous for the CD45.2 allele were crossed with male C57BL/6J *SNCA* (*SNCA*) mice heterozygous for the Thy1-α-synuclein transgene (encoding human a-synuclein under the mouse Thy1 promoter) which is integrated on mouse Chromosome 11 (40456787–40495044) and results in a 38.4 kb deletion. Only male WT or heterozygous *SNCA* mice were used for experiments in this study, as female mice did not present a significant motor impairment (unpublished data). Male WT ($Cd45.2^{+/+}$) mice and transgenic *SNCA* ($Cd45.2^{+/+}$) mice of different ages (see the specific ages in figure legend) were used. When indicated in figure legends, WT ($Cd45.1^{+/+}$) mice were used as donors of lymphocytes to generate CAR-Treg cells. Ten-week-old male C57BL/6J WT mice were used in the preclinical model of neurodegeneration induced by the stereotaxic delivery

of AAV-hαSyn. Eight-week-old female wild-type BALB/c mice were used for mAb production. Animals were housed under specific pathogen-free conditions in plastic homecages in a temperature-controlled room, under a 12 h light/dark cycle. The environment was enriched with sterile cardboard cones. All animals had *Ad libitum* access to standard rodent food pellets and drinking water. Experimental mice were daily monitored and received a health score (from 0 to 12) based on body weight loss (from 0 to 3), general healthy aspect (from 0 to 3), spontaneous behavior (from 0 to 3) and stool consistency (from 0 to 3). When the health score was $\geq$7, mice were euthanised with CO_2 overdose. When the health score was lower than 7 and difficulty eating or drinking was noticed, food and hydration were provided in the form of wet jellies. All procedures conducted in animals and housing complied with the recommendations in the 8th edition of the Guide for the Care and Use of Laboratory Animals and with the United States Public Health Service Policy. The IACUC of Fundación Ciencia & Vida approved the protocols for the AAV-hαSyn model (Permit Number: P035/2022) and the *SNCA* model (Permit Number: P043/2022).

Human subjects

Serum samples were obtained from patients diagnosed with Parkinson's disease according to the UK Parkinson's Disease Society Brain Bank criteria and from age-matched healthy controls. Clinical evaluation included UPDRS, Hoehn and Yahr staging, Schwab and England scale, and MoCA assessment. For demographic information see Table S2. Exclusion criteria included acute or chronic inflammatory diseases, autoimmune disorders, cancer, hepatic or renal dysfunction, and metabolic disorders. The study performed with human individuals conforms to the principles outlined in the Declaration of Helsinki, the study protocol was approved by the local Ethics Committee of the Hospital del Salvador (Permit Number: 28E/2014), Santiago (Chile), and all the participants signed a written informed consent before enrollment.

METHOD DETAILS

Generation of monoclonal antibodies specific to nitrated α-synuclein

The 3NY-hαSyn peptide was covalently conjugated to the highly immunogenic protein *Concholepas concholepas* hemocyanin (CCH) and used as the immunogen in BALB/c mice, as described before.[48] Maleimide and glutaraldehyde were used as two alternative linkers for the generation of CCH-3NY-hαSyn conjugates. Mice were immunised with CCH-3NY-hαSyn mixed with CFA in the first immunisation and mixed with IFA in the second and third immunisation (Figure S2). PBS was used instead CCH-3NY-hαSyn in a control mouse. After the second (Figure S3) and the third (Figure S4) immunisation, the presence of specific antibodies for 3NY-hαSyn or unmodified hαSyn in the serum of mice was monitored by ELISA. The results show high immunoreactivity for 3NY-hαSyn but not for unmodified hαSyn in the serum of mice immunised with CCH-3NY-hαSyn generated with maleimide as a linker (Figures S3 and S4). Despite immunisation with CCH-3NY-hαSyn using glutaraldehyde as linker also resulted in a significant generation of antibodies with a selective specificity for 3NY-hαSyn but not unmodified hαSyn in the serum, the immunoreactivity of these serums for 3NY-hαSyn was lower than serums obtained from mice immunised with CCH-3NY-hαSyn generated with maleimide (Figures S3 and S4). We proceeded to use splenocytes from mice immunised with maleimide-CCH-3NY-hαSyn for further production of mAbs. Splenocytes from these mice were fused with the myeloma cell line NS0/2 to generate hybridomas, which were subsequently cloned by limiting dilutions as described before.[49] The supernatant of hybridomas obtained was analyzed for their immunoreactivity to 3NY-hαSyn and unmodified hαSyn by ELISA (Figure S5A). After the supernatant screening, we obtained two hybridomas producing mAbs with selective reactivity for 3NY-hαSyn but not for unmodified αSyn: the clone 1A11 with IgG1κ isotype and the clone 8E7 with IgAκ isotype (Figure S5B). To further analyze the specificity of these mAbs, we also tested the immunoreactivity of the hybridomas' supernatant to 31-mer peptides containing the same aminoacidic composition of the 3NY-hαSyn peptide, including three nitrated tyrosines but scrambled sequence (3NY-scrambled; Table S1). Importantly, whereas the 1A11 mAb displayed specific immunoreactivity to 3NY-hαSyn but not to 3NY-scrambled or unmodified hαSyn peptides, the 8E7 mab presented considerable immunoreactivity for 3NY-hαSyn and 3NY-scrambled (Figure S6). These results indicate that the 1A11 mAb was the only antibody specific to 3NY-hαSyn. Conjugation of 3NYαSyn peptide to native CCH. Native CCH (5 mg; Biosonda Corporation, Chile) was dissolved in 1 mL borate buffer 0.1 M (at pH 10), while 3NY-hαSyn peptide (5 mg; synthesized by GenScript) was dissolved in 325 μL of 0.1 M borate buffer (at pH 10) and then both solutions were mixed. Of note, a cysteine was included at the N-terminal extreme of the 3NY-hαSyn peptide to allow the conjugation with CCH. Afterward, 0.5 mL of 0.3% glutaraldehyde (Polysciences, USA) in borate buffer 0.1 M (at pH 10) was slowly added and incubated for 2 h in darkness. The reaction was stopped with 250 mL of glycine 1M and left in the dark for 30 min, finally stored at 4°C. For the conjugation of the peptide 3NYαSyn to CCH using maleimide as the linker, the protocol suggested by the manufacturer for using the Imject Maleimide Activated Blue Carrier Protein kit was followed. Immunisation. Three groups of 12-weeks old female BALB/c mice were i.p. immunised; some of them with the glutaraldehyde 3NY-hαSyn-CCH conjugate (n = 3), another group with the maleimide 3NY-hαSyn-CCH conjugate (n = 3) and a control group injected with PBS instead immunogen (n = 1). In all groups, 250 μg of immunogen was administered per mouse per immunisation in a volume of 150 μL of immunogen and 150 μL of CFA (Pierce) for the first immunisation or IFA (Pierce) for the second and third immunisations. ELISA for antibody detection. 96-well plates (Pierce) were activated with 100 μL of unmodified or nitrated hαSyn (10 μg/mL) peptides (Table S1) overnight at 4°C. Afterward, plates were blocked with 1% casein in PBS (100 μL per well) for 1 h at 37°C. After washing, plates were incubated with the immune serum in indicated dilutions (in PBS) or with hybridoma supernatants using 100 μL per well and incubated for 3 h at 37°C. Plates were washed three times with PBS containing 0.02% tween 20. Afterward, plates were incubated with alkaline

phosphatase-conjugated mouse anti-Ig H + L (in a dilution of 1:2000; Thermo Scientific) or HRP-conjugated mouse anti-Ig H + L (in a dilution of 1:1000; Thermo Scientific) in PBS containing 1% casein and incubated for 30 min at 37°C. Plates were washed three times with 0.02% tween 20 in PBS and then incubated with 100 μL of substrate. In the case of alkaline phosphatase, we used *p*-nitrophenyl phosphate (PNPP; 10 mg/mL) in alkaline phosphatase buffer (Tris 100 mM, NaCl 100 mM and $MgCl_2$ 50 mM) for 30 min at 37°C, and the absorbance was read at 405 nm. In the case of HRP, we used TMB (1 mg/mL) containing H_2O_2 (dilution 1:5000) for the time necessary until the blue color was evident (approximately 10–15 min). The reaction was stopped by adding H_2SO_4 2M (50 μL/well) and the absorbance was read at 450 nm. Kohler fusion. Once the donor mouse was selected, the spleen was removed and homogenized and left in the same medium containing non-secreting murine myeloma cells (NS0/2 cells; 25×10^6 cells). This mixture was washed with high-glucose DMEM medium (HyClone, USA) and then resuspended in 0.5 mL of polyethylene glycol (PEG, Merck, Germany), which was added dropwise under agitation for 1 min. Afterward, this mixture was agitated for an additional 90 s and then, 1 mL of high glucose DMEM medium was added for 30 s. Consecutively 3 mL of high glucose DMEM medium was added for the next 30 s, and then 16 mL of medium was added for 1 min, all the time under agitation. The cell-fusion mixture was left at room temperature for 5 min and then 20 mL of additional medium was added. Cells were centrifuged at 1000 rpm for 10 min at room temperature. The pellet of cells was resuspended in HAT medium (containing hypoxanthine, aminopterin, thymidine, antibiotics, pyruvate, L-glutamine (all from Gibco, USA), and 10% FBS (HyClone) in high glucose DMEM medium) and plated onto 96-well plates flat bottom at 100 μL per well. Selection of hybridoma clones. After the generation of hybridomas, 584 potential positive clones were selected in the HAT medium and analyzed for the production of anti-3NY-hαSyn mAb. ELISA for 3NY-hαSyn, 3NY-scrambled and control-hαSyn peptides (Figure S6) were assessed to select clones positive for anti-3NY-hαSyn mAb production. Positive reactivity was considered when hybridoma supernatants displayed OD higher than 0.5. Using this selection criterion, 14 positive clones were obtained. These clones were expanded, and the antibody production was once again tested by ELISA (using the same OD criteria). Among the 14 expanded clones, three of them produced supernatants with reactivity to 3NY-hαSyn displaying OD higher than 0.5 and negative reactivity for control-hαSyn peptide. These three clones were re-cloned by serial dilution in 96-well plates in such a way that the number of cells in each row was reduced by half. Finally, the obtained clones were tested by ELISA and clones 1A11 and 8E7 displayed immunoreactivity to 3NY-hαSyn but not to unmodified hαSyn (Figure S5B). Nevertheless, clone 8E7 was also immunoreactive to 3NY-scrambled (Figure S6). Isotype of mAb secreted by hybridomas was detected using the Rapid Antibody Isotyping kit.

Determining 1A11 mAb immunoreactivity in human serum by dot-blot

Serum samples were diluted in PBS up to obtain protein concentration of 10 mg/mL. To deplete immunoglobulins, 900 μL of serum were incubated with 100 μL of protein A-agarose at 4°C in agitation for 2 h. Afterward, samples were centrifugated, and the supernatant was store for further analysis. 1 μL of the immunoglobulin-depleted serum was diluted with 9 μL PBS and then analyzed by dot-blot. To this end, the 10 μL sample was incubated in a polyvinylidene difluoride membrane (PVDF, Thermo Scientific, Rockford, IL, USA). After the sample was evaporated, the membrane was incubated with blocking buffer (5% milk in PBS containing 0.05% tween 20) at room temperature for 1h. PVDF membranes were then incubated with the 1A11 mAb (0.1 μg/mL), followed by HRP-conjugated goat anti-mouse IgG Ab (1:7500; Rockland, Gilbertsville, PA, USA). As a control, samples were incubated with the anti-hαSyn monomeric mAb (MJFR1 rabbit mAb) followed by HRP-conjugated goat anti-rabbit IgG secondary Ab. Immunodetection was carried out with Fast western kit SuperSignal chemiluminescent substrate (Thermo Scientific, Rockford, IL, USA).

Western blot analysis

Brain and intestine homogenates were dissolved in RIPA lysis buffer, which was composed of 50 mM Tris–HCl (pH 7.4), 150 mM NaCl, 1% (v/v) NP-40 or Triton X-100, 0.5% (w/v) sodium deoxycholate, and 0.1% (w/v) SDS, and was supplemented with protease and phosphatase inhibitor cocktails prior to use. Protein concentration was determined using the Bradford assay. For analysis of human serum samples, protein concentration was determined directly using the Bradford assay. Brain or gut lysates from mice, or human serum samples were mixed with 5*X* SDS-PAGE loading buffer and incubated at 60°C for 5 min. The 5*X* SDS–PAGE loading buffer consisted of 250 mM Tris–HCl (pH 6.8), 10% (w/v) SDS, 50% (v/v) glycerol, 0.05% (w/v) bromophenol blue, and 500 mM dithiothreitol as a reducing agent. Indicated amounts of protein extract (10, 20, 50, and 100 μg) were separated on SDS–PAGE gels and transferred onto PVDF membranes (Amersham Hybond P, 0.45 μm). Membranes were blocked for 1 h in 5% milk prepared in TBS–Tween and incubated overnight at 4°C with the primary antibody (human 1A11 mAb diluted 1:1000; mouse 1A11 mAb diluted 1:2500; rabbit anti-α-Syn aggregated mAb,MJFR-14-6-4-2 diluted 1:2500; rabbit anti-α-Syn monomeric mAb, MJFR1 diluted 1:5000; rabbit anti-pSer129-αSyn pAb from Biolegend diluted 1:2500) in blocking solution. After washing three times with TBS–Tween, membranes were incubated for 1 h at room temperature with the secondary antibody (HRP-coupled anti-human 1:5000; HRP-conjugated anti-mouse 1:5000; HRP-conjugated anti-Rabbit 1:5000), washed, and developed using SuperSignal West Dura Extended Duration Substrate.

Chimeric antigen receptor engineering and production

RNA was isolated from a hybridoma (clone 1A11) cell line using TriReagent (Sigma-Aldrich) following the manufacturer's instructions. After RNA quantification, it was retro-transcribed to cDNA using Quantitect Reverse Transcription Kit according to the manufacturer's protocol. A Poly-C tail was added at the 3′ end of cDNA using Terminal deoxynucleotidyl transferase (New England Biolabs). The variable region of the antibodies was amplified through a 2-step (nested) PCR using Platinum 2*X* SuperFi DNA Polymerase PCR

Mastermix accordingly to the manufacturer's instructions. This nested PCR was done for both the light (VL) and heavy chain (VH) of the antibody. For VH amplification, an outer primer (caggcatcccagggtcaccatg) and an inner primer (accatggagttagtttgggcagcagatc) specific for the mouse IgG1 heavy chain were used. For VL amplification, an outer primer (caagaagcacacgactgaggcac) and an inner primer (ggcacctccagatgttaactgctcactg) specific for the mouse Igκ light chain were used. The amplification was performed as follows: 94°C for 2 min, followed by 20 cycles of 94°C for 30 s, 56°C for 30 s, and 68°C for 90 s. After the last cycle, samples were heated at 68°C for 10 min and then stored at 4°C. After amplification, PCR products were separated in 1.2% agarose gel and bands were cut for further purification and sequentiation. The DNA sequences encoding for VH and VL regions from 1A11 hybridoma were separated by a spacer (Figure S8) to generate a single chain variable fragment (scFv; the antigen-recognizing site of the CAR). The scFv was synthesized in both versions 5′VH-VL3′ and 5′VL-VH3'. Each scFv was inserted into a pBullet vector CAR cassette including an extracellular IgG1 hinge domain and intracellular CD28 and CD3z moieties responsible for costimulatory and activation signals, respectively, as described before.[50] The *Foxp3* gene was inserted into the construct at the 3′ side of the CAR-1A11 gene and separated by a 2A peptide, as described before.[26] All these genes were under the control of a strong promoter (cytomegalovirus; CMV) located at 5'. Similar to the generation of CAR-1A11/Foxp3 vectors, to obtain a control CAR with an irrelevant specificity for Parkinson's disease, a CAR with the scFv obtained from CEA-specific hybridomas was used.[40] Using the DNA sequences encoding VH and VL regions from the 1A11 hybridoma, recombinant versions of the 1A11 mAb were generated with the mouse IgG2aκ isotype and with the human IgG4κ isotype using the TurboCHO™ antibody expression technology by GenScript services.

Transduction of T-cells with CARs and adoptive transfer

The different pBullet vectors encoding CAR-1A11-VL-VH/Foxp3, CAR-1A11-VH-VL/Foxp3 or CAR-CEA/Foxp3 were amplified in E. Coli followed by bacterial lysis and plasmid purification with NucleoBond Xtra Maxi EF (Macherey-Nagel). To generate cells producing CAR-1A11/Foxp3 or CAR-CEA/Foxp3 retroviral particles in the supernatant, ECO Phoenix™ retroviral packaging cells (which express endogenous gag, pol and envelope proteins) were transfected with 4 μg CAR-1A11/Foxp3 or CAR-CEA/Foxp3 vectors in addition of 2.5 μg pCLEco vector in Turbofect/OPTIMEM medium. 24h later, the medium was replaced with RPMI and retroviral particles were harvested at 48 and 72 h post-transfection. To generate Treg expressing CAR-1A11 or CAR-CEA, $CD3^+$ $CD4^+$ T-cells were isolated from the spleen of wild-type C57BL/6 (CD45.1) mice by using the $CD4^+$ T cell negative selection kit (Miltenyi Biotec) and then activated (10^6 cells per well) with αCD3/αCD28-coated MACSibeads (T cell activation/expansion kit, MACSiabead-to-cell ratio 2:1; Miltenyi Biotec) in the presence of IL-2 (1 μg/mL; Peprotech) and IL-7 (1 ng/mL; Biolegend) in 24-well plates for 12 h. Concomitantly, 24-well plates were coated with retronectin (12 μg/mL; Takara) at 4°C for 12h and then with retroviral particles obtained from the supernatant of ECO Phoenix™ retroviral packaging cells at room temperature for 30 min. Afterward the supernatant was removed, and activated $CD4^+$ T-cells were added on plates coated with retronectin and the first layer of viral particles and spinoculated at 1200 g and 37°C for 90 min. Cells were incubated for 24h at 37°C and then again spinoculated over a second layer of viral particles in the presence of fresh IL-2, IL-7 and MACSibeads. After a final incubation at 37°C for 24 h, MACSibeads were removed using a magnet, and $CD4^+$ $IgG1^+$ ZAq^- cells were isolated by cell sorting and i.v. injected into wild-type C57BL/6 recipient mice.

Determination of the CAR1A11 affinity

NIH/3T3 cells were transduced with the CAR1A11 and then incubated with indicated concentrations of the 5-FAM-coupled peptides derived from the C-terminal of the hαSyn (hα$Syn_{111-140}$; Table S1) for 10 min. Cells were washed three times with PBS and resuspended in PBS at 10^6 cells/mL. Then, the fluorescence associated with 5-FAM was quantified in the population of live cells expressing the CAR1A11 ($IgG1^+$ ZAq^-) by flow cytometry.

In vitro activation assay for CAR-Treg

Spleen $CD3^+$ $CD4^+$ T-cells were transduced with CAR-1A11/Foxp3 vectors and MACSibeads were removed. CAR-Treg were then activated with plate coated CD3 (1 μg/mL) and soluble anti-CD28 (2 μg/mL) for 48 h. Concomitantly, 96-well ELISA plates were incubated with hα$Syn_{111-140}$-derived peptides or CEA (1 μg/mL), or anti-CD3 (1 μg/mL) plus anti-CD28 (2 μg/mL). Afterward, activated CAR-Treg (5×10^4 cells/well) were incubated with the different antigens for 24 h and the surface CD69, CTLA4, CD25, PD1 and GITR expression was determined in live $CD4^+$ T-cells expressing the CAR1A11 ($IgG1^+$ ZAq^- population) by flow cytometry, while IL-10 production was assessed in the culture supernatant by ELISA (Biolegend).

In vitro suppression assay for CAR-Treg

TCRβ^+ $CD25^-$ T cells were isolated by cell sorting from the spleen of 20-week-old *SNCA* mice, labeled with 5 μM CellTrace Violet (CTV), and co-cultured (10^5 cells/well) with activated CAR-Treg cells (3×10^3 cells/well) in 96-well plates in the presence of plate-coated 133,136-NY-hαSyn peptide (1 μg/mL), plate-coated anti-CD3 (1 μg/mL), and soluble anti-CD28 (2 μg/mL). Prior to co-culture, CAR-Treg cells were activated with plate-coated anti-CD3 (1 μg/mL) and soluble anti-CD28 (2 μg/mL) in the presence of IL-2 (300 U/mL), IL-7 (1 ng/mL), and RA (500 ng/mL) for 72 h. Co-cultures were then incubated for a further 72 h in the presence or absence of a neutralising anti-IL-10 mAb (1 μg/mL; clone JES5-16E3), and naive T cell proliferation was assessed by measuring CTV dilution within the TCRβ^+ $CD8^+$ ZAq^- population by flow cytometry. IL-10 levels in culture supernatants were quantified by ELISA.

Generation and *in vitro* activation assays of human CAR-Treg

A lentiviral vector encoding the human version of the CAR1A11 was synthesized by VectorBuilder services, including a strong promoter (promoter of the human eukariotic translation elongation factor 1α; EF1α) followed by the human CAR1A11 built with a transit peptide, the CAR1A11 in VH-VL conformation, CD8-hinge region, CD8-transmembrane region and the intracellular signaling domains from CD28 and CD3zeta. Dowstream in the vector sequence was encoded the self-cleaving 2A peptide from porcine teschovirus-1 (P2A) followed by the hFOXP3 sequence [NM_014009.4]. At 3′ was then encoded the sequence of the human cytomegalovirus immediate-early enhancer/promoter (CMV) followed by the gene of enhanced green fluorescent protein (EGFP). Detailed map of this vector (pLV-hCAR1A11 VH-VL-hFOXP3-EGFP) might be found in the Figure S12A. A control vector encoding only the EGFP was also generated (pLV-EGFP; VectorBuilder). Human peripheral blood mononuclear cells (PBMCs) were obtained immediately after the extraction of heparinized blood from HC using Ficoll-PaqueTM Plus (Ge Healthcare). $CD4^+$ T-cells were purified from PBMCs by positive selection using the CD4 Microbeads kit, and then were activated in 96-well plates caoted with LEAF purified anti-human CD3 mAb (5 μg/mL) and LEAF purified anti-human CD28 mAb (2 μg/mL) in RMPI containing 5% FBS at 37°C and 5% CO_2 for 48 h. Activated $CD4^+$ T-cells were then transduced with lentiviral vectors pLV-hCAR1A11 VH-VL-hFOXP3-EGFP or pLV-EGFP using polybrene (8 μg/mL) in the presence of IL-2 (1000 IU/mL) and retinoic acid (RA; 100 nM) for 5 days. Cells were washed with fresh medium and then incubated only with IL-2 and RA for additional 2 days. $CD4^+$ T-cells were then incubated in 96-well plates coated with LEAF purified anti-human CD3 mAb and soluble LEAF purified anti-human CD28 mAb, or 96-well plates coated with 3NY-hαSyn (1 μg/mL) in RPMI medium for 24 h and the extent of T cell activation was assessed by the surface CD69 expression in the GFP^+ population by flow cytometry. IL-10 production was determined in the culture supernatant by using the cytometric bead array (CBA) kit for human cytokines.

Mouse models and treatments

SNCA model. CAR-Treg cells were administered intravenously starting at pre-symptomatic stages (week 10 of age) and mice were monitored longitudinally. Experimental groups receiving different treatments were made by selecting *SNCA* male mice randomly. Control groups received polyclonal Treg (a single i.v. injection of 10^5 cell/mouse), or rapamycin (7.5 mg/Kg) i.p. three times a week for 10 weeks. Non-treated WT mice were also used as controls. Stereotaxic surgery. Adenovirus associated vectors (AAV5) encoding for hαSyn under the control of the chicken beta actin gene (CAG) promoter was obtained from the University of Iowa Viral Vector Core Facility. The final vector concentrations was 10^{13} vg/mL for AAV5-CAG-αSyn. The surgeries were conducted as described before.[38] Briefly, once anesthetized, mouse heads were fixed in a stereotaxic frame (Stoelting, Wood Dale, IL, USA) and 1 μL of AAV5-CAG-αSyn was administered into the right Substantia Nigra using the following coordinates: anteroposterior 2.8 mm respect to bregma, mediolateral 1.4 mm respect to the medial line and dorsoventral 7.2 mm respect to dura.[51] The injections were performed at a rate of 0.2 μL/30 s using a 10 μL Hamilton syringe coupled to a glass capillary. The needle was left in position for 5 min after the delivery of viral vectors and then withdrawn slowly. The animals were sutured with Silkam thread (B Braun) and received analgesia for the following three days (carprofen 5 mg/Kg s.c. every 12 h).

Transient depletion of $CD4^+$ T-cells

Animals received three i.p. injections of a rat anti-mouse CD4 Ab (αCD4; isotype IgG2bκ; InVivoMAb, clone GK1.5) 100 μg each, separated for 24 h. $CD4^+$ T cell depletion was confirmed by flow cytometry in the live (ZAq^-) $TCR\beta^+$ population from peripheral blood samples 7 and 14 days after the last injection.

Tracking CAR-Treg distribution *in vivo*

CAR-Treg cells, generated by transducing $CD3^+$ $CD4^+$ T cells isolated from the spleen of $CD45.1^+/^+$ mice using a $CD4^+$ T cell negative selection kit (Miltenyi Biotec), were intravenously injected (10^6 cells per mouse) into 10-week-old congenic $CD45.2^+/^+$ *SNCA* mice. Mice were sacrificed 1, 3, or 10 weeks later, and the relative composition of donor ($CD45.1^+$) and endogenous ($CD45.2^+$) $CD4^+$ T cells isolated from different tissues was analyzed by flow cytometry, including blood, spleen, CLN, MLN, colonic lamina propria (cLP), small-intestinal lamina propria, spinal cord, and brain. Tissues were obtained as indicated below. Immunostaining of fluorophore-coupled anti-CD45.1 (clone A20, coupled to AF488 or Bv421) combined with fluorophore-coupled anti-CD45.2 (clone 104, coupled to PE-Cy7, APC-Cy7 or PerCP) antibodies was analyzed in the $TCR\beta^+$ $CD4^+$ ZAq^- population by flow cytometry.

Flow cytometry

For surface markers immunostaining, cells were incubated with Zombie aqua (ZAq) fixable viability kit (Biolegend) and immunostained with fluorophore-conjugated monoclonal antibodies (mAbs) for 15 min at room temperatute. For intracellular cytokine staining, cells were re-stimulated with the indicated peptide (1 μg/mL; Table S1) and ionomycin (1 mg/mL; Sigma) in the presence of brefeldin A (5 μg/mL; Biolegend) for 3 h. After staining surface markers, cells were fixed and permeabilised using the Fixation/Permeabilisation Foxp3 staining kit (Invitrogen), according to the manufacturer's instructions. Permeabilised cells were incubated with fluorophore-conjugated mAbs to intracellular markers for 15 min at room temperature. All flow cytometry analyses were performed using a FACSCanto II flow cytometer, and collected data were analyzed using FACSDiva (BD Biosciences) and FlowJo software (Tree Star, Ashlan, OR, USA).

Tissue processing

Animals were anesthetized with an overdose of 5% isoflurane (Sigma-Aldrich) and sacrificed by transcardial perfusion with PBS. For histological techniques, brains were removed, fixed with 4% paraformaldehyde (Merck, Darmstadt, Germany) in 0.125 M phosphate buffered saline (PBS, pH 7.4), and then cryoprotected for 48 h in 20% glycerine and 2% DMSO in PBS. Blood samples (∼1 mL) were collected by cardiac puncture and processed for erythrocyte lysis using ACK buffer through sequential centrifugation and incubation steps, yielding cell suspensions ready for staining. Spleens were mechanically dissociated in ACK buffer using a 29G syringe and glass slides, followed by centrifugation, resuspension in PBS, cell counting, and adjustment to a final concentration of 10^7 cells/mL. Cervical and mesenteric lymph nodes were mechanically dissociated in PBS, centrifuged, and resuspended to obtain single-cell suspensions for staining. For the isolation of lamina propria cells from the colon and small intestine, intestinal tissues were excised, cleaned of fat and luminal contents, opened longitudinally, and cut into small fragments, which were subjected to sequential predigestion steps in EDTA-containing buffer at 37°C with continuous rotation, followed by washing in HBSS without Ca^{2+}/Mg^{2+}. Tissues were subsequently enzymatically digested in pre-warmed digestion buffer containing DNase I, collagenase IV, and liberase using a gentleMACS dissociator, and the resulting cell suspensions were filtered, centrifuged, and enriched by Percoll density gradient centrifugation, with cells recovered from the interphase and washed prior to staining. Brain and spinal cord tissues were minced and enzymatically digested in HBSS containing collagenase IV and DNase I at 37°C under agitation, followed by filtration, centrifugation, and Percoll gradient separation to isolate mononuclear cells, which were collected from the interphase, washed, and prepared for downstream staining. All buffers and solutions were prepared under sterile conditions as specified, and cells from all tissues were processed immediately for subsequent immunostaining analyses.

Immunohistochemical and immunofluorescent analysis

Blinded immunohistochemical and immunofluorescence analysis evaluation of TH, NeuN, Iba1, GFAP, DAT, pSer129-hαSyn and NhαSyn (mAb 1A11) in the SN and striatum was performed. RP was the only person who knows the identity of mice evaluated. To carry out immunohistochemical and immunofluorescent analysis, free floating sections (40 μm thick in the case of the AAV-hαSyn model or 20 μm thick in the case of the *SNCA* model) were processed at the same time in each experiment. For immunohistochemical analysis, sections were washed with PBS and endogenous peroxidase activity was inactivated by 30 min incubation with 0.03% H_2O_2 in methanol (Sigma-Aldrich). After washing three times with PBS, the tissue was incubated for 40 min with blocking solution [4% goat serum, 0.05% Triton X-100 (Sigma-Aldrich) and 4% BSA (Merck, Darmstadt, Germany) in PBS], and exposed overnight to the primary antibodies diluted in blocking solution at room temperature. The primary antibodies used were: rabbit anti-tyrosine hydroxylase (TH) pAb (1:1000; Millipore, Temecula, CA, USA), rabbit anti-Iba1 antibody (1:1000; Abcam, Cambridge, United Kingdom), and rabbit anti-GFAP antibody (1:500; Abcam, Cambridge, United Kingdom). For colorimetric immunohistochemistry, antibody binding was detected by incubating sections with biotinylated goat anti-rabbit secondary pAb (1:500; Jackson ImmunoResearch Laboratories, West Gore, PA, USA) in blocking solution for 2 h at room temperature. The biotinylated antibody was detected by incubation with peroxidase-conjugated avidin (1:5000; Sigma-Aldrich) for 90 min at room temperature followed by incubation with 0.05% diaminobenzidine (Sigma-Aldrich) in 0.03% H_2O_2/Trizma-HCl buffer (pH 7.6). Sections were mounted on glass slides in a 0.2% solution of gelatine in 0.05 M Tris (pH 7.6) (Sigma-Aldrich). The mean number of TH^+ neurons from six SN *pars compacta* (SNpc) sections (separated by 120 μm in between each other) per mouse was quantified under light microscopy at a magnification of 200*X*, and the total area of SNpc was calculated using ImageJ software (National Institutes of Health, Bethesda, MD). Density of dopaminergic neurons was expressed as the number of TH^+ neurons per area (mm^2) in the SNpc. To evaluate the extent of astrogliosis, the mean of GFAP-associated immunoreactivity was analyzed in areas of interest (660 μm × 877 μm) in five striatum sections per mouse and quantified as the integrated density using the ImageJ software as described.[52] To determine the extent of activated microglia, the mean number of $Iba1^{high}$ reactive microglia displaying ameboid shape was quantified in areas of interest of 660 μm × 877 μm in five striatum sections per mouse as described before.[52] In the case of immunohistochemical analysis of 1A11 immunoreactivity in colonic sections, tissue was embedded in paraffin and cut in 3 μm sections. Antigen recovery was conducted by heating tissue at 95°C for 40 min in Decloacking Chamber NxGen. Incubation with the mouse recombinant 1A11 IgG1 primary mAb (0.5 μg/mL), was followed by incubation with mouse-on-mouse HRP Polymer secondary antibody staining system and ImmPACT DAB chromogenic substrate.

For immunofluorescence analysis, brain slices were washed three times with PBS, and then incubated with blocking solution [0.3% Triton X-100, 0.05% tween 20 (both from Sigma-Aldrich) and 5% BSA (Merck, Darmstadt, Germany) in PBS] for 40 min, and exposed overnight to the primary antibodies (rabbit anti-TH pAb diluted 1:500, from Millipore; sheep anti-TH pAb diluted 1:100, from Invitrogen; mouse anti-NeuN mAb clone A60 diluted 1:100, from Millipore; rabbit anti-Arginase 1 mAb, clone 24H4L3 diluted 1:100, from Invitrogen; rabbit anti-pSer129-αSyn pAb diluted 1:100, from Invitrogen; mouse anti-GFAP mAb clone 2E1.E9 diluted 1:250, from Biolegend; rabbit anti-Iba1 pAb diluted 1:500, from wako; rat anti-DAT pAb, diluted 1:250, from Millipore; mouse 1A11 mAb used at 4 μg/mL, synthesized by GenScript) diluted in blocking solution at 4°C in constant agitation. When mouse antibodies were used, the blocking solution also included a rat anti-mouse CD16/32 mAb (1:400; clone 93, eBioscience), which was used to block the FcγRII (CD32) and FcγRIII (CD16). Brain slices were washed three times with PBS and then incubated with secondary antibody (AF546-coupled anti-rabbit from Thermo Fisher Scientific; PE-conjugated anti-mouse IgG1 mAb, clone RMG1-1 from Biolegend; AF647-conjugated anti-sheep from Abcam; AF488-coupled anti-mouse from Invitrogen; AF647-coupled anti-mouse from Invitrogen; AF488-coupled anti-rabbit from Invitrogen; AF555-coupled anti-rabbit from Thermo Fisher Scientific; AF546-coupled anti-rat from

Thermo Fisher Scientific) in constant agitation at room temperature with agitation for 2 h. Brain slices were washed three times with PBS and covered in Fluoromount-G with Hoechst 33342. All images were acquired using a Leica DMi8 epifluorescence microscope. For DAT staining, stitched images covering the entire brain section were analyzed using ImageJ software, and integrated density (IntDen) values were plotted. For the analysis of GFAP and Arg1 colocalisation, the JaCoP plugin in ImageJ was used, and Manders' coefficient M2 values were quantified. For quantification of NeuN+ and TH + cells in IF, stitching images of all brain slide were analyzed in ImageJ software using a ROI in the SNpc of both side to count $NeuN^+$ and TH^+ cells (Cell Counter Plugin).

For 3D-morphometric analysis of microgliosis in the striatum, confocal z-stacks images were acquired using a microscope Leiva DMi8, with a z stack of 1 μm, and an objective HC PL APO 40*X*/1.10 Ester. 3D morphological analysis of microglia was performed with Imaris v10.2.0 (Oxford Instruments) by a blinded investigator. 3D-miocroglial reconstruction was performed using Imaris' Filament Tracer with no loops allowed and soma model. Each image analyzed had between 10 and 12 microglial cells. Largest diameter of starting point was set as 11 μm. Machine learning approach was used for distinguishing between specific and non-specific branching during reconstruction. After reconstruction, each process was verified in a defined cell and manually removed false connections during the machine learning process. Volume thresholding was used to remove small, non-specific projections before completing the reconstruction.

Beam test

We used the beam test previously described.[53] Briefly, the beam apparatus was made of plexiglas and consisted of four continuous horizontal tracts (T1-T4) of 25 cm each (1 m in total). The width of different tracts was 3.5 cm (T1), 3 cm (T2), 2.5 cm (T3), and 2 cm (T4). The beam surface was covered by a metallic grid (1 cm^2). Mice were videotaped while traversing the grid-surface beam from one of the extremes of the beam to the opposite extreme, where the home-cage was located. Two days of training were performed for habituation to the task. The number of errors (# errors) was quantified by watching the videos in slow-motion mode. An error was defined as when a forelimb or hindlimb slipped through the grid and became visible between the grid and the beam surface or on the side of the grid during a forward movement.

Hindlimb clasping reflex

The animals were gently lifted upwards by the midsection of the tail and were observed for ∼5–10 s. Animals were assigned a score of 0, 1, 2, or 3 based on the extent to which the hindlimbs contracted medially, as described before.[29] A score of 0 was assigned to those animals that did not show the hindlimb reflex. A score of 1 was assigned to animals that held one hindlimb inward for the duration of the restraint or if both paws exhibited partial inward grasp. A score of 2 was assigned if both legs were crossed inwards during most of the observation time, but still exhibited some flexibility. A score of 3 was assigned if the animals showed complete paralysis of the hindlimbs that immediately contracted inward and showed no signs of flexibility.

QUANTIFICATION AND STATISTICAL ANALYSIS

The sample size was estimated with the mean and dispersion obtained from preliminary data using the sample size calculator: https://www.stat.ubc.ca/∼rollin/stats/ssize/n2.html. A power of 80% was assumed. All values are expressed as mean ± SEM. Differences between two groups were analyzed using a two-tailed Student's *t* test or, when data were not normally distributed, a non-parametric Mann–Whitney U test. Comparisons among multiple groups were assessed using one- or two-way ANOVA, with time, treatment, or genotype as independent factors. When ANOVA indicated significant differences, pairwise comparisons between means were performed using Dunnett's post hoc test (for comparisons with the control group) or Sidak's post hoc test (for comparisons among all groups). When data were not normally distributed, ANOVA on ranks was applied (Kruskal–Wallis test followed by pairwise comparisons using Dunn's test). The significance of correlations was assessed using Pearson's test when data were normally distributed. A p value ≤ 0.05 was considered statistically significant. Analyses were performed using GraphPad Prism 10 software. p values, n values, and dispersion measurements are indicated in the associated figure legends for each figure.

ADDITIONAL RESOURCES

Supplemental figures and tables are available as supplemental information.

Cell Reports Medicine, Volume *7*

Supplemental information

Preclinical evaluation of nitrated

α-synuclein-specific CAR-Treg therapy

in Parkinson's disease

Valentina Ugalde, Daniela Elgueta, Sandra Espinoza, Ornella Chovar-Vera, Ernesto López, Alejandra Catenaccio, Francisca Álvarez-Astudillo, Dafne Franz, Vanesa Sanchez-Guajardo, María Inés Becker, Diego Figueroa, Zulmary Manjarres, Sofía Hidalgo, Álvaro Lladser, Cheril Tapia-Rojas, Alejandra Loyola, Sergio A. Quezada, Roque Villagra, and Rodrigo Pacheco

Figure S1. Working model hypothesis and aim.

(A–G) Scheme representing the proposed working model of how T cells promote neuroinflammation and neurodegeneration in Parkinson's disease (PD).

(A) Oxidative stress triggers the generation of pathogenic forms of **α**Syn, (B) which aggregate into oligomers and are released into the extracellular space. These **α**Syn oligomers stimulate toll-like receptors on microglia and antigen-presenting cells (APCs), inducing an initial and local inflammatory response.

(C) APCs capture pathogenic forms of **α**Syn, process them, and present their fragments on MHC molecules while migrating to the lymph nodes.

(D) In the lymph nodes, APCs activate $CD8^+$ and $CD4^+$ T cells specific for **α**Syn-derived antigens, which subsequently infiltrate the brain.

(E) $CD4^+$ T cells are re-stimulated by microglia presenting **α**Syn-derived antigens, inducing the production of inflammatory cytokines by T cells, particularly TNF**α** and IFN**γ**. These cytokines promote the production of neurotoxic factors by microglia, thereby favouring neuronal death.

(F) In addition, oxidative stress produced by microglia induces further oxidation and the generation of pathogenic forms of **α**Syn.

(G) Recognition of neurons presenting **α**Syn-derived antigens on MHC class I molecules by $CD8^+$ T cells may induce the release of cytotoxic vesicles, leading to neuronal death.

(H) The aim: we propose to develop regulatory T cells (Treg) expressing chimeric antigen receptors (CAR) specific for the recognition of pathogenic forms of **α**Syn, which, upon antigen recognition, would dampen inflammatory processes and attenuate the progression of neuroinflammation and neurodegeneration.

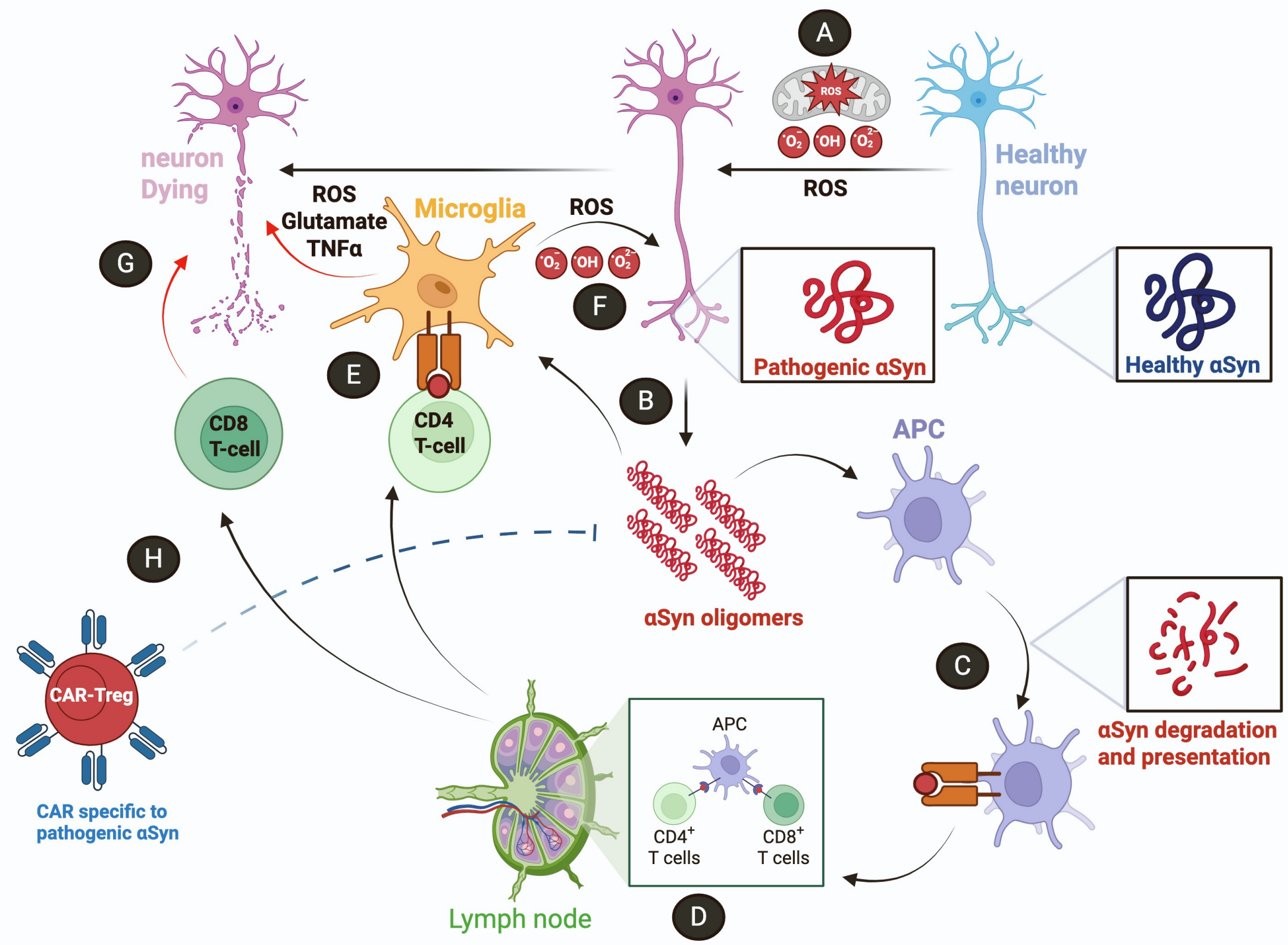

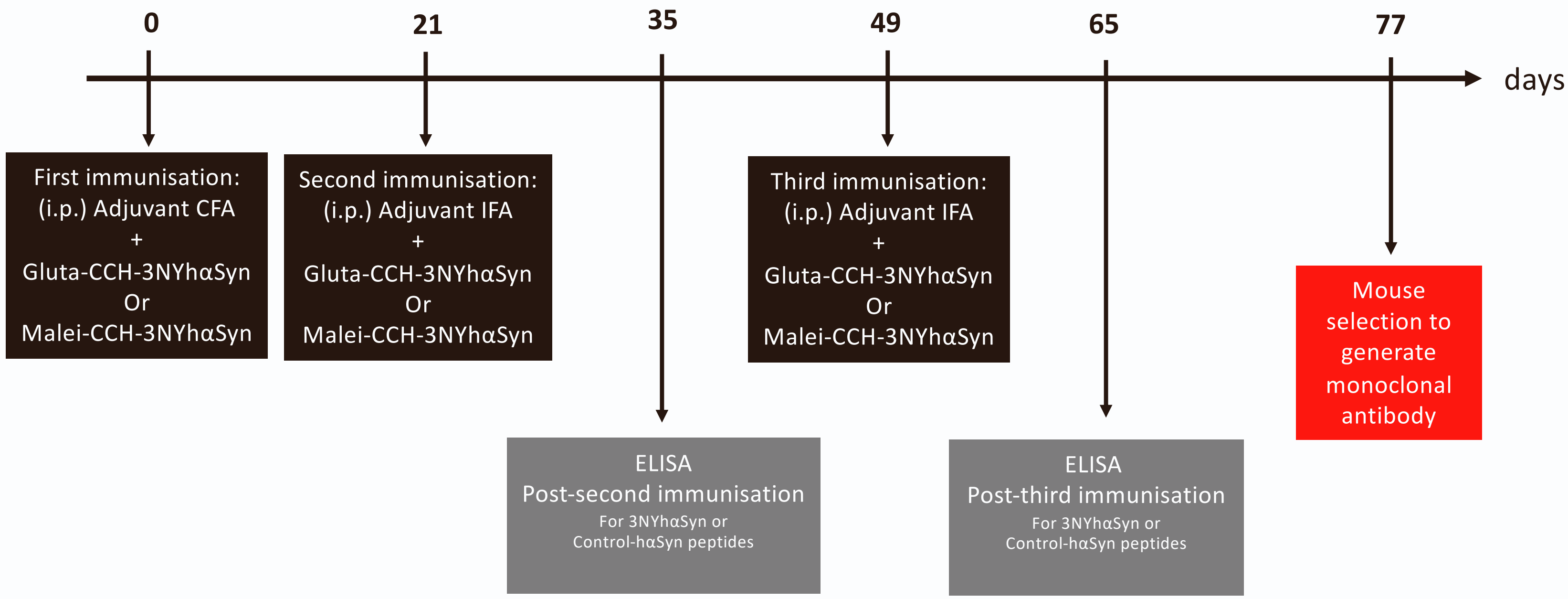

Figure S2. Strategy used for generation of anti-3NYhαSyn mouse polyclonal antibodies. Related to Figure 1 A and B. Scheme representing a timeline of immunisation and analyses to detect anti-3NYhαSyn pAb in serum. The i.p. immunisation with glutaraldehyde CCH-3NYhαSyn (Gluta-CCH-3NYhαSyn; 250 µg of immunogen per mouse), maleimide CCH-3NYhαSyn (250 µg of immunogen per mouse) or PBS was performed using CFA as adjuvant, whereas the second and third immunisation were performed using IFA instead CFA. ELISA analyses were carried out by activating plates with 10 µg/mL of 3NYhαSyn or control-hαSyn peptides after the second and third immunisation. Serial dilutions of serum were assessed starting at 1:10. Immunisations are shown in black boxes, analyses in grey boxes and final outcome in a red box.

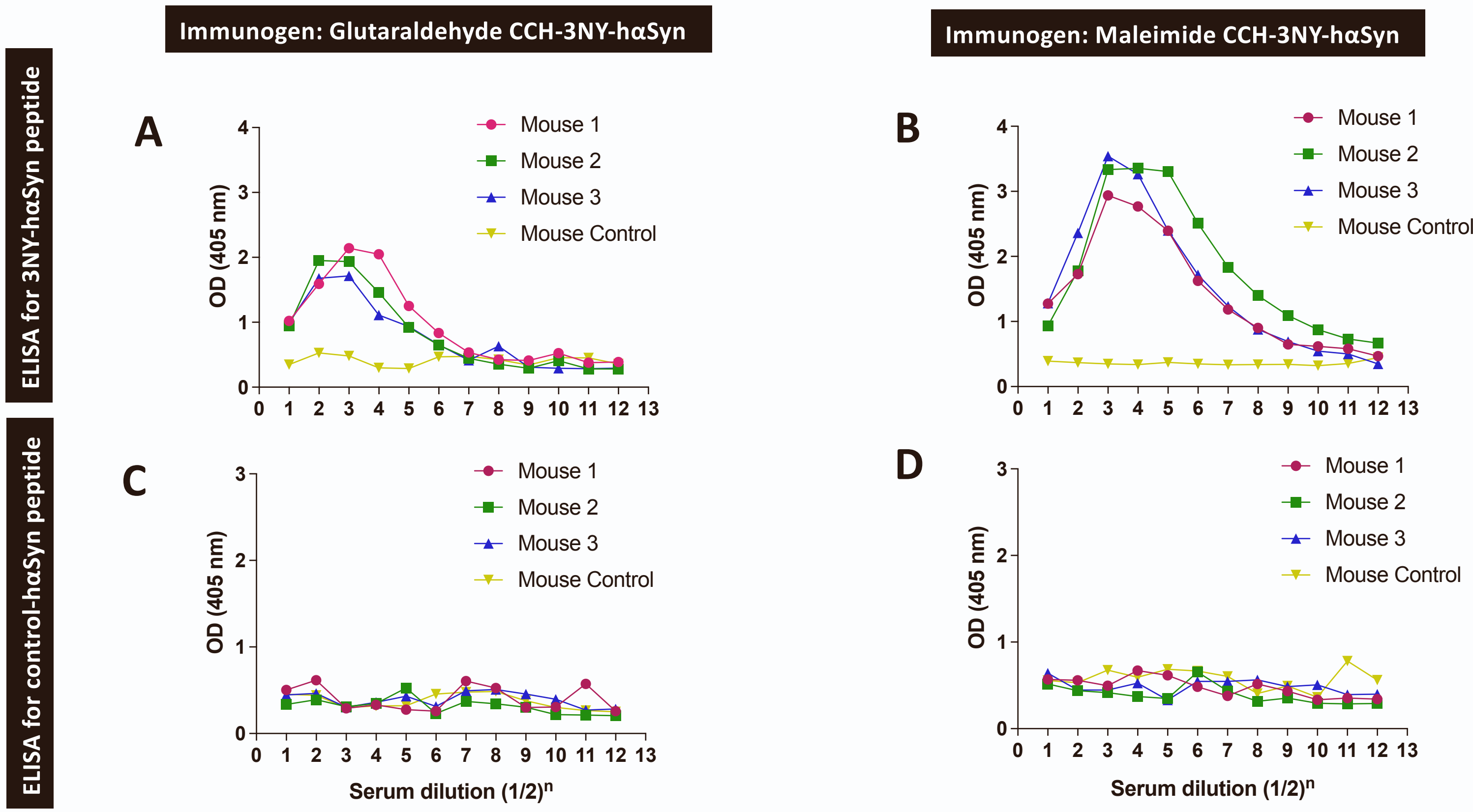

Figure S3. Detection of anti-3NYhαSyn pAb by ELISA to 3NYhαSyn and control-hαSyn peptides after the second immunisation of mice (at day 35). Related to Figure 1 A and B. (**A**) Serum from animals immunised with glutaraldehyde CCH-3NYhαSyn conjugate were assessed for 3NYhαSyn peptide. (**B**) Serum from animals immunised with maleimide CCH-3NYhαSyn conjugate were assessed for 3NYhαSyn peptide. (**C**) Serum from animals immunised with glutaraldehyde CCH-3NYhαSyn conjugate were assessed for control-hαSyn peptide. (**D**) Serum from animals immunised with maleimide CCH-3NYhαSyn conjugate were assessed for control-hαSyn peptide. (A-D) The optic density (O.D.) was detected for serial dilutions of serum obtained from immunised mice (mouse 1-3) or from control mouse (injected with PBS and adjuvant).

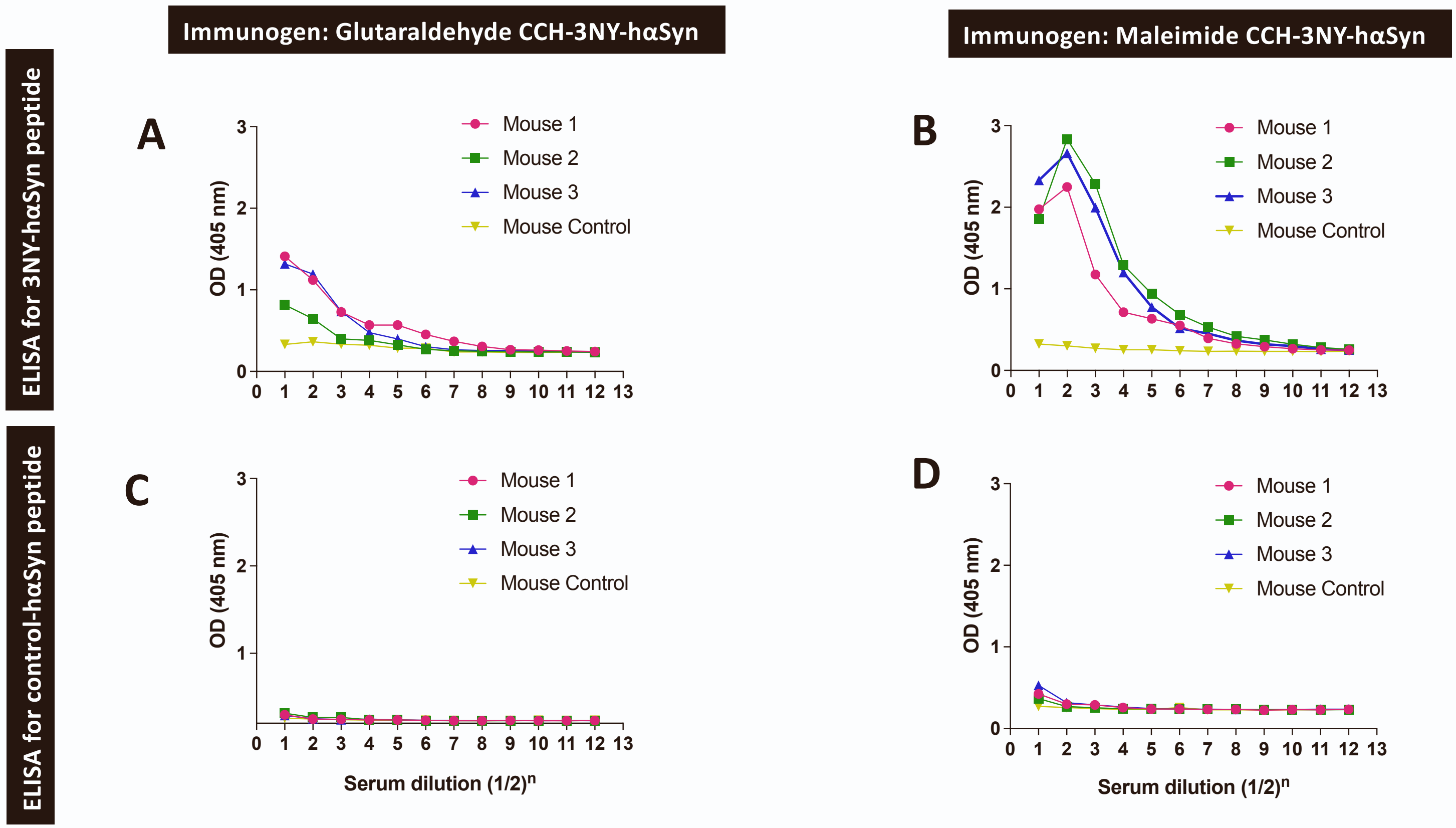

Figure S4. Detection of anti-3NYhαSyn pAb by ELISA for 3NYhαSyn and control-hαSyn peptides after the third immunisation of mice (at day 65). Related to Figure 1 A and B. (**A**) Serum from animals immunised with glutaraldehyde CCH-3NYhαSyn conjugate were assessed for 3NYhαSyn peptide. (**B**) Serum from animals immunised with maleimide CCH-3NYhαSyn conjugate were assessed for 3NYhαSyn peptide. (**C**) Serum from animals immunised with glutaraldehyde CCH-3NYhαSyn conjugate were assessed for control-hαSyn peptide. (**D**) Serum from animals immunised with maleimide CCH-3NYhαSyn conjugate were assessed for control-hαSyn peptide. (A-D) The optic density (O.D.) was detected for serial dilutions of serum obtained from immunised mice (mouse 1-3) or from control mouse (injected with PBS and adjuvant).

A

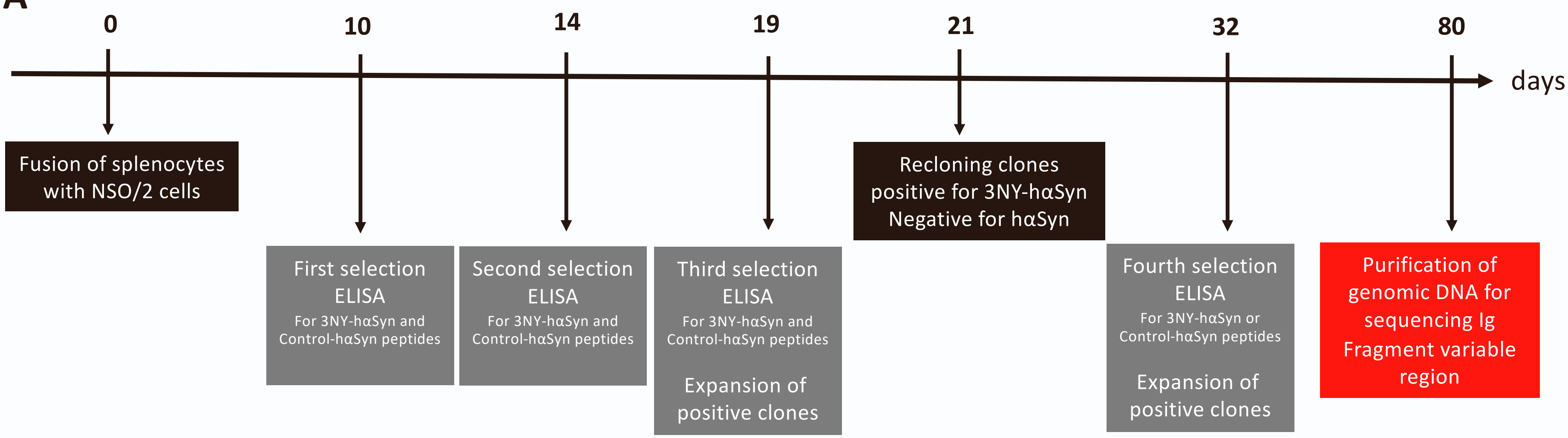

B

Clone	ELISA for 3NY-hαSyn (O.D; mean ± SEM)	ELISA for control-hαSyn (O.D; mean ± SEM)	Heavy chain Isotype	Light chain Isotype
1A 11	2.78 ± 0.57	0.27 ± 0.01	IgG1	Igκ
8E 7	3.34 ± 0.16	0.26 ± 0.01	IgA	Igκ

Figure S5. Generation of anti-3NY-hαSyn mAb. Related to Figure 1 A and B. (**A**) Scheme representing a timeline of generation of hybridomas, expansion and selection of clones by ELISA analyses to detect anti-3NY-hαSyn mAb in hybridomas supernatant. Actions are shown in black boxes, analyses in grey boxes and final outcome in a red box. (**B**) Table showing the two hybriboma clones displaying supernatants with high reactivity to 3NY-hαSyn peptide and negative reactivity to control-hαSyn peptide. Values represent average optic density. The isotype for each clone was determined by the Rapid Antibody Isotyping kit.

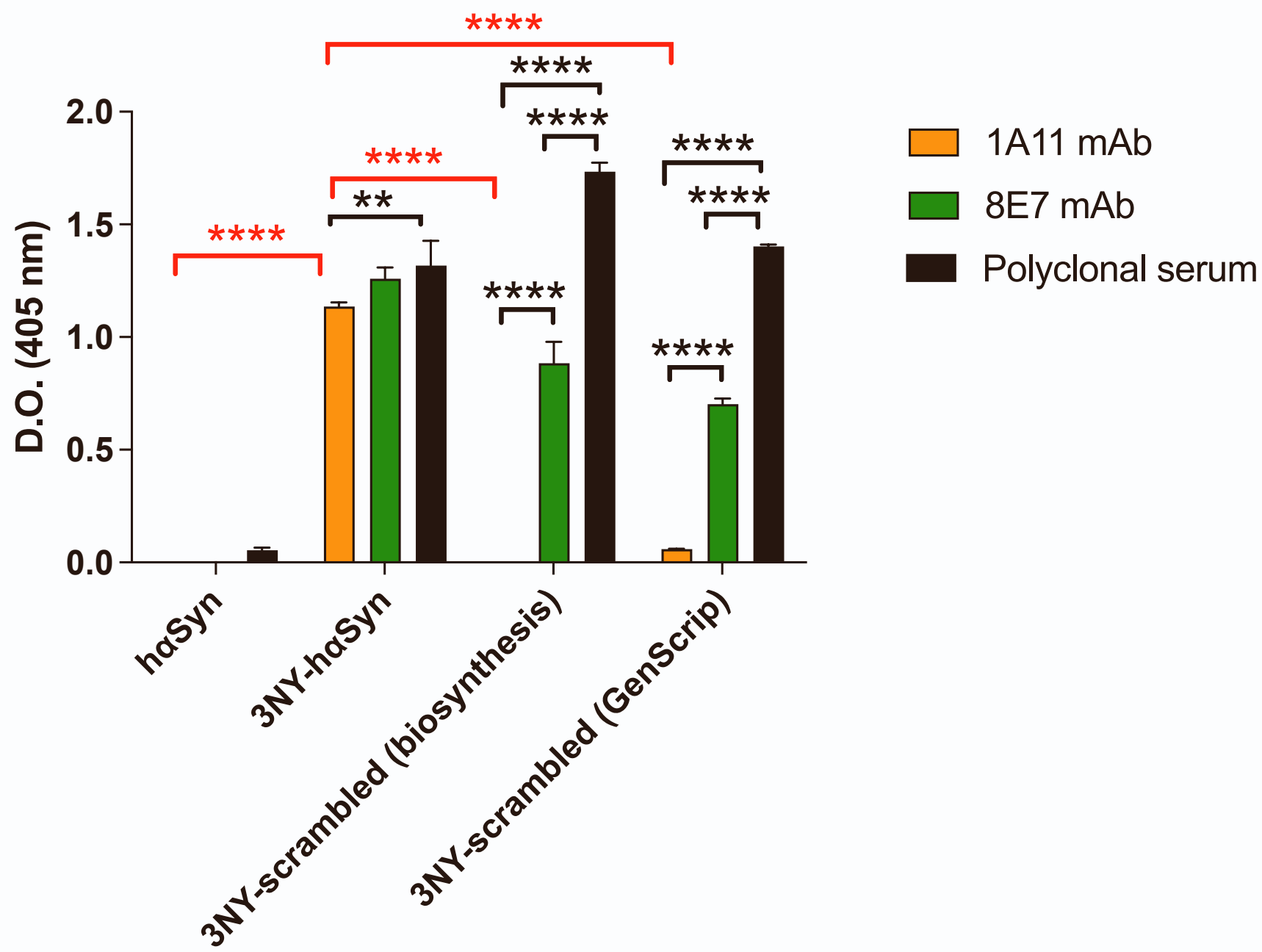

Figure S6. Evaluating the specificity of 1A11 and 8E7 mAbs by ELISA. **Related to Figure 1 A and B.** Serum from animals immunised with maleimide CCH-3NY-hαSyn conjugate (diluted 1:25 in PBS; black bars) or undiluted supernatants from 1A11 (orange bars) or 8E7 (green bars) hybridomas were assessed for reactivity with peptides hαSyn, 3NY-hαSyn or 3NY-scrambled (obtained from biosynthesis or from GenScript). Detection of reactivity was performed by ELISA using a secondary anti-mouse Ig (H+L) antibody conjugated to phosphatase alkaline followed by the addition of p-nitrophenyl phosphate. The optic density was quantified at 405 nm. Values are mean with SEM from triplicates. **, $p<0.01$; ****, $p<0.0001$ by two-way ANOVA followed by Tukey's posthoc test. Differences obtained between different antibodies using the same antigen are indicated in black asterisks/brackets, whilst differences obtained for 1A11 mAb using different antigens are indicated in red asterisks/brackets.

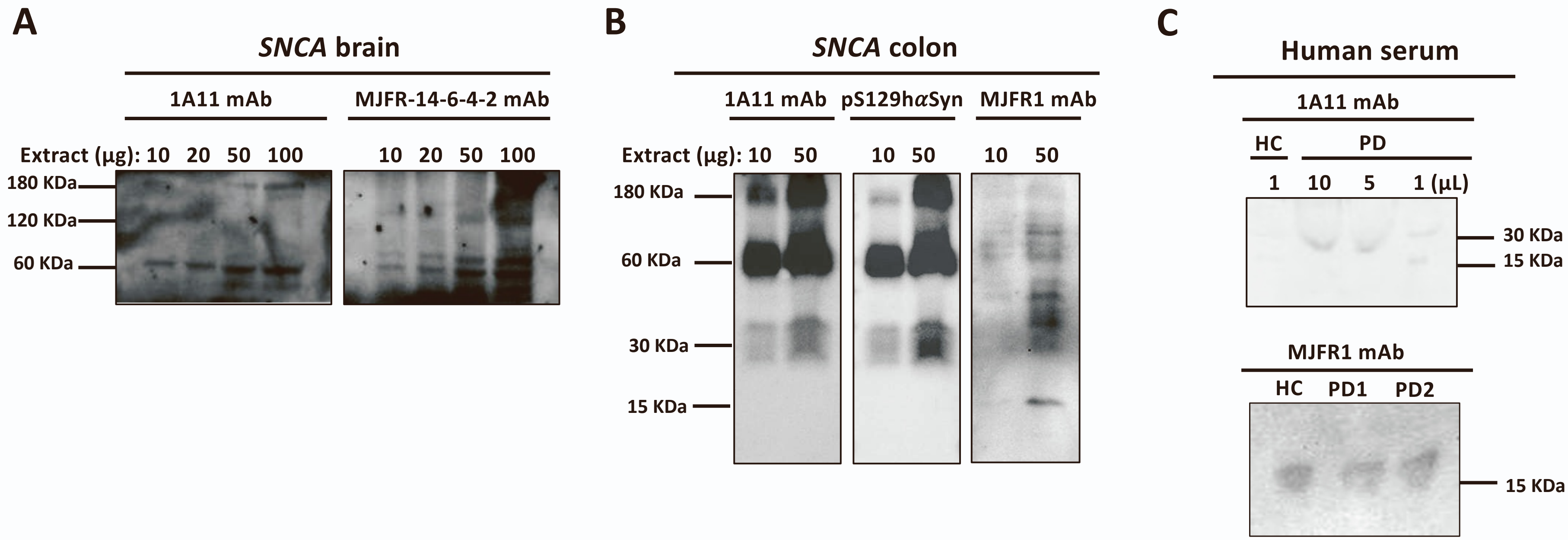

Figure S7. Characterising the 1A11 mAb antigen recognition in biological samples. **Related to Figure 1.** (**A**) Brain lysate obtained from 20-week-old *SNCA* mice was loaded in increasing amounts and separated in SDS-PAGE 10% followed by western blot analysis using the 1A11 mAb or the MJFR-14-6-4-2 mAb (specific for recognition of h**α**Syn aggregates; Abcam, cat# ab209538). (**B**) Colon lysate obtained from 20-week-old SNCA mice was loaded in increasing amounts and separated in SDS-PAGE 12,5% followed by western blot analysis using the 1A11 mAb, pSer129-h**α**Syn (Biolegend) or the MJFR1 mAb (specific for recognition of monomeric **α**Syn; Abcam, cat# ab138501). (**C**) Serum samples obtained from Parkinson's disease patients (PD) or from heathy controls (HC) were loaded and separated in SDS-PAGE 18% followed by western blot analysis using the 1A11 mAb or the MJFR1 mAb. Approximate molecular weights are indicated.

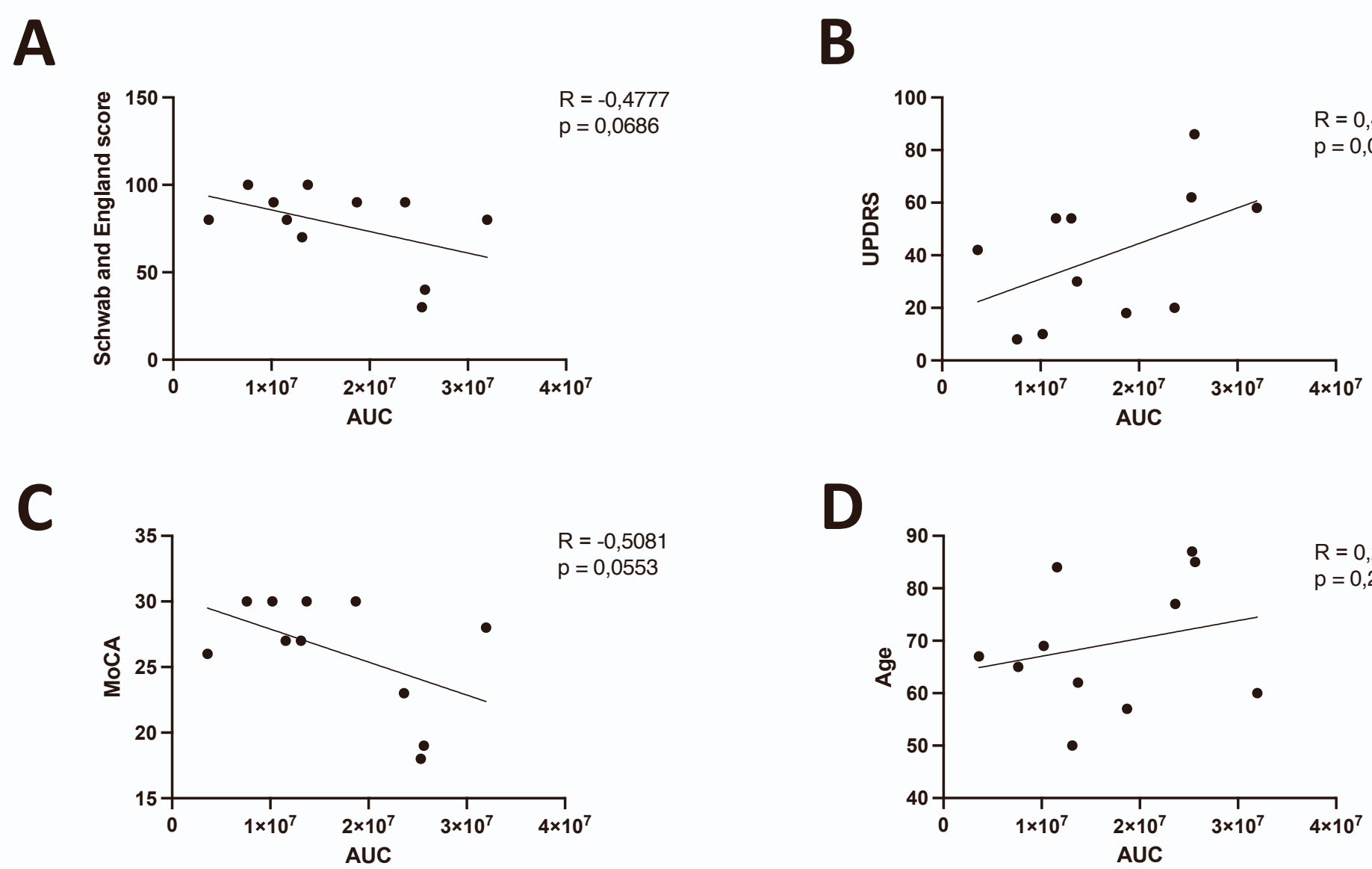

Figure S8. Correlation analysis of the immunoreactivity of the 1A11 mAb with clinical score and age of PD patients. **Related to Figure 1 E.** Dot blot immunoreactivity of 1A11 mAb in serum from PD patients versus clinical score determined by Schwab and England score (**A**), UPDRS (**B**), MoCA test (**C**), and versus the age (**D**). R values are indicated. Each symbol represents data from an individual subject. P values are indicated, as calculated by the Pearson's correlation test.

A

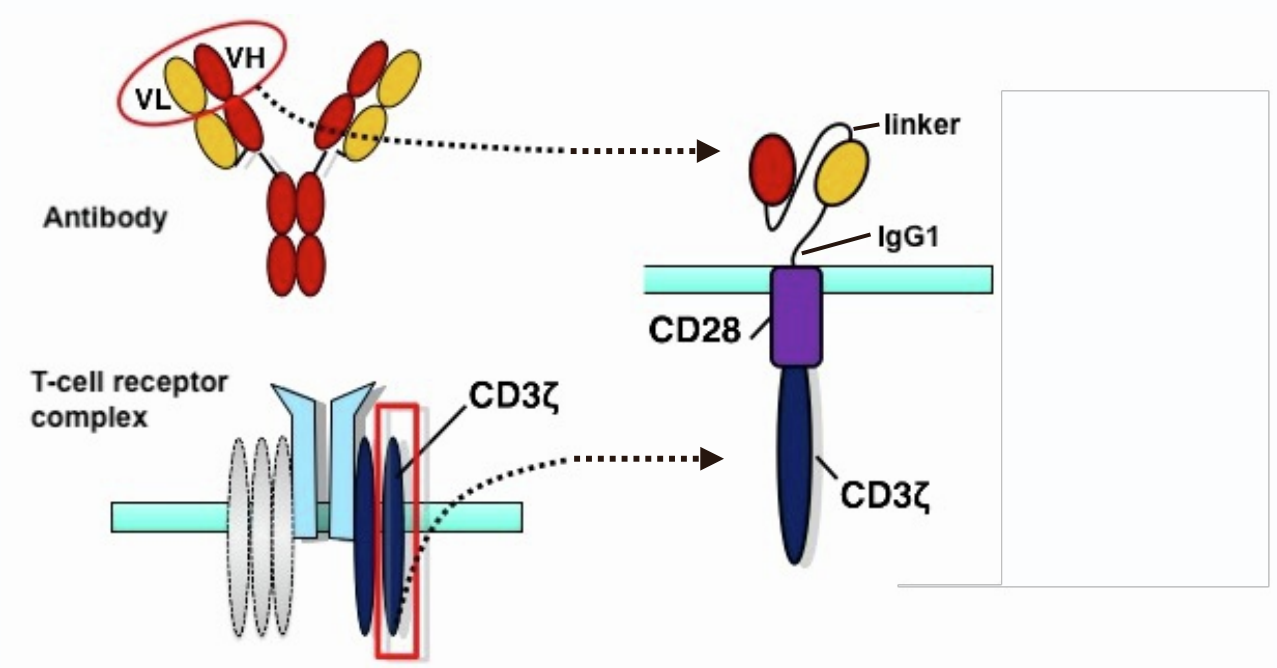

B

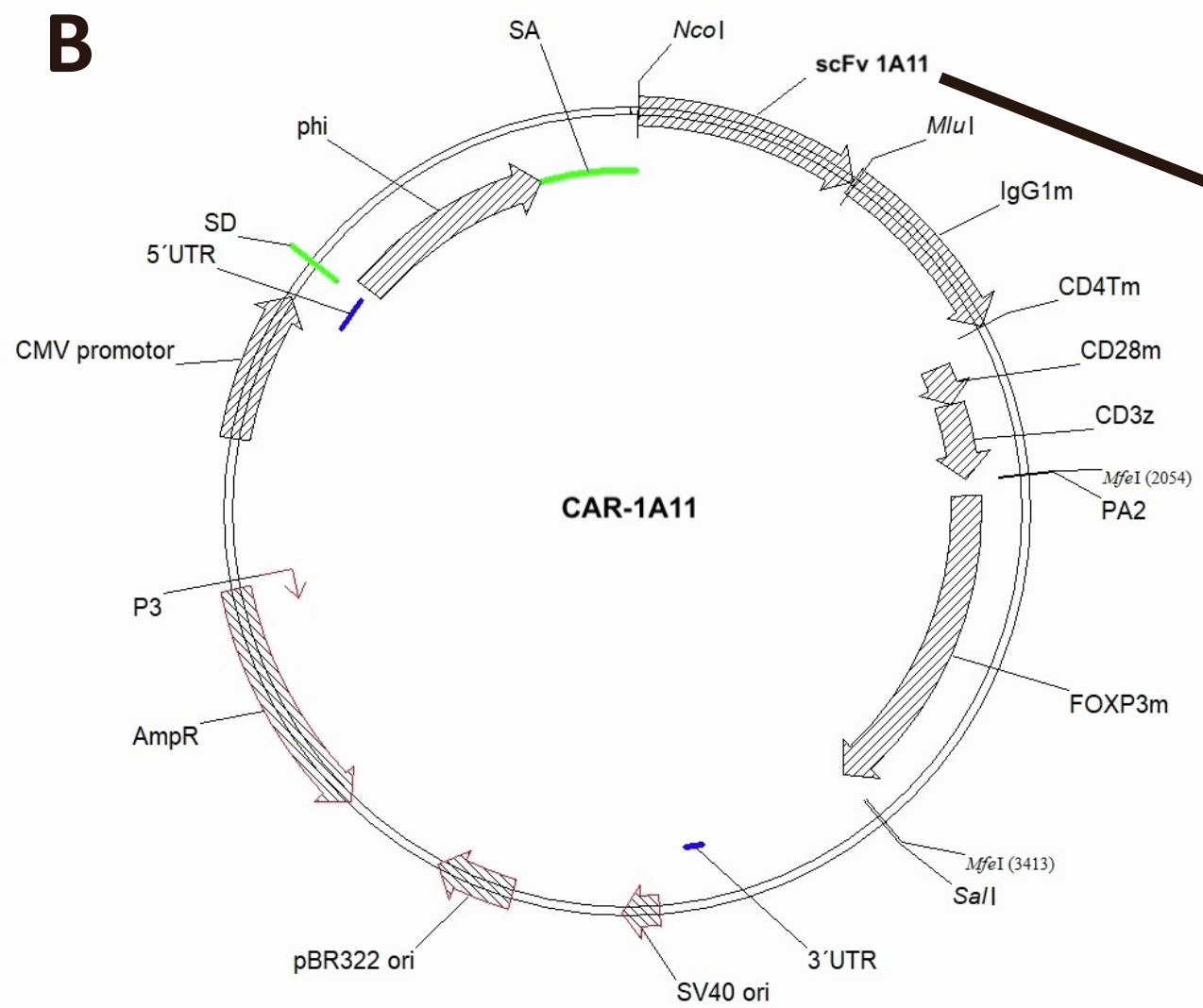

Region	Nucleotide sequence
3'-VL	gatgt…….aacgg
Spacer	ggagg……gatct
VH-5'	gaggt…….cttct

Region	Nucleotide sequence
3'-VH	gaggt…….cttct
Spacer	ggagg……gatct
VL-5'	gatgt…….aacgg

Figure S9. Generation of vectors encoding the CAR1A11. Related to Figure 2. (**A**) Scheme of a Chimeric Antigen Receptor (CAR). (**B**) The left panel shows a map of the vector built to generate regulatory T-cells expressing the CAR1A11. The DNA encoding for a single chain variable fragment (scFv; the antigen-recognising site) of the 1A11 mAb was inserted into a pBullet vector CAR cassette including an extracellular mouse IgG1 hinge domain, CD4 transmembrane domain (CD4Tm) and intracellular CD28 and CD3z moieties responsible for costimulatory and activation signals, respectively. The murine Foxp3 gene was inserted into the construct at the 3' side of the CAR1A11 gene and separated by a 2A peptide. All genes were under the control of a strong promoter (cytomegalovirus; CMV). The right panel shows the abbreviated nucleotide sequences of VH (red) and VL (yellow) from the 1A11 mAb. A spacer sequence required for generating scFv is also indicated (grey). The scFv was arranged in two versions: 3'-VL-spacer-VH-5' and 3'-VH-spacer-VL-5'.

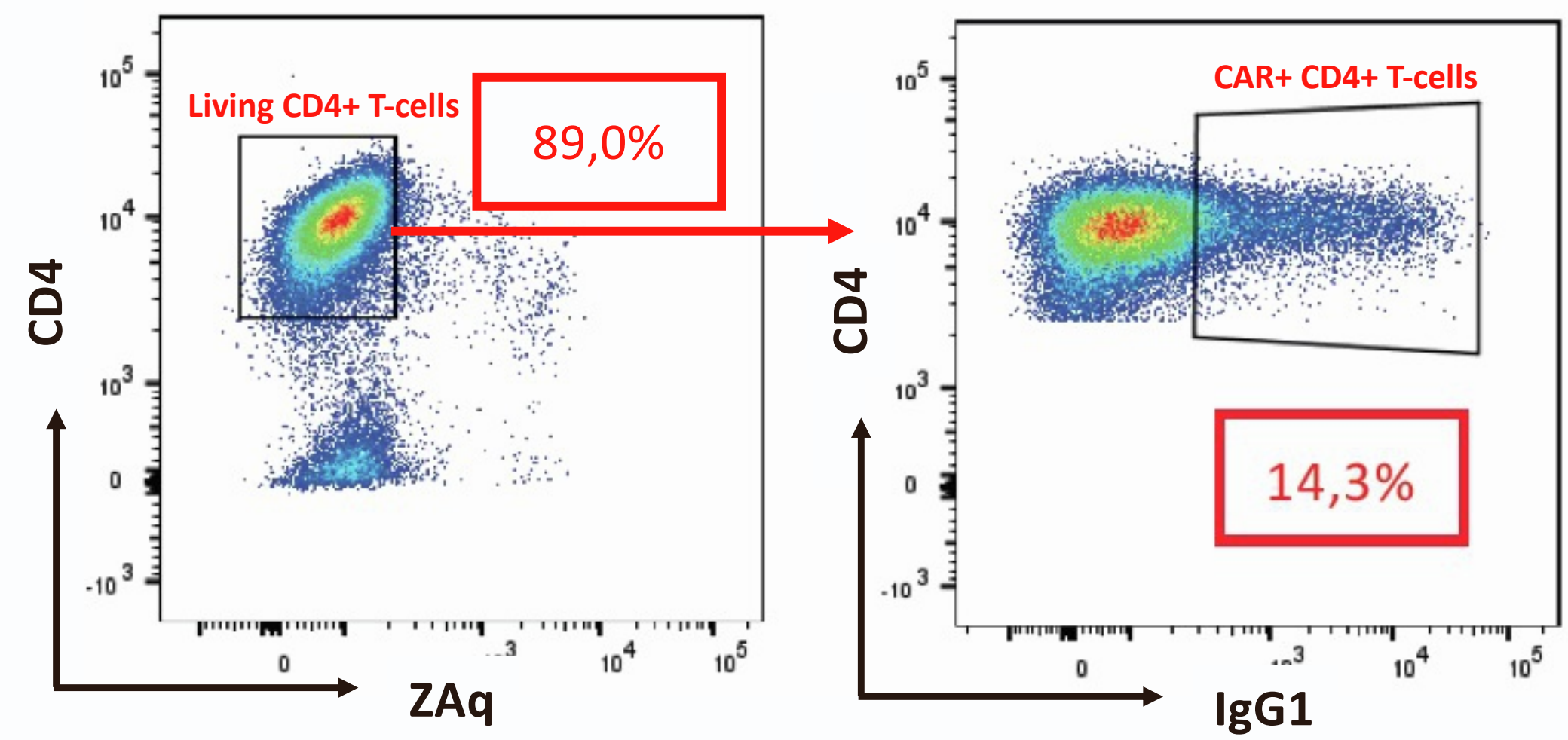

Figure S10. Transduction of CD4⁺ T-cells with viral vectors encoding for CARs. Related to Figure 2. Total $CD3^+$ $CD4^+$ T-cells were isolated from the spleen of WT mice and activated with αCD3/αCD28-coated dynabeads (dynabead-to-cell ratio 1:1) in the presence of IL-2 (1 µg/mL). After 24h, cells were spinoculated over retroviral particles encoding the CAR1A11. The expression of the CAR was evaluated as the extent of surface anti-IgG1 immunoreactivity in living (ZAq^-) $CD4^+$ T-cells. Representative dot-plots are shown. The percentage of cells in the indicated region is shown in red.

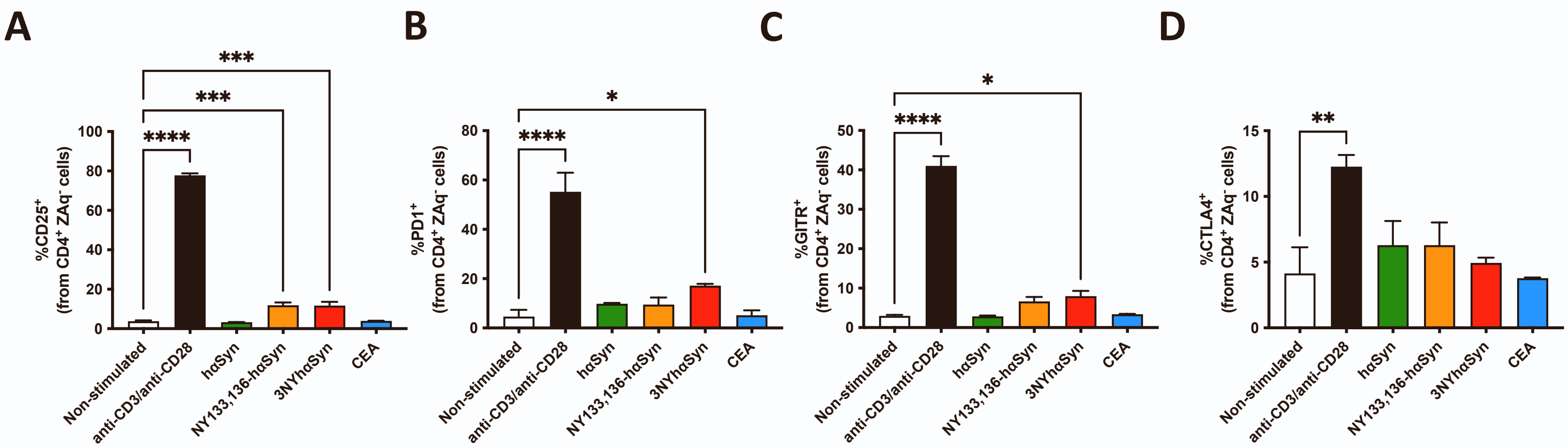

Figure S11. **Characterising CAR-Treg phenotype in response to antigen recognition. Related to Figure 2.** 96-well plates were incubated with hαSyn$_{111-140}$-derived peptides (at 1 µg/mL), including unmodified hαSyn (green), NY133,136-hαSyn (orange), and 3NY-hαSyn (red). As controls, plates were incubated with only vehicle (white), carcinoembrionary antigen (CEA;1 µg/mL, blue), or anti-CD3 (1 µg/mL) plus anti-CD28 (2 µg/mL) (black). Afterwards, CAR-Treg (transduced with the CAR1A11 in the VH-VL conformation; $5x10^4$ cells/well) were incubated with the different antigens for 24 h and the surface expression of CD25 (**A**), PD1 (B), GITR (C) and CTLA4 (D) were determined in live $CD4^+$ T-cells ($CD4^+$ ZAq^- population) by flow cytometry. Data represent the mean ± SEM of triplicates from one out of three experiments with similar results.

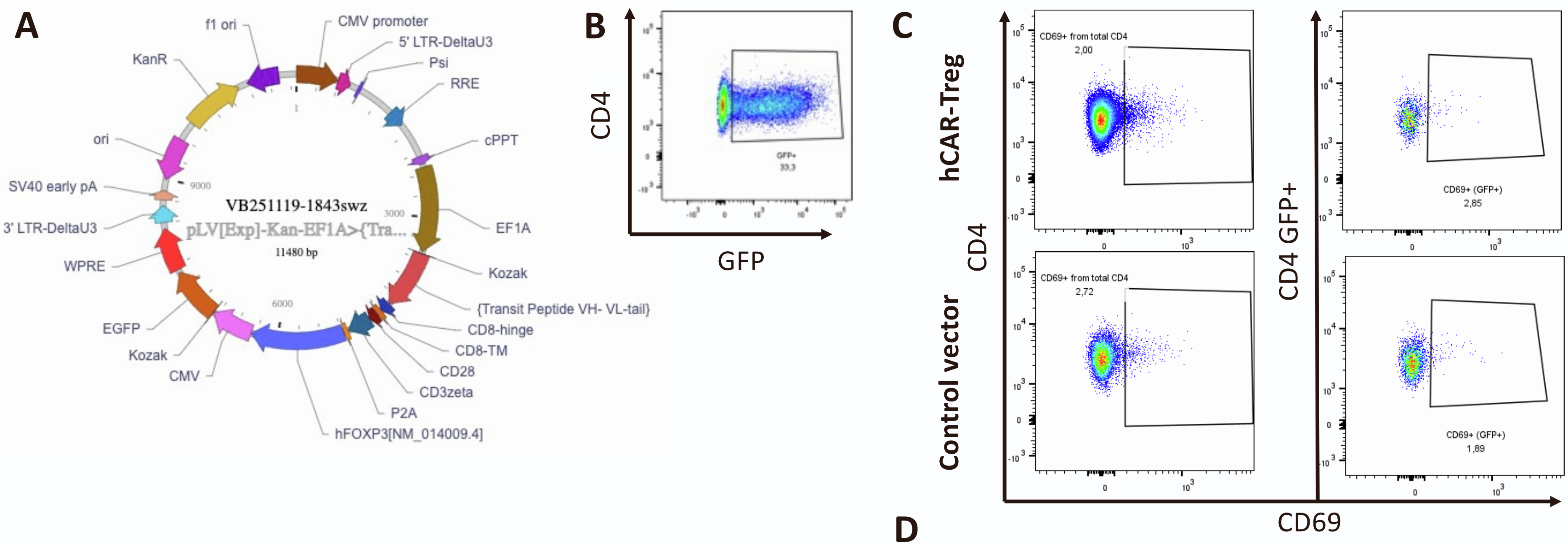

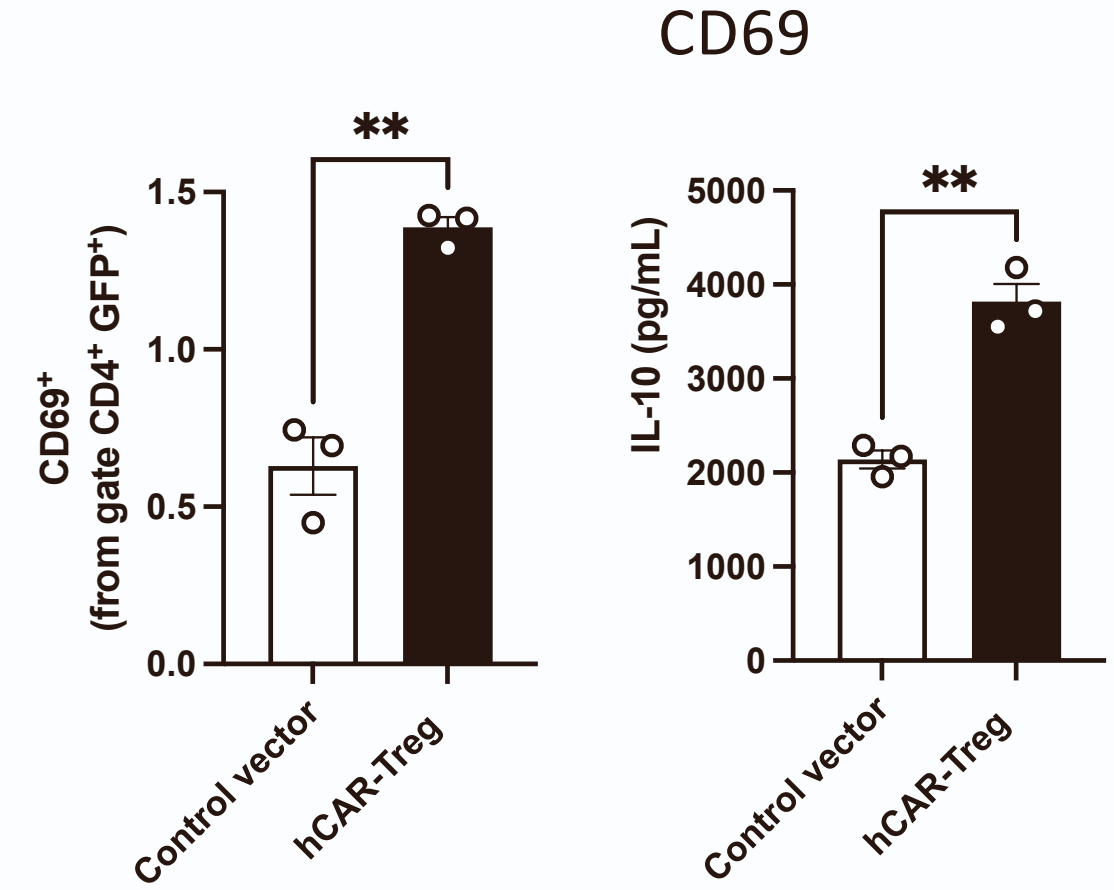

Figure S12. **Human CAR-Treg (hCAR-Treg) becomes activated and release IL-10 in response to antigen recognition. Related to Figure 2.** Human CD4+ T-cells were isolated by cell-sorting from PBMC and then transduced with a lentiviral vector encoding for the human version of the CAR-1A11 (in the VH-VL conformation) bearing the CD3z and CD28 signalling motifs and the human Foxp3 gene. (**A**) Illustration of the lentiviral vector encoding the hCAR, Foxp3 and GFP. (**B**) Representative dot-plot showing the transduction efficiency as determine by the extent of GFP^+ cells in the $CD4^+$ T-cell population by flow cytometry. (C-D) Human CD4+ T-cells transduced with the hCAR-Treg vector or with a control vector (encoding GFP only) were incubated with the 3NY-hαSyn peptide (1 μg/mL) for 24 h. The extent of activation was assessed by the surface CD69 expression and the production of IL-10 was determined in the culture supernatant as a measure of Treg function. (**C**) Representative dot-plots showing the extent of the surface CD69 expression on the CD4+ or the CD4+ GFP+ population. (D) The left panel shows the quantification of the relative CD69 expression in the CD4+ GFP+ population determined by flow cytometry. The right panel shows the quantification of IL-10 in the culture supernatant. Data represent the mean ± SEM of triplicates from one out of three experiments with similar results. **, $p<0.01$ by two-tailed unpaired Student's t-test.

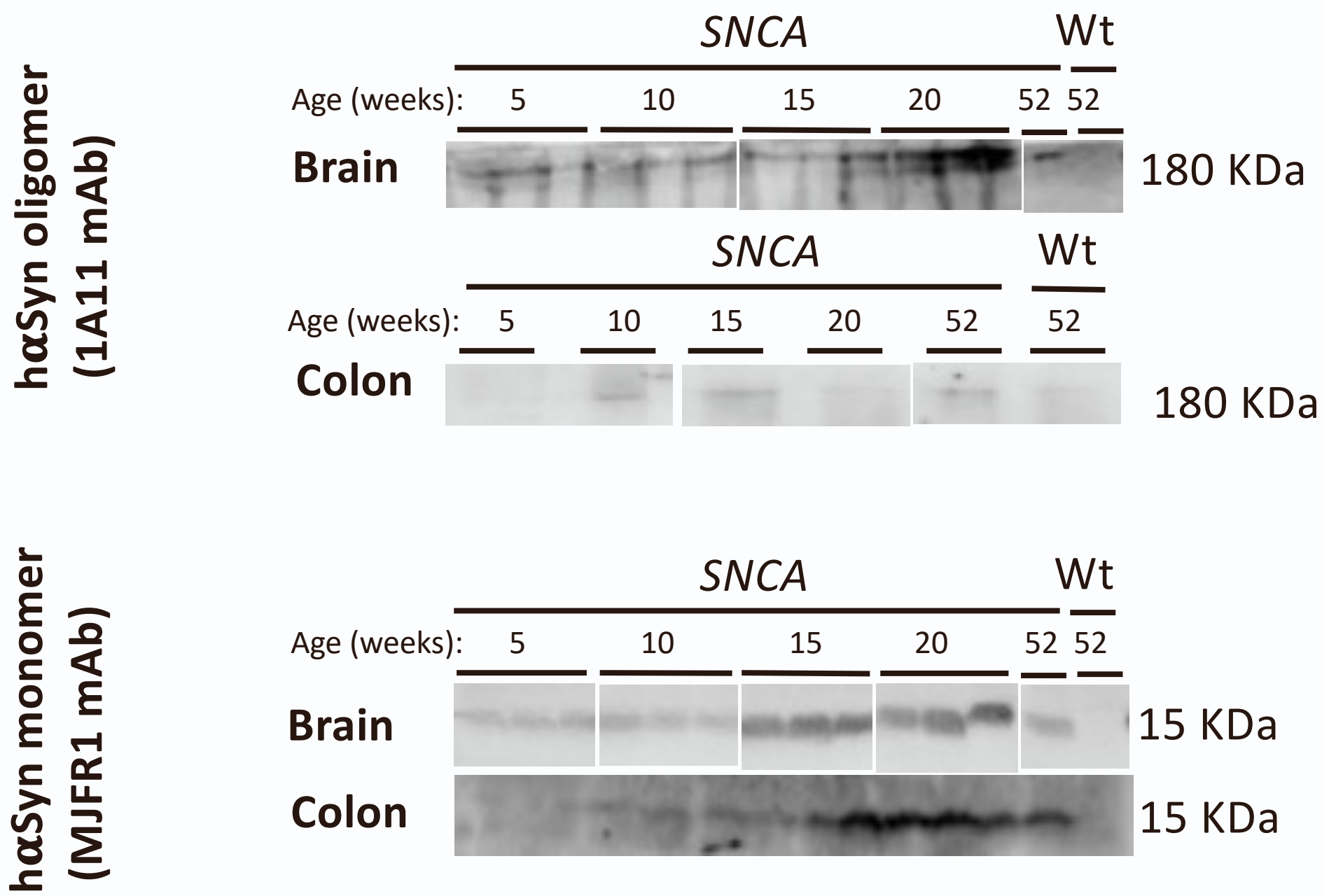

Figure S13. Kinetic analysis of human αSyn expression in brain and colon of *SNCA* mice. Related to Figures 1 and 2. Brain and colon lysates obtained from *SNCA* mice of different ages, or from 1-year old wild-type (Wt) mice were separated in SDS-PAGE 10% followed by western blot analysis using the 1A11 mAb or the MJFR1 mAb (specific for recognition of monomeric αSyn; Abcam, cat# ab138501).

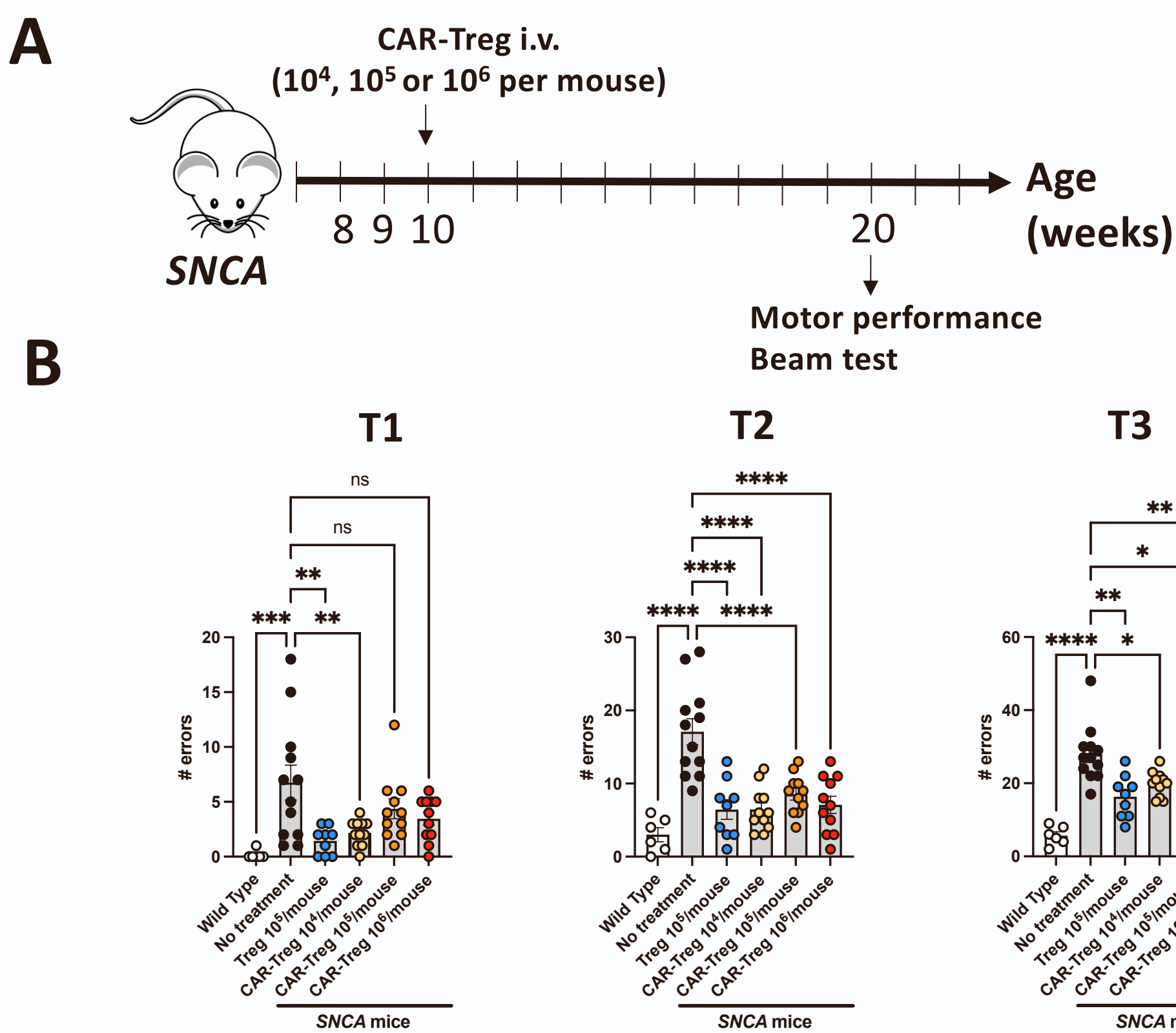

Figure S14. A single injection of the CAR-Treg therapy improves the motor decline in *SNCA* mice. Detailed beam test analysis. Related to Figure 3. Ten weeks old *SNCA* mice were treated with a single i.v. injection of CAR-Treg expressing the CAR1A11 in the VH-VL conformation (10^4 cells/mouse, yellow symbols; 10^5 cells/mouse, orange symbols; 10^6 cells/mouse, red symbols), or polyclonal Treg (10^5 cells/mouse, blue symbols). Non-treated *SNCA* mice (black symbols) were used as controls. Age matched wild-type mice (white symbols) were also evaluated as controls. The motor performance was evaluated at 20 weeks of age by the beam test. (**A**) Illustration of the experimental strategy. (**B**) The number of errors was quantified in each single tract (T1-T4). Each symbol represents an individual mouse (n = 6-12 mice per group). Mean ± SEM are shown. *, p<0.05; **, p<0.01; ***, p<0.001; ****, p<0.0001 compared to the control *SNCA* mice without treatment (black symbols) by one-way ANOVA followed by Dunnett's *post-hoc* test. ns, non-significant.

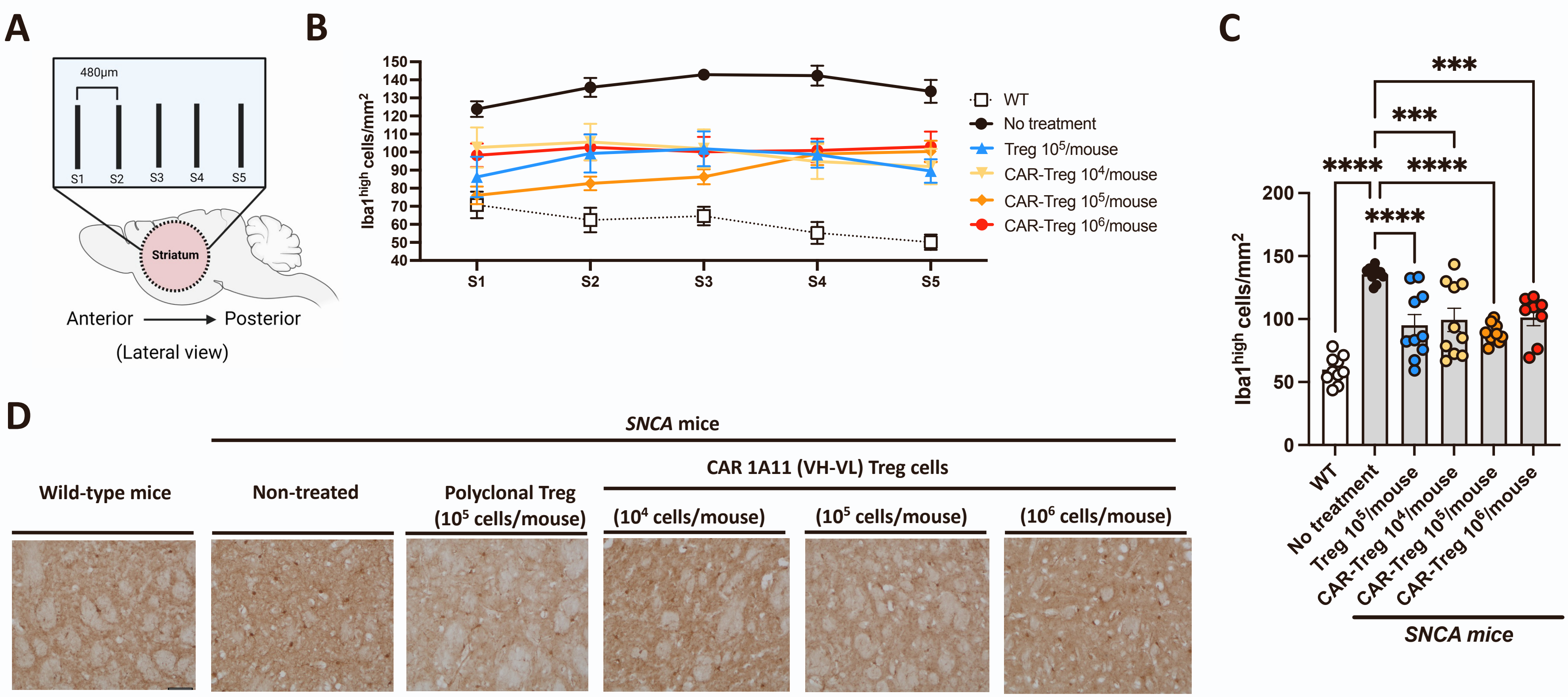

Figure S15. A single injection of CAR-Treg therapy reduces neuroinflammation in *SNCA* mice. Related to Figure 3. Ten weeks old *SNCA* mice were treated with a single i.v. injection of CAR-Treg expressing the CAR 1A11 in the VH-VL conformation (10^4 cells/mouse, beige; 10^5 cells/mouse, orange; 10^6 cells/mouse, red), or polyclonal Treg (10^5 cells/mouse, blue). Non-treated *SNCA* mice (black) or wild-type mice (WT, white) were used as controls. The extent of microgliosis was evaluated at 20 weeks of age by IHC analysis of Iba1 along the striatal antero-posterior axis. (**A**) Scheme illustrating the analysis of five different sections (S1-S5), separated by 480 µm, along the striatum. (B, C) Quantifying of the number of cells displaying high immunoreactivity for Iba1 per area in every single section (**B**) or the average from all the sections together (**C**). Each symbol represents an individual mouse (n = 8-10 mice per group). Mean ± SEM are shown. ***, p<0.001; ****, p<0.0001 by one-way ANOVA followed by Tukey's *post-hoc* test. (**D**) Representative images from section 3 are shown for each experimental group. Bar scale, 100 µm.

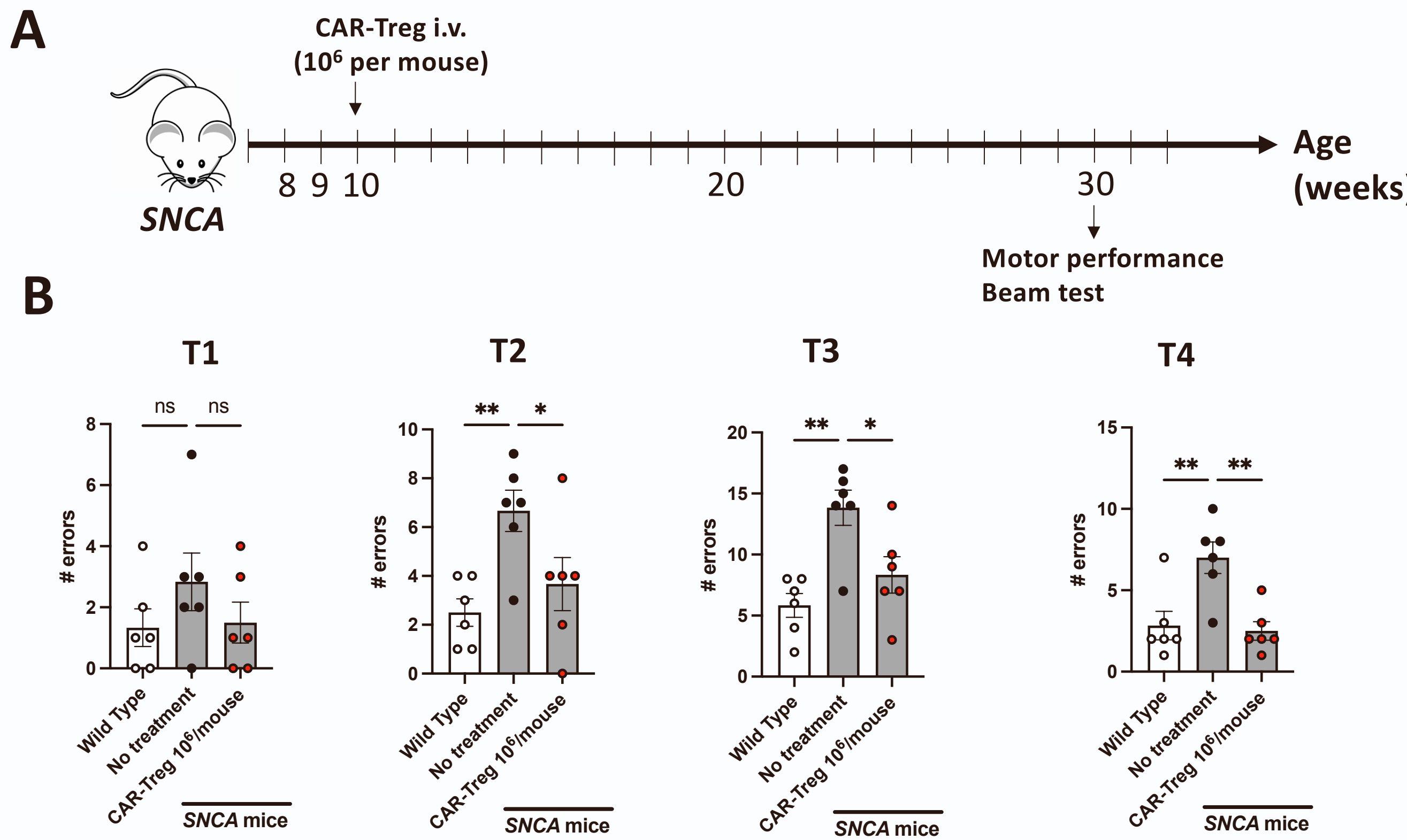

Figure S16. A single injection of the CAR-Treg therapy improves the motor decline in *SNCA* mice even 20 weeks after the treatment: Detailed beam test analysis. Related to Figure 3. Ten weeks old *SNCA* mice were treated with a single i.v. injection of CAR-Treg expressing the CAR1A11 in the VH-VL conformation (10^6 cells/mouse, red symbols). Non-treated *SNCA* mice (black symbols) were used as controls. Age matched wild-type mice (white symbols) were also evaluated as controls. The motor performance was evaluated at 30 weeks of age by the beam test. (**A**) Illustration of the experimental strategy. (**B**) The number of errors was quantified in each single tract (T1-T4). Each symbol represents an individual mouse (n = 6 mice per group). Mean ± SEM are shown. *, p<0.05; **, p<0.01 compared to the control *SNCA* mice without treatment (black symbols) by one-way ANOVA followed by Dunnett's *post-hoc* test. ns, non-significant.

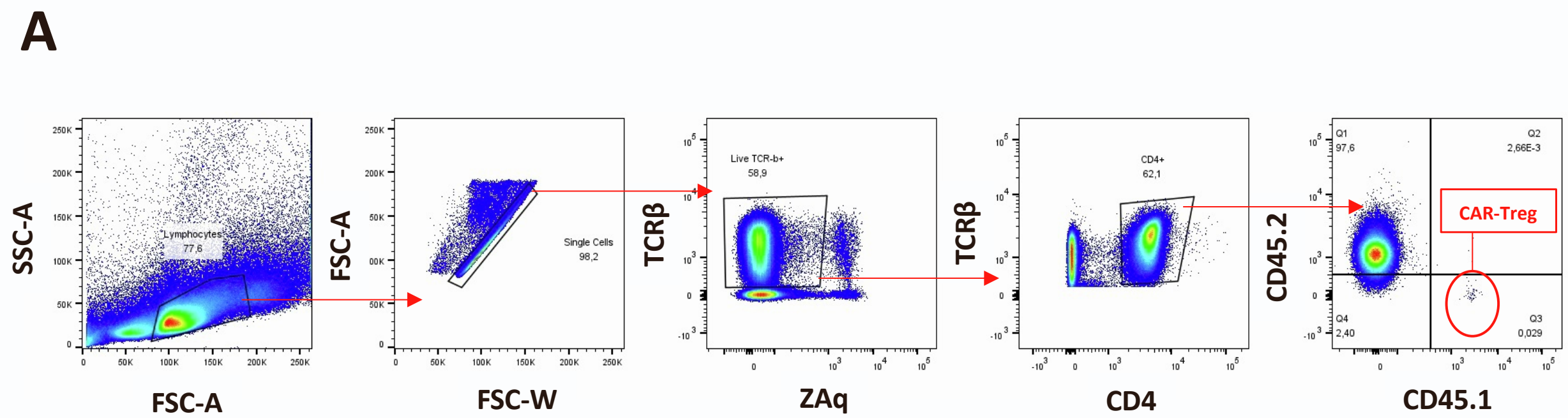

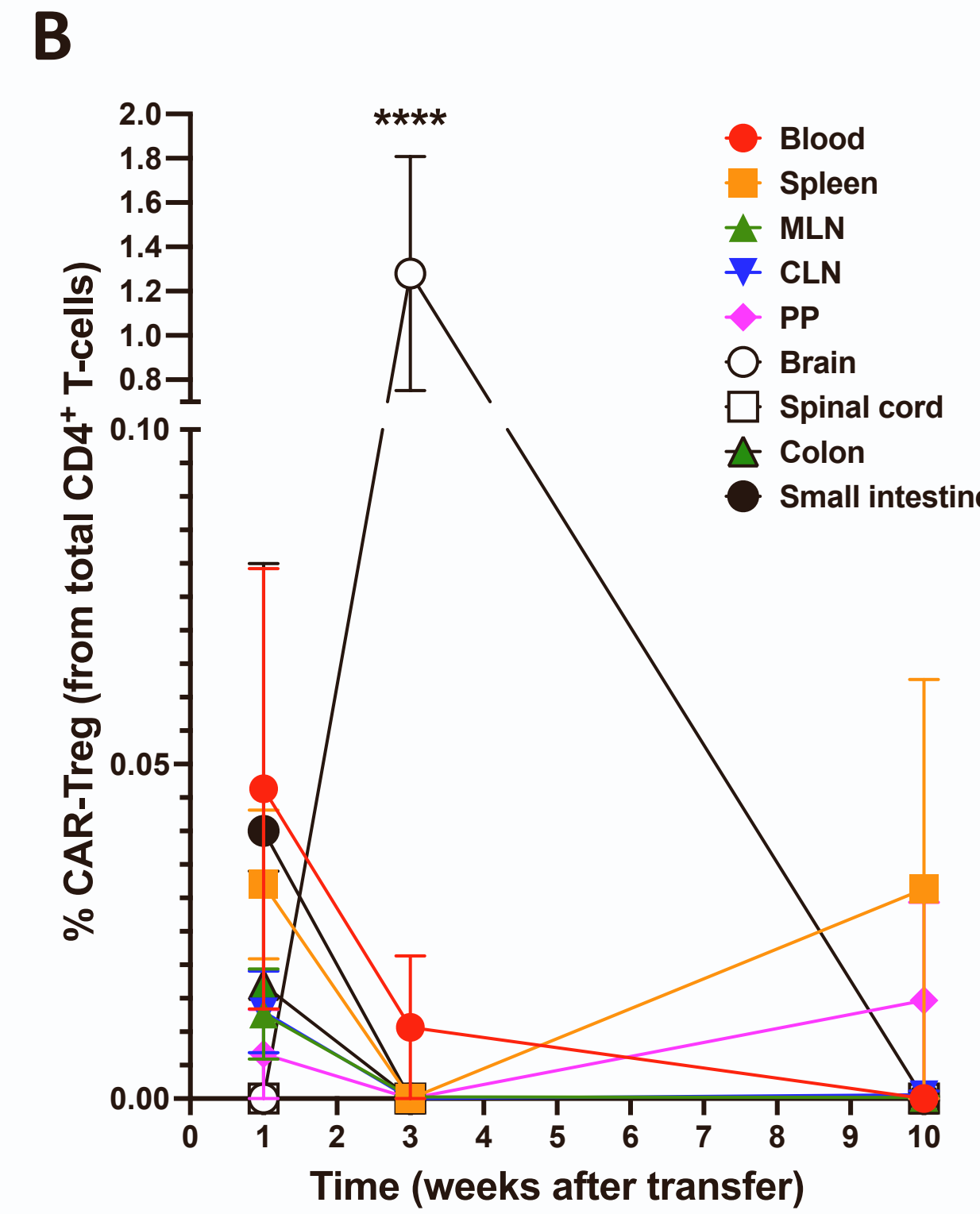

Figure S17. Spatiotemporal analysis of the distribution of CAR-Treg cells in *SNCA* mice. Related to Figure 3. Ten-week-old *SNCA* mice (CD45.2$^{+/+}$) received a single injection of congenic CAR-Treg cells (10^6 cells/mouse; CD45.1$^{+/+}$). One, three, or ten weeks later, mice were sacrificed and the presence of CAR-Treg cells was tracked by flow cytometry in different tissues, including blood, spleen, mesenteric lymph nodes (MLN), cervical lymph nodes (CLN), Peyer's patches (PP), brain, spinal cord, colonic mucosa, and small-intestinal mucosa. (**A**) Gating strategy used to analyse transferred cells (CD45.1$^+$) within the live (ZAq$^-$) CD4$^+$ T-cell population of each tissue. (**B**) Quantification of the relative percentage of donor cells (CD45.1$^+$) within the total CD4$^+$ T-cell population analysed in each tissue. Data represent mean ± SEM of three mice per group. ****, $p<0.0001$ compared to the percentage of CAR-Treg in the blood as determined by two-way ANOVA followed by Dunnett's post-hoc test.

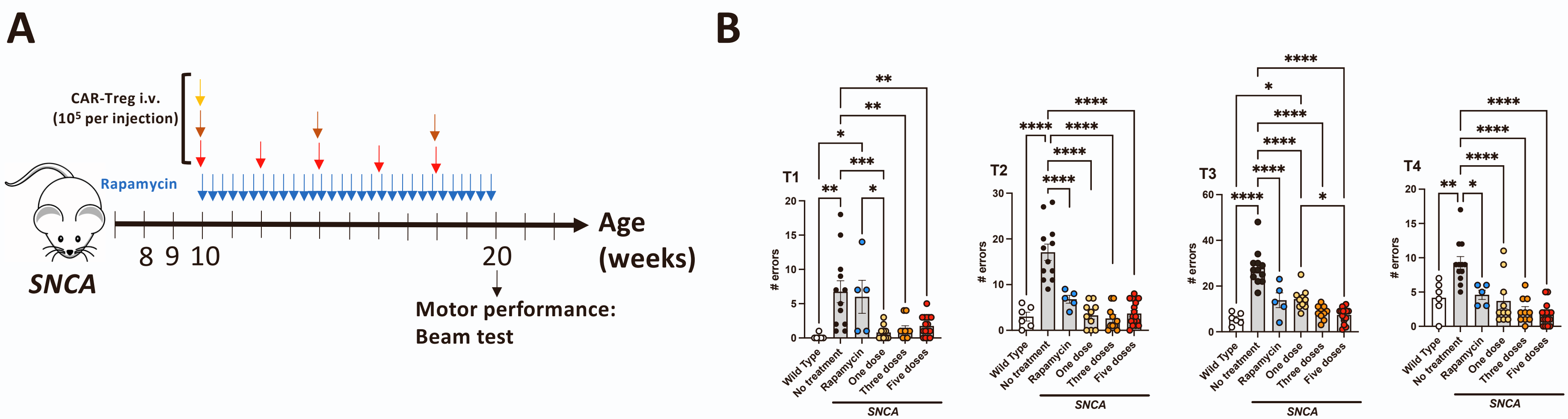

Figure S18. Evaluation of the efficacy of CAR-Treg therapy administered at different number of injections in *SNCA* mice at the level of motor performance. **Detailed beam test analysis. Related to Figure 3.** Ten weeks old *SNCA* mice (grey bars) were treated with a single injection (at week 10; beige symbols), three injections (at weeks 10, 14 and 18; orange symbols), or five injections (at weeks 10, 12, 14, 16, 18; red symbols) of i.v. CAR-Treg expressing the CAR1A11 in the VH-VL conformation (10^5 cells/mouse). *SNCA* mice treated with rapamycin (7,5 mg/Kg i.p. three times a week for 10 weeks, blue symbols) or non-treated (black symbols) were used as controls. Age matched wild-type mice (white bars, white symbols) were also evaluated as controls. At the week 20 of age, the motor performance was quantified by the beam test. (**A**) Illustration of the experimental strategy. (**B**) The number of errors in each single tract (T1-T4) quantified in the beam test. Each symbol represents an individual mouse (n = 5-16 mice per group). Mean ± SEM are shown. *, p<0.05; **, p<0.01; ***, p<0.001; ****, p<0.0001 by one-way ANOVA followed by Tukey's *post-hoc* test.

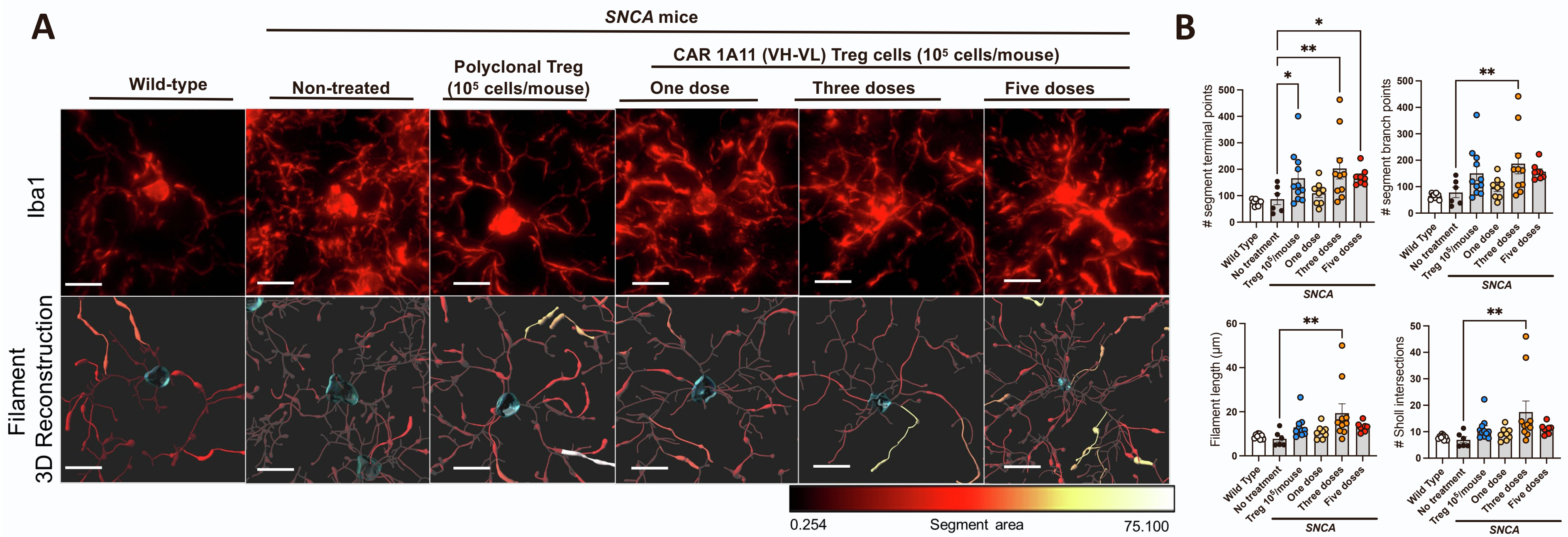

Figure S19. Morphometric analysis of microglia in *SNCA* mice treated with CAR-Treg therapy. Related to Figure 4. Ten weeks old *SNCA* mice (grey bars) were treated with a single injection (at week 10; yellow symbols), three injections (at weeks 10, 14 and 18; orange symbols), or five injections (at weeks 10, 12, 14, 16, 18; red symbols) of i.v. CAR-Treg expressing the CAR1A11 in the VH-VL conformation (10^5 cells/mouse). *SNCA* mice treated with a single injection of polyclonal Treg (10^5 cells/mouse, blue symbols) or non-treated (black symbols) were used as controls. Age matched wild-type mice (white bars, white symbols) were also evaluated as controls. At the week 20 of age, striatal microglia was identified by Iba1 fluorescent immunostaining and morphometric analysis was conducted. (**A**) Representative maximum intensity projections from confocal images showcasing microglial morphology are shown in top panels. Representative three-dimensional reconstructions (filament tool) of microglia from the different groups using Imaris are shown in bottom panels using color codes based on statistics of the filament area are shown in bottom panels. Scale bar: 10 µm. (**B**) Several morphometric quantifications are shown, including number of segment terminal points, number of segment branch points, filament length and number of sholl intersections. Each symbol represents an individual slice (four slices per mouse; n = 3-5 mice per group). Mean ± SEM are shown. *, $p<0.05$; **, $p<0.01$ compared to the control (non-treated *SNCA* mice; black symbols) by one-way ANOVA.

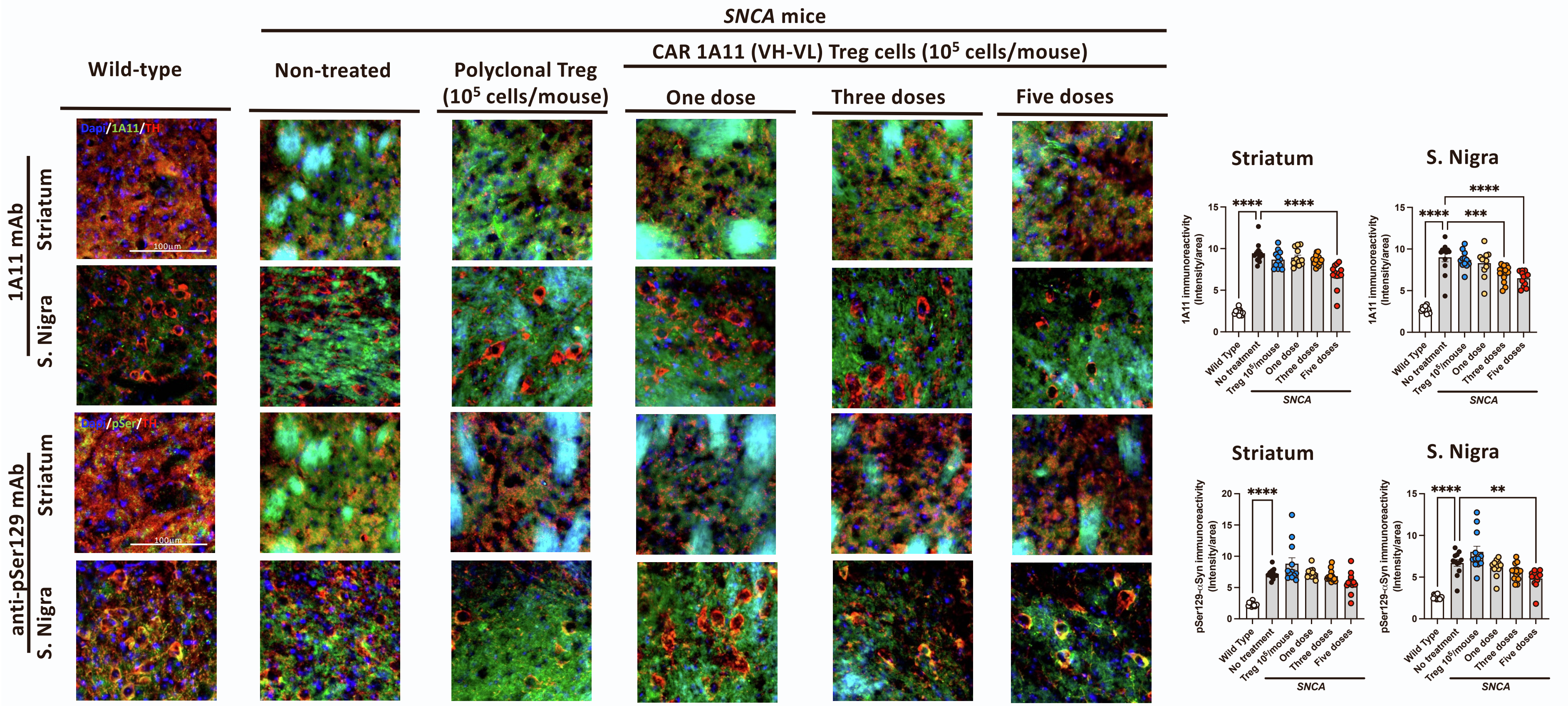

Figure S20. Synuclein pathology is reduced in the nigrostriatal pathway of *SNCA* mice treated with multiple injections of CAR-Treg therapy. Related to Figures 3 and 4. Ten weeks old *SNCA* mice (grey bars) were treated with a single injection (at week 10; yellow symbols), three injections (at weeks 10, 14 and 18; orange symbols), or five injections (at weeks 10, 12, 14, 16, 18; red symbols) of i.v. CAR-Treg expressing the CAR1A11 in the VH-VL conformation (10^5 cells/mouse). *SNCA* mice treated with a single injection of polyclonal Treg (10^5 cells/mouse, blue symbols) or non-treated (black symbols) were used as controls. Age matched wild-type mice (white bars, white symbols) were also evaluated as controls. At the week 20 of age, the immunoreactivity of the mAb 1A11 and pSer129-**α**Syn were analysed in the SN and striatum by immunofluorescence. TH and nuclei were also immunostained. Representative images are shown in left panels. Bar scale, 100 µm. The fluorescence intensity associated to mAb 1A11 and pSer129-**α**Syn normalised per area was quantified in right panels. Each symbol represents an individual slice (three slices per mouse; n = 5-6 mice per group). Mean ± SEM are shown. **, p<0.01; ***, p<0.001; ****, p<0.0001 compared to the control (non-treated *SNCA* mice; black symbols) by one-way ANOVA followed by Dunnett's *post-hoc* test.

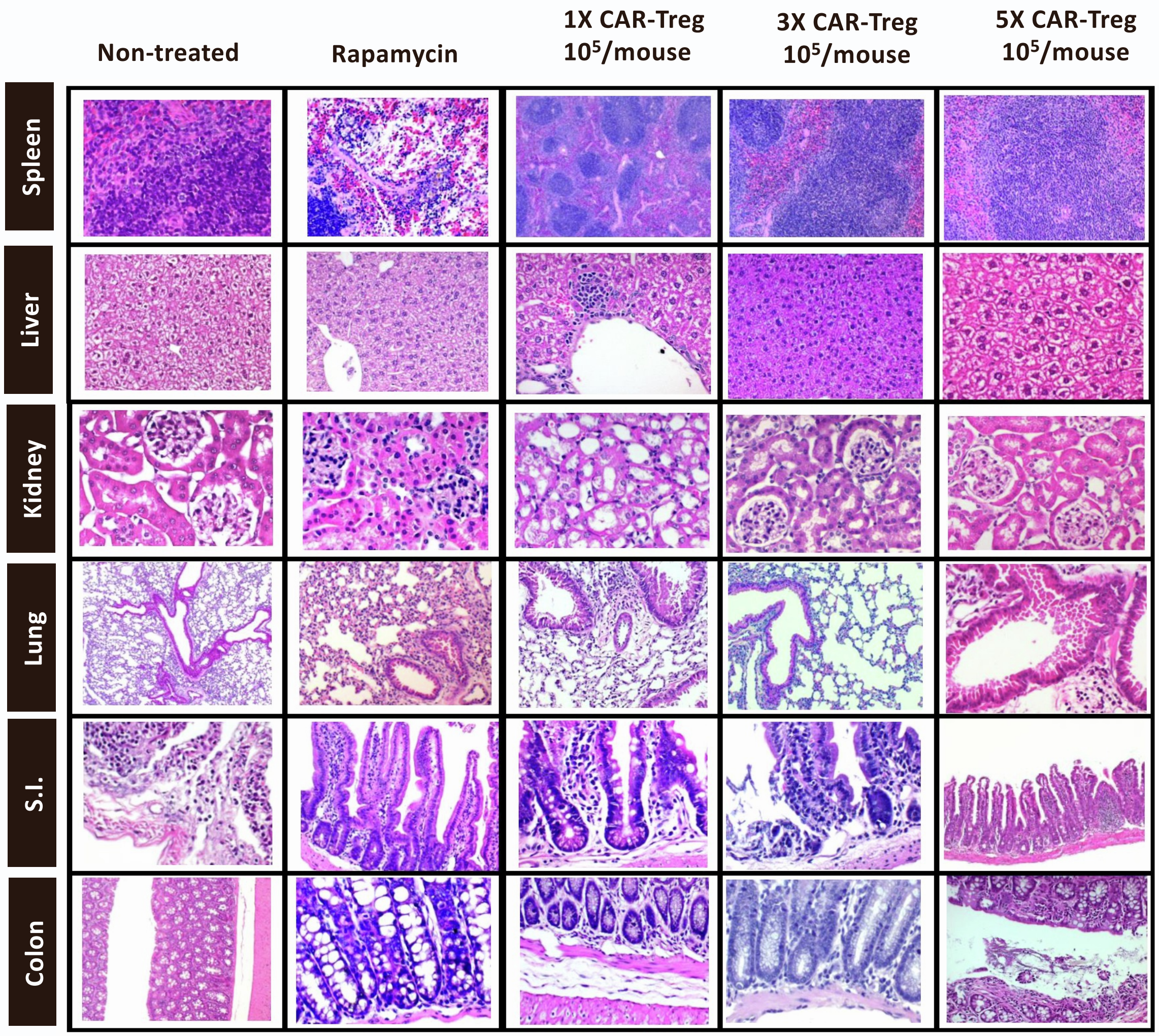

Figure S21. Histopathological analysis of different organs from *SNCA* mice treated with CAR-Treg therapy. **Related to Figures 3 and 4.** *SNCA* mice were treated as shown in figure 3A. At 20 weeks of age, mice were sacrificed and histological analysis was performed on the liver, spleen, kidney, lung, small intestine (S.I.), and colon. Representative images are shown. The group of mice treated with rapamycin was the only one displaying histological anomalies in most organs analysed.

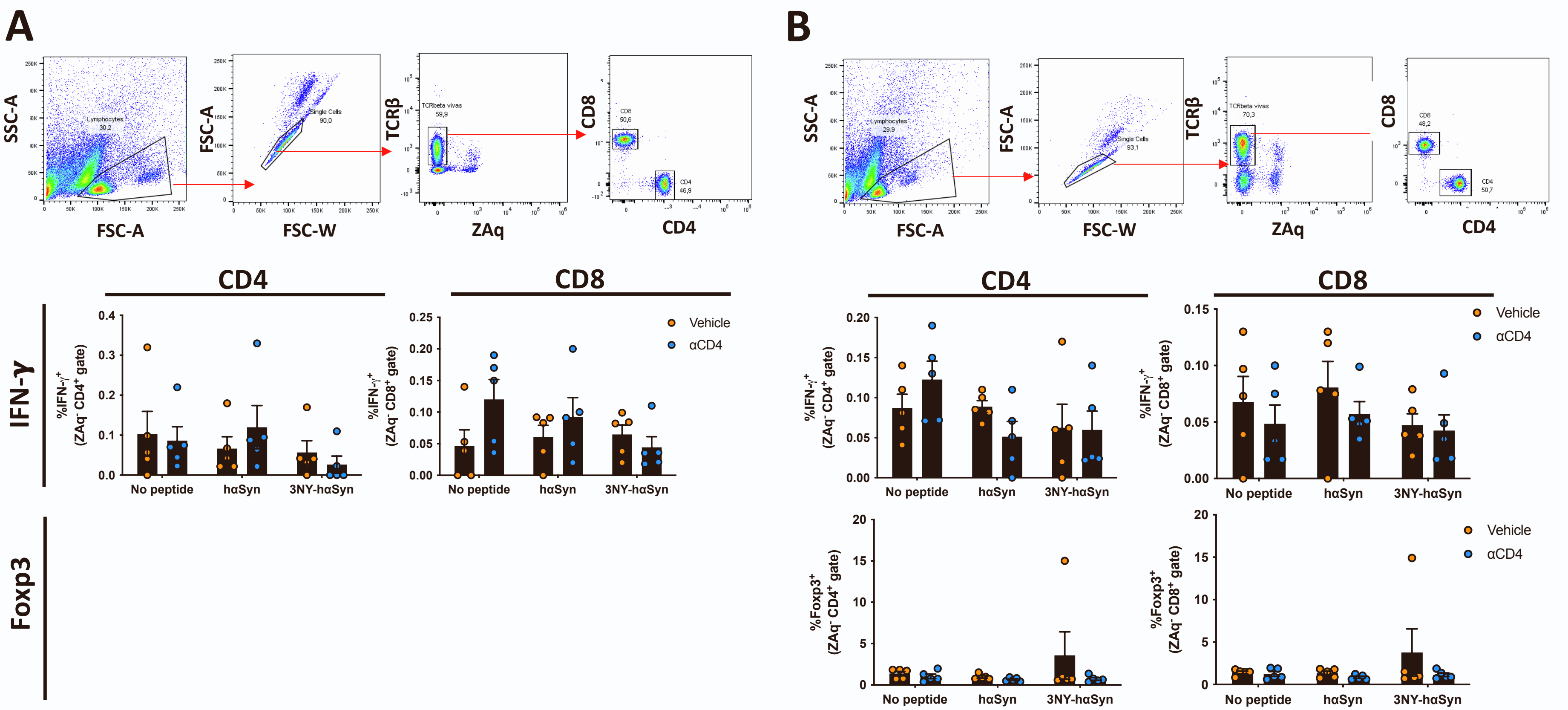

Figure S22. Analysing the T-cell response to αSyn-derived antigens in cervical and mesenteric lymph nodes upon transiently depleting CD4⁺ T-cells in *SNCA* mice treated with CAR-Treg. Related to Figure 5. Eight weeks old *SNCA* mice received three injections of a depleting anti-CD4 mAb (i.p., 100 μg/injection; blue symbols) or vehicle (orange symbols) separated for 24 h between injections. At week 10 of age, mice were treated with i.v. CAR-Treg (10^5 cells/mouse). At the week 20 of age, mononuclear cells were isolated from the cervical (**A**) mesenteric (**B**) lymph nodes. Cells were stimulated with peptides derived from unmodified hαSyn, 3NYhαSyn, or without peptides, and 24 h later the extent of IFN**γ** and Foxp3 expression in the CD4+ and CD8+ T-cell populations was analysed by intracellular immunostaining followed by flow cytometry. Values are the percentage of IFN**γ**+ or Foxp3+ cells in the $CD4^+$ TCRβ^+ ZAq^- and $CD8^+$ TCRβ^+ ZAq^- population. Top panels show the gating strategy and bottom panels show the quantification. Each symbol represents an individual mouse (n = 5 mice per group). Mean ± SEM are shown. No significant differences were found using the multiple t-test.

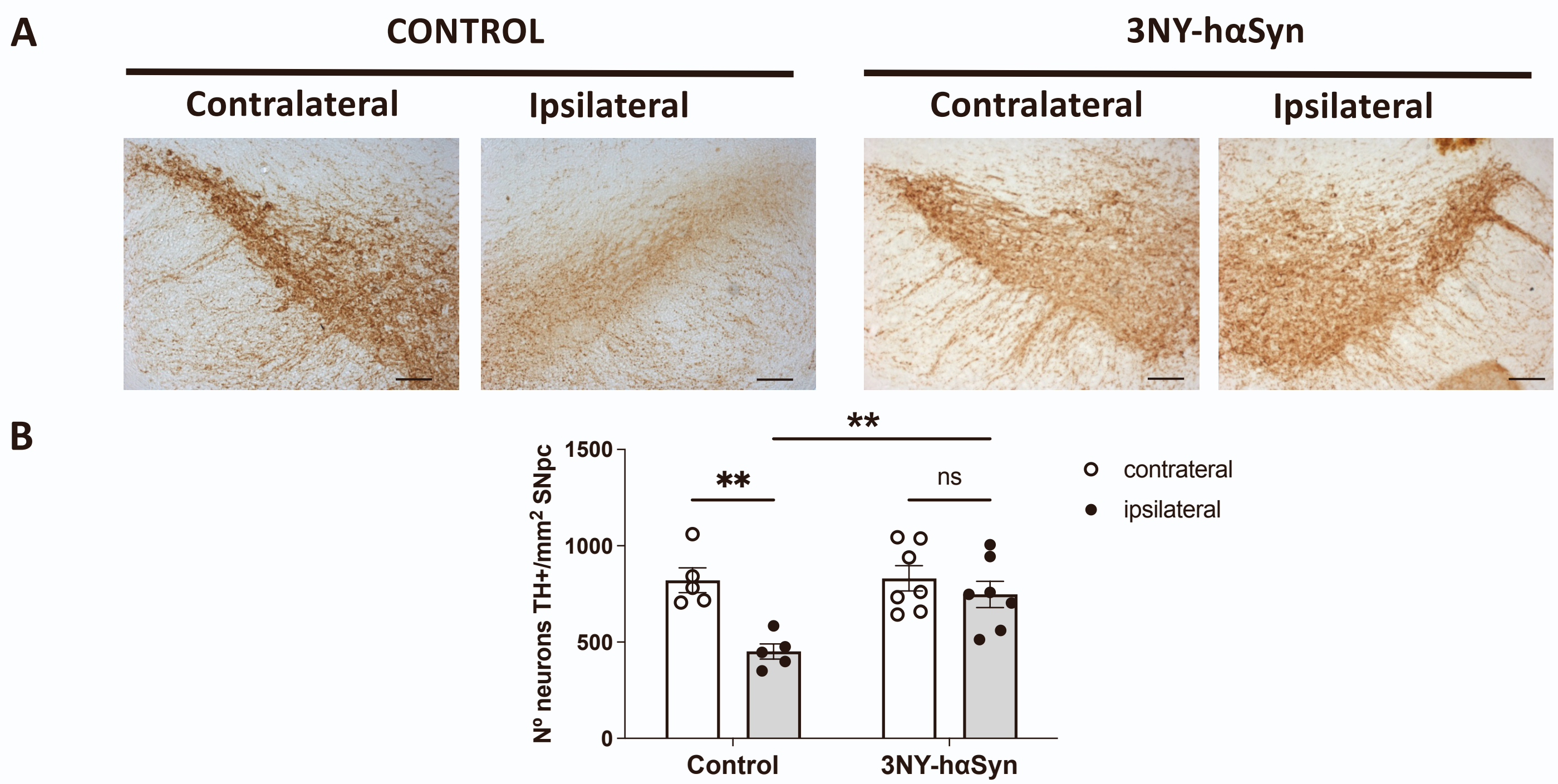

Figure S23. Vaccination with 3NYαSyn exerts a therapeutic effect attenuating neurodegeneration in the AAV-hαSyn mouse model. Related to Figure 6. C57BL/6 mice received unilateral stereotaxic injections of AAV-hαSyn (10^{10} vg/mice) in the right SN (ipsilateral). Three weeks later, mice were s.c. Immunised with complete Freund's adjuvant (CFA) containing 10 µg 3NY-hαSyn peptide (3NY-hαSyn group) or with CFA alone (Control group). Five-weeks after the first immunisation, mice in the 3NY-hαSyn group received a second s.c. immunisation with 10 µg 3NY-hαSyn peptide in incomplete Freund's adjuvant (IFA), while the control group received a s.c. injection of IFA alone. Seven-weeks after the second immunisation, mice were euthanised and the extent of dopaminergic neuron loss was determined by immunohistochemical (IHC) analysis of TH in the SN. (**A**) Representative images obtained for IHC analyses of TH are shown. Left SN were analysed as internal controls (contralateral). Scale bars, 80 **µ**m. (**B**) Quantification of TH+ neurons per area in the SNpc. **, $p<0.01$ by two-ways ANOVA followed by the Sidak's multiple comparison test; n = 5-7 mice per group. ns, not significant.

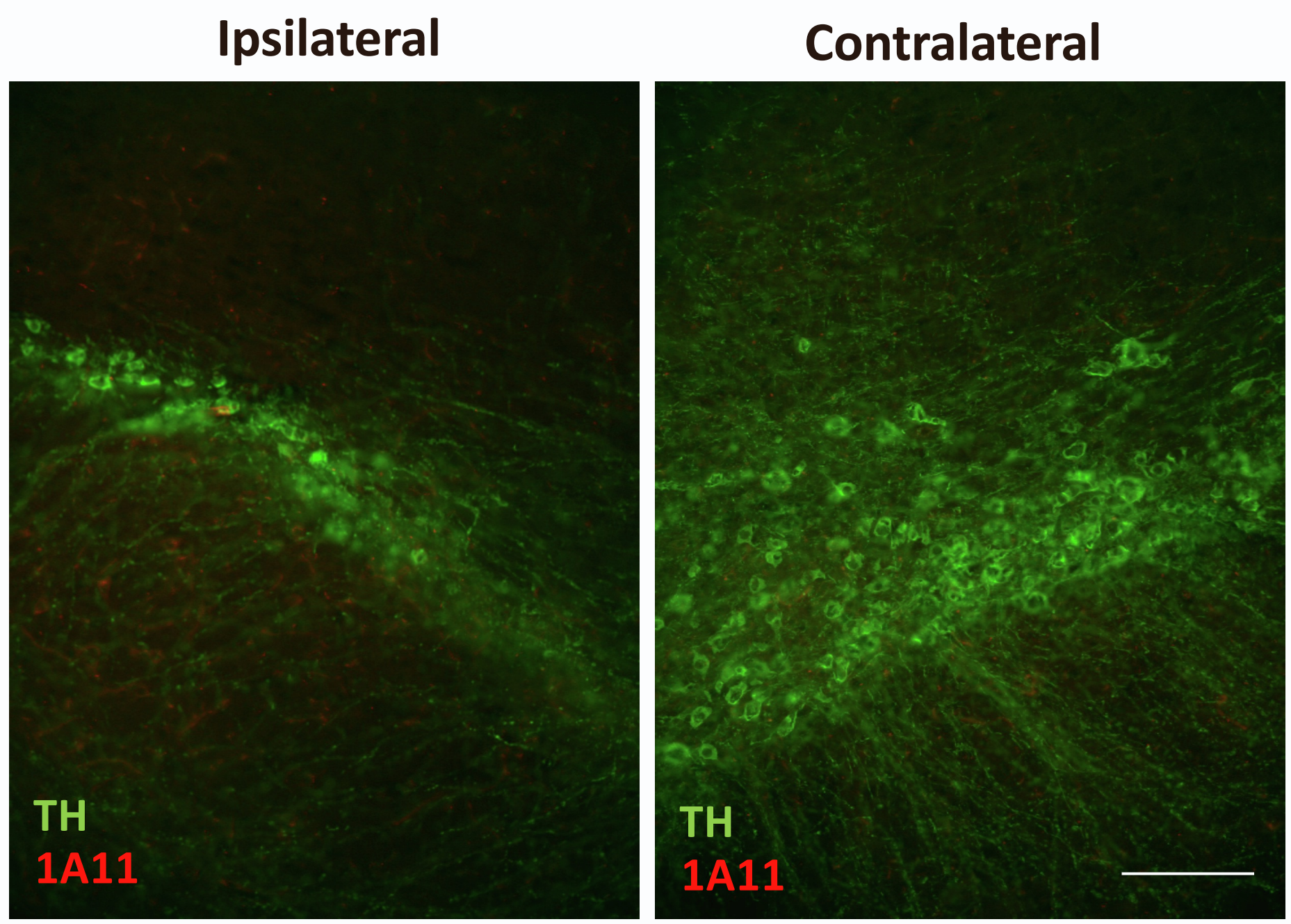

Figure S24. Immunoreactivity of 1A11 mAb in the SN of mice treated with AAV-hαSyn. Related to Figure 6. Wild-type C57BL/6 mice were anesthetized and received the stereotaxic inoculation of AAV encoding for human α-synuclein under the control of the chicken beta-actin (CBA) promoter (10^{10} vg/mouse) into the right substantia nigra (SN). After 15 weeks, mice were sacrificed and incubated with 1A11 mAb (1A11) followed by AlexaFluor546-coupled anti-mouse secondary antibody in brain slices containing the area of the SN. TH was also immunostained with a rabbit anti-TH pAb followed by an AlexaFluor488-coupled anti-rabbit secondary antibody. TH and 1A11 associated fluorescence was analysed by epifluorescence microscopy. Representative images of merge staining of TH (green) and 1A11 (red) are shown. Bar, 100 µm.

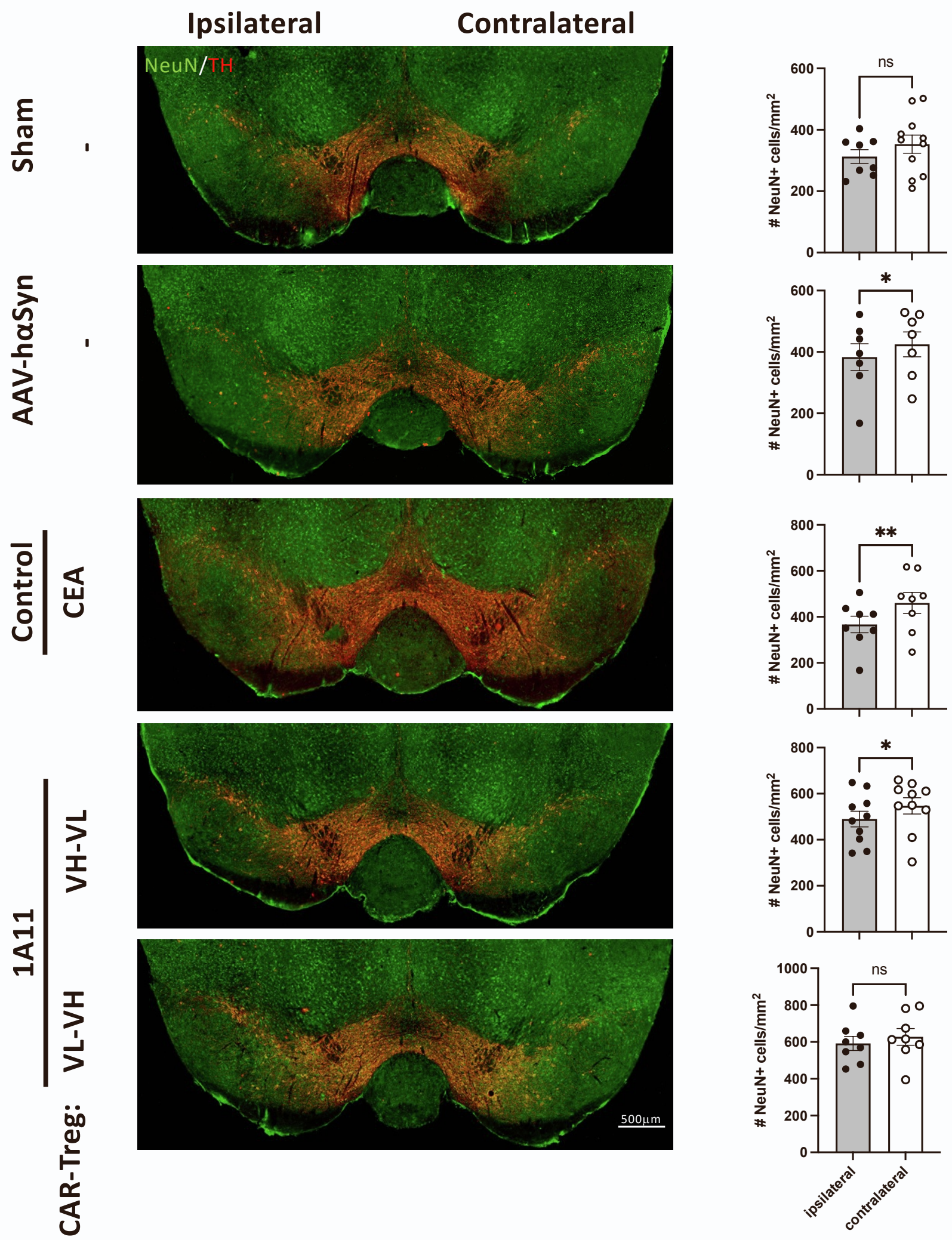

Figure S25. The reduction of the number of TH+ cells in the SN of mice treated with AAV-hαSyn and receiving CAR-1A11 T-cell therapy correlates with decreased number of NeuN+ neurons. Related to Figure 6. Wild-type C57BL/6 mice received the stereotaxic inoculation of AAV encoding human **α**-Synuclein (AAV-h**α**Syn; 10^{10} vg/mouse) or vehicle (sham) into the right substantia nigra (SN). Three weeks after the surgery, mice received the i.v. injection of CAR-Treg ($3x10^4$ cells per mouse) expressing the CAR1A11 in the VH-VL or VL-VH conformations. As a control group, some mice received the i.v. injection of CAR-Treg ($3x10^4$ cells per mouse) expressing the CAR-CEA (control CAR). Sixteen weeks after the stereotaxic surgery, nigral sections were co-immunostained for TH (red) and the pan-neuronal marker NeuN (green), and the number of NeuN+ cells were quantified in the SN (TH+ area). Left panels show representative images. Bar, 500 **μ**m. Right panels show the quantification of NeuN+ cells in the TH+ areas in the ipsilateral (grey bars) and the contralateral (white bars) SN. Data represent the mean with SEM from 2 slices per mouse from 4-5 mice per group. *, p<0.05; **, p<0.01 by paired Student's t-test. ns, non-significant.

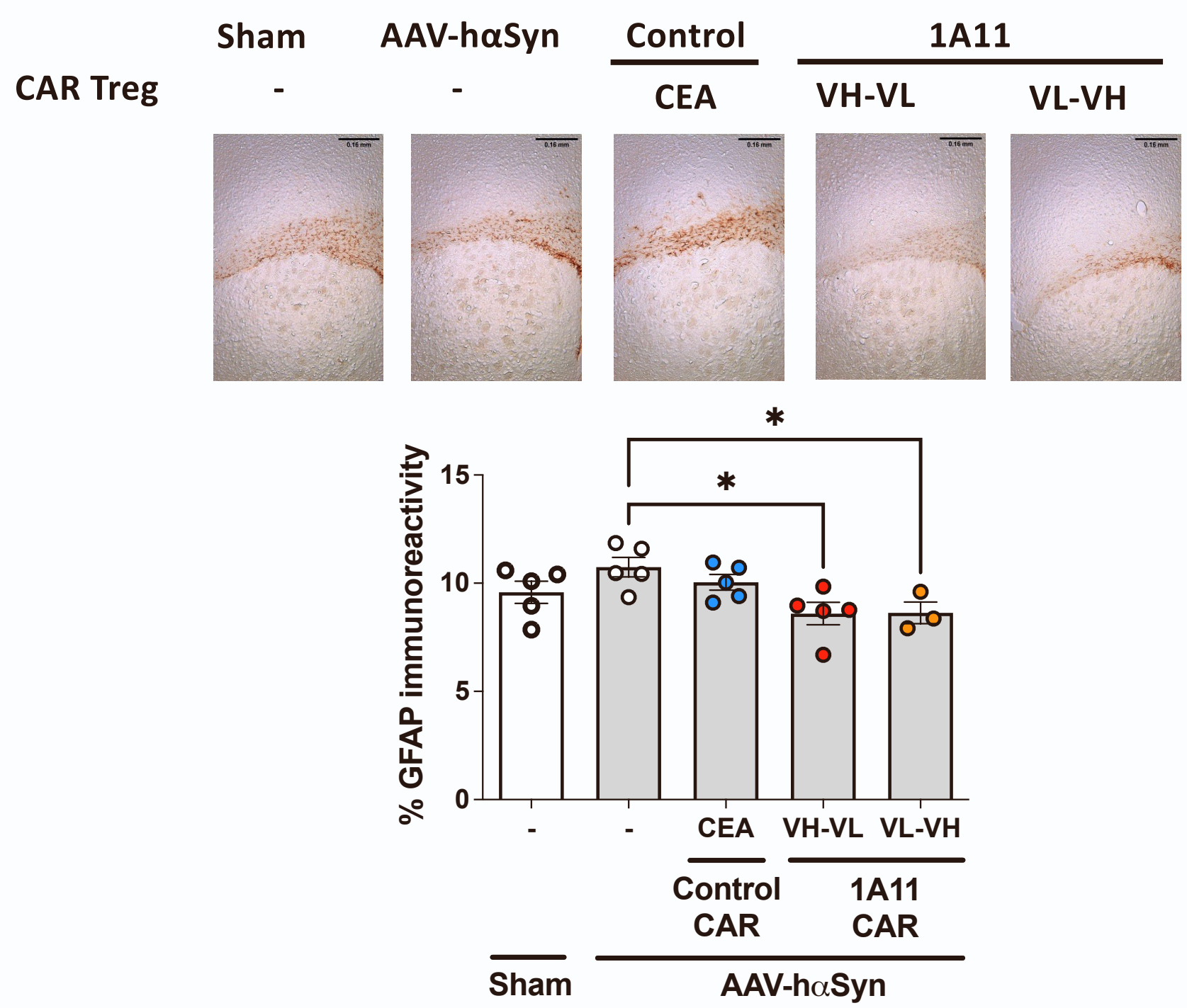

Figure S26. Analysis of astrogliosis in the striatum of mice treated with AAV-hαSyn and receiving CAR-1A11 T-cell therapy. Related to Figure 6. Wild-type C57BL/6 mice received the stereotaxic inoculation of AAV encoding human α-Synuclein (AAV-hαSyn; 10^{10} vg/mouse; grey bars) or vehicle (sham; white bar) into the right substantia nigra (SN). Three weeks after the surgery, mice received the i.v. injection of CAR-Treg ($3x10^4$ cells per mouse) expressing the CAR1A11 in the VH-VL (red symbols) or VL-VH (orange symbols) conformations. As a control group, some mice received the i.v. injection of CAR-Treg ($3x10^4$ cells per mouse) expressing the CAR-CEA (control CAR; blue symbols). Sixteen weeks after the stereotaxic surgery, mice were sacrificed and astrogliosis was evaluated by immunohistochemical analysis of GFAP in the striatum. Top panels show representative images for each experimental group. Scale bars, 160 µm. Bottom panel show the quantification of the density of GFAP immunoreactivity. Data represent the mean with SEM from 5 mice per group. *, $p<0.05$ by one-way ANOVA followed by Dunnett's *post-hoc* test.

Table S1. Peptides used in the study. Related to figure 1 A and B.

Name	Sequence	Provider
hαSyn111-140	GILEDMPVDPDNEAYEMPSEEGYQDYEPEA	Genscript
3NY-hαSyn	GILEDMPVDPDNEAY*EMPSEEGY*QDY*EPEA	Genscript
NY125-hαSyn	GILEDMPVDPDNEAY*EMPSEEGYQDYEPEA	Genscript
NY133-hαSyn	GILEDMPVDPDNEAYEMPSEEGY*QDYEPEA	Genscript
NY136-hαSyn	GILEDMPVDPDNEAYEMPSEEGYQDY*EPEA	Genscript
NY125,133-hαSyn	GILEDMPVDPDNEAY*EMPSEEGY*QDYEPEA	Genscript
NY125,136-hαSyn	GILEDMPVDPDNEAY*EMPSEEGYQDY*EPEA	Genscript
NY133,136-hαSyn	GILEDMPVDPDNEAYEMPSEEGY*QDY*EPEA	Genscript
3NY-scrambled	LQSEVEY*GAEAEDMGEPIPMPY*DNY*CDPEED	Genscript
3NY-scrambled	LQSEVEY*GAEAEDMGEPIPMPY*DNY*CDPEED	Biosynthesis
5FAM-hαSyn	#GILEDMPVDPDNEAYEMPSEEGYQDYEPEA	Genscript
5FAM-NY133,136-hαSyn	#GILEDMPVDPDNEAYEMPSEEGY*QDY*EPEA	Genscript
5FAM-3NY-hαSyn	#GILEDMPVDPDNEAY*EMPSEEGY*QDY*EPEA	Genscript

Y*, Indicates nitrated tyrosine; #, indicates 5FAM coupled in the N-terminus.

Table S2. Demographic features and disease activity of Parkinson's disease patients. Related to figure 1 D and E.

Gender (Female/Male)	11 (3/8)
Age	69,4 ± 12,3
MoCA test[1]	26,2 ± 4,3
UPDRS[2]	40,2 ± 24,9
Hoehn and Yahr[3]	2,1 ± 1,2
Schwab & England[4]	77 ± 23

[1] Montreal Cognitive Assessment (scale 0-30); [2] Unified Parkinson's Disease Rating Scale (scale 0-199); [3] Modified Hoehn and Yahr scale (scale 0-5); [4] Schwab & England activities of daily living scale (%).
